# Superior efficacy of co-targeting GFI1/KDM1A and BRD4 against AML and post-MPN secondary AML cells

**DOI:** 10.1038/s41408-021-00487-3

**Published:** 2021-05-20

**Authors:** Warren Fiskus, Christopher P. Mill, Behnam Nabet, Dimuthu Perera, Christine Birdwell, Taghi Manshouri, Bernardo Lara, Tapan M. Kadia, Courtney DiNardo, Koichi Takahashi, Naval Daver, Prithviraj Bose, Lucia Masarova, Naveen Pemmaraju, Steven Kornblau, Gautam Borthakur, Guillermo Montalban-Bravo, Guillermo Garcia Manero, Sunil Sharma, Matthew Stubbs, Xiaoping Su, Michael R. Green, Cristian Coarfa, Srdan Verstovsek, Joseph D. Khoury, Christopher R. Vakoc, Kapil N. Bhalla

**Affiliations:** 1 The University of Texas M.D. Anderson Cancer Center, Houston, TX USA; 2 Department of Cancer Biology, Dana-Farber Cancer Institute and Department of Biological Chemistry and Molecular Pharmacology, Harvard Medical School, Boston, MA USA; 3 Department of Molecular and Cellular Biology Baylor College of Medicine, Houston, TX USA; 4 The Translational Genomics Research Institute (TGen), Phoenix, AZ USA; 5 Incyte Corporation, Wilmington, DE USA; 6 Cold Spring Harbor Laboratory, Cold Spring Harbor, NY USA

**Keywords:** Acute myeloid leukaemia, Targeted therapies

## Abstract

There is an unmet need to overcome nongenetic therapy-resistance to improve outcomes in AML, especially post-myeloproliferative neoplasm (MPN) secondary (s) AML. Studies presented describe effects of genetic knockout, degradation or small molecule targeted-inhibition of GFI1/LSD1 on active enhancers, altering gene-expressions and inducing differentiation and lethality in AML and (MPN) sAML cells. A protein domain-focused CRISPR screen in LSD1 (KDM1A) inhibitor (i) treated AML cells, identified BRD4, MOZ, HDAC3 and DOT1L among the codependencies. Our findings demonstrate that co-targeting LSD1 and one of these co-dependencies exerted synergistic in vitro lethality in AML and post-MPN sAML cells. Co-treatment with LSD1i and the JAKi ruxolitinib was also synergistically lethal against post-MPN sAML cells. LSD1i pre-treatment induced GFI1, PU.1 and CEBPα but depleted c-Myc, overcoming nongenetic resistance to ruxolitinib, or to BETi in post-MPN sAML cells. Co-treatment with LSD1i and BETi or ruxolitinib exerted superior in vivo efficacy against post-MPN sAML cells. These findings highlight LSD1i-based combinations that merit testing for clinical efficacy, especially to overcome nongenetic therapy-resistance in AML and post-MPN sAML.

## Introduction

During complete remission in AML, AML stem/progenitor cells (LSCs) in the measurable residual disease (MRD) resist differentiation, retain leukemia-initiating potential and mediate relapse of AML^1–3^. This phenotype of LSCs is mainly orchestrated by dysregulated super-enhancers (SEs)/enhancers (Es) and the ensuing dysregulated transcriptome/proteome, which directly results from genetic alterations and/or perturbed levels and activity of epigenetic and transcriptional regulators in AML^4–6^. SEs/Es of myeloid lineage transcription factors (TFs) and of their target genes in AML LSCs are characterized by histone H3 lysine (K) 27 acetyl (H3K27Ac) and H3K4 mono-methylation (H3K4Me1) marks, as well as by high occupancy with BRD4 and histone acetyl transferase CBP/p300^7–9^. Among the key LSC-specific TFs that regulate cell growth, differentiation and survival of AML stem cell differentiation are RUNX1, PU.1, CEBPα, c-Myb, and c-Myc^10–13^. LSD1 (lysine specific demethylase 1; KDM1A) is a FAD-dependent amine-oxidase family member and a component of the co-repressor complexes involving HDAC1/2 and CoREST (RCOR1) or NuRD (MTA2)^14^. LSD1 contains a conserved N-terminal SWIRM domain (100 AA) and C-terminal amine oxidase-like (AOL) catalytic domain^14,15^. The AOL domain binds to FAD and the methylated H3K4 substrate, causing demethylation of mono and dimethyl histone H3 lysine 4 (H3K4Me1 and H3K4Me2)^15,16^. Two antiparallel α-helices divide and project away from the AOL domain as the Tower domain, which provides the binding interface with CoREST (RCOR1 and HDAC1/2) and allosterically regulates the catalytic activity and stability of LSD1^14–16^. In addition to H3K4, LSD1 also demethylates TP53, E2F1, and DNMT1^17,18^. LSD1 interacts with GFI1/1B, a zinc finger transcriptional repressor and master regulator of normal and malignant lineage development and differentiation in hematopoiesis^18–21^. GFI1/1B contains an N-terminal, 20 AA SNAG domain and six C-terminal Zn fingers^22^. LSD1 binds to the methylated lysine in the ^8^KSKK^11^ motif in the SNAG (Snail/GFI1) domain, and thereby helps recruit RCOR1-HDAC1/2 co-repressor complex to mediate transcriptional repression and differentiation block due to GFI1 activity in AML stem/progenitor cells^19–22^. GFI1/1B can be co-immunoprecipitated with LSD1 and CoREST and exhibits overlapping DNA-binding sites at enhancers (by ChIP-Seq analyses) of key myeloid/monocyte differentiation–regulatory genes^21,23^. LSD1 inhibitors (LSD1i) disrupt GFI1/1B interaction with LSD1-CoREST, inducing differentiation of AML blast progenitor cells^17,21,23–25^. Importantly, CRISPR-suppressor scanning revealed that enzymatic activity of LSD1 was not required for blocked AML differentiation and survival^26^. GFI1/1B interaction with LSD1 and CoREST also causes demethylation of K372 on TP53, thereby inactivating TP53^17,21^. Notably, LSD1 is over-expressed in the stem/progenitor versus differentiated sub-types of AML^17,21^, and GFI1 expression is a documented prognostic factor in MDS/AML^27^. LSD1i treatment also increased chromatin accessibility and binding of SPI1 and CEBPα at their target Es/promoters^28^. Knockdown by shRNA or treatment with either the irreversible tranylcypromine (TCP)-derivative LSD1i or the reversible LSD1i SP2509 disrupted LSD1-binding to CoREST and GFI1/1B, induce differentiation markers (CD86 and CD11b) and morphologic differentiation, repress colony growth, as well as sensitize AML blast progenitor cells (BPCs) to all-trans retinoic acid (ATRA)^23,25,29–32^. Co-treatment with LSD1i and cytarabine, DNA hypomethylating agents, or inhibitor of HDACs, FLT3, DOT1L or BCL2, was shown to exert synergistic lethality in AML expressing MLL fusion protein^25,30^. However, these studies did not interrogate the activity of LSD1i and LSD1i-based combinations in post-MPN sAML blast progenitor cells (BPCs). In present studies, utilizing for the first time CRISPR-Cas9, or LSD1-FKBP12^F36V^ and dTAG-13, we demonstrate that knockout (KO) or degradation of LSD1 inhibits growth and induces differentiation of AML BPCs with or without expression of MLL fusion oncoproteins, and of post-MPN sAML BPCs. While disrupting the binding of LSD1 to CoREST and GFI1, treatment with irreversible LSD1i also attenuated c-Myc levels, but induced expressions of GFI1, PU.1, p21, and CD11b, inhibiting in vitro growth, inducing differentiation/cell lethality, as well as extending survival in immune-depleted mice engrafted with AML or sAML. Utilizing a domain-focused CRISPR-Cas9 sgRNA screen followed by LSD1i treatment, present studies also demonstrate co-dependencies, including BRD4, in AML cells. Whereas co-treatment with an LSD1i and BETi (OTX015) synergistically induced lethality in PD AML including sAML BPCs, pre-treatment with LSD1i sensitized JAKi-resistant post-MPN sAML cells to ruxolitinib and BETi P/R cells to BETi-induced apoptosis^33^. Co-treatment with LSD1i and BETi (OTX015) also inhibited AML cell burden and improved median survival of NSG mice engrafted with AML or post-MPN sAML cells.

## Results

### Biologic effects of CRISPR-Cas9-mediated LSD1 knockout or dTAG-13-mediated degradation of LSD1 in AML cells

**Fig. 1:**
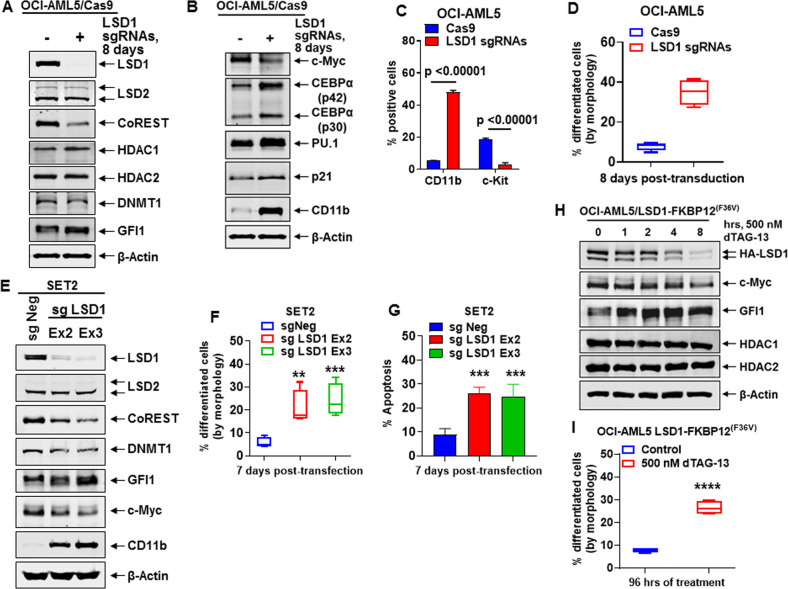
Knockout of LSD1 by CRISPR/Cas9 depletes c-Myc, and derepresses myeloid differentiation gene CD11b in AML and sAML cells. **A, B** Representative immunoblot analysis of OCI-AML5 Cas9- expressing cells transduced with two lentiviral sgRNAs against LSD1 and incubated for 8 days. **C** Expression of c-Kit and CD11b (assessed by flow cytometry) in OCI-AML5 Cas9 and LSD1 knockout cells 8 days post-transduction. Mean of two independent experiments performed in duplicate + S.D. Significance determined by a two-tailed, unpaired *t*-test. **D** Morphologic differentiation (% myelocytes and metamyelocytes or bands by morphologic features of hematoxylin & eosin-stained cytospun cells) of OCI-AML5 Cas9 and LSD1 knockout cells 8 days post-transduction. Mean of two independent experiments performed in duplicate + S.D. **E** Representative immunoblot analysis of SET2 cells transfected with two sgRNAs against LSD1 and incubated for 7 days. **F, G** Induction of morphologic differentiation (% myelocytes and metamyelocytes or bands by morphologic features of hematoxylin & eosin-stained cytospun cells) and apoptosis in SET-2 LSD1 knockout cells 7 days post-transfection. Mean of two independent experiments performed in duplicate + S.D. **p < 0.01, ***p < 0.005 compared to sgNeg-transfected SET-2 cells (determined by a two-tailed, unpaired *t*-test). **H** Immunoblot analysis of OCI-AML5/LSD1-FKBP12(^F36V^) cells treated with 500 nM of dTAG-13 for the indicated times. **I** OCI-AML5/LSD1-FKBP12(^F36V^) cells were treated with 500 nM of dTAG-13 for 96 h. At the end of treatment, differentiation was assessed by cell morphology, as described above. Mean of three experiments + S.D. ****p < 0.001 compared to untreated control cells (determined by a two-tailed, unpaired *t*-test).

We first determined effects of CRISPR-Cas9-mediated knockout (KO) of LSD1 on the CoREST complex and on GFI1 and its targets in AML OCI-AML5 and post-MPN sAML SET-2 cells. Fig. 1A demonstrates that 8-days following transduction of two gRNAs targeting exon 2 and 3 of LSD1 into OCI-AML5 cells, stably transduced with Cas9, LSD1 was profoundly depleted, associated with reduction in protein levels of CoREST and slightly of DNMT1, increased GFI1, and unaltered HDAC1/2 and LSD2 levels. LSD1-KO also attenuated c-Myc levels, while increasing protein levels of PU.1, CEBPα, p21 and CD11b (Fig. 1B). Alterations in the levels of these proteins were associated with decline in % of cells in cell cycle S-phase and increase in % of G1-phase cells, accompanied by augmentation in % of cells expressing CD11b that also displayed morphologic features of differentiation (% myelocytes and metamyelocytes or bands by morphologic features of hematoxylin & eosin-stained cytospun cells), while reducing % of cells expressing c-KIT (Figs. 1C, D and S1A). LSD1-KO via CRISPR-Cas9 in post-MPN sAML SET-2 cells, was also accompanied by decline in the protein levels of LSD1, CoREST, c-Myc, and DNMT1, but increased protein levels of GF11, PU.1 and CEBPα (Figs. 1E and S1B). LSD1-KO also inhibited in vitro growth and increased % of morphologically-differentiated (% myelocytes and metamyelocytes or bands by morphologic features of hematoxylin & eosin-stained cytospun cells) SET-2 cells with increased CD11b but reduced c-KIT expression (Figs. 1F, S1C, D), along with significant increase in % apoptotic cells (Fig. 1G). Following CRISPR-Cas9-mediated LSD1-KO, perturbed protein expressions could be documented only 5–8 days later. Therefore, we employed the dTAG system^34^ to degrade LSD1-FKBP12^(F36V)^ in OCI-AML5 cells following knockout of the endogenous LSD1 to assess within hours loss of LSD1 and the downstream biological consequences. As shown in Figs. 1H, S1E–H, 4–24 h after adding dTAG-13, protein levels of LSD1, CoREST, and c-Myc declined, while GFI1, PU.1, RUNX1, p21, p27, and CD11b levels were induced in OCI-AML5 cells. dTAG-13 treatment did not affect LSD2, HOXA9, and Meis1 levels in OCI-AML5 cells (Figs. S1G, H). Four days after dTAG-13 addition, an increase in the % of cells expressing CD11b and displaying morphologic features of differentiation was observed (Figs. 1I and S1I).

### Effect of LSD1 knockout on enhancer activity and transcriptome in AML cells

**Fig. 2:**
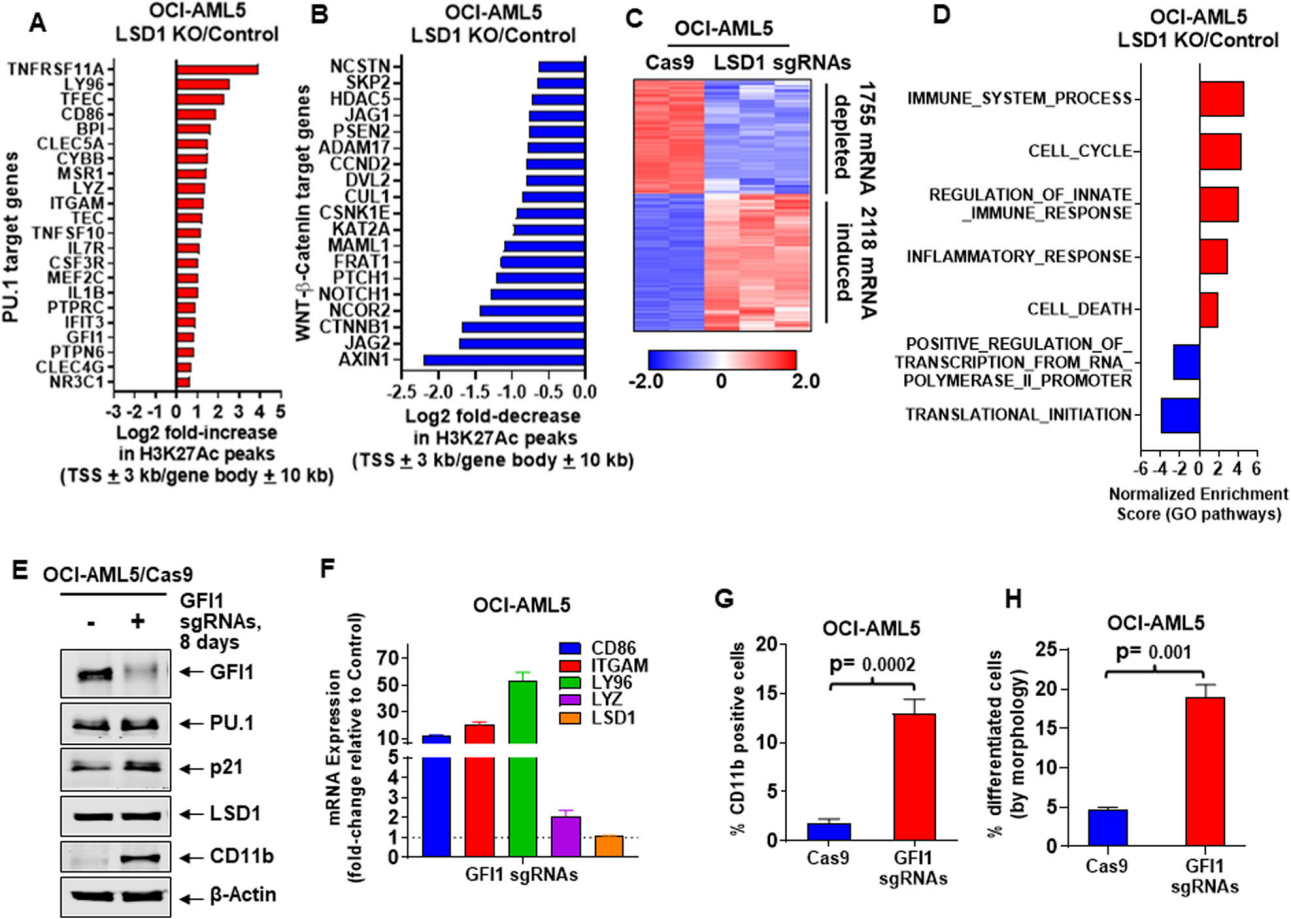
Knockout of LSD1 alters chromatin accessibility, augments H3K27Ac occupancy on PU.1 target and WNT-β-Catenin target genes, and knockout of GFI1 induces myeloid differentiation genes, LY96 and ITGAM (CD11b) and morphologic differentiation of AML cells. **A** Log2 fold-change in H3K27Ac ChIP-Seq peaks (TSS + 3 kb or gene body + 10 kb) for PU.1 target genes (from the MSigDB database) in LSD1 knockout compared to Cas9-only-expressing OCI-AML5 cells. **B** Log2 fold-change in H3K27Ac ChIP-Seq peaks (TSS + 3 kb or gene body + 10 kb) in WNT-β-Catenin target genes (from HALLMARK datasets in the MSigDB database) in LSD1 knockout compared to Cas9-only-expressing OCI-AML5 cells. **C** RNA-Seq analysis was performed on OCI-AML5 LSD1 knockout (biologic triplicates) and Cas9-only-expressing (biologic duplicates) cells 8 days post-transduction. Heat map shows the number of mRNAs depleted or induced > 1.25-fold and *p*-value < 0.05 in the LSD1 knockout versus Cas9-only-expressing cells. **D** Gene set enrichment analysis was performed with RNA expression changes in OCI-AML5 LSD1-KO cells compared to GO pathways. The *q*-values are < 0.1 in all comparisons. **E** Representative immunoblot analysis of OCI-AML5 Cas9-expressing cells transduced with two lentiviral sgRNAs against GFI1 and incubated for 8 days. **F** Relative mRNA expression analysis of OCI-AML5 GFI1 knockout cells compared to Cas9-only-expressing cells. GAPDH expression was utilized as the normalization control. **G** Expression of CD11b (assessed by flow cytometry) in OCI-AML5-Cas9 and GFI1 knockout cells 8 days post-transduction. Mean of two independent experiments performed in duplicate + S.D. Significance determined by a two-tailed, unpaired *t*-test. **H** Morphologic differentiation (% myelocytes and metamyelocytes or bands by morphologic features of hematoxylin & eosin-stained cytospun cells) of OCI-AML5 GFI1 knockout cells compared to Cas9-only-expressing cells 8 days post-transduction. Mean of two independent experiments performed in duplicate + S.D. Significance determined by a two-tailed, unpaired *t*-test in GraphPad V8.

Utilizing ChIP-Seq analysis, we next determined the H3K27Ac signal-density at specific loci in LSD1-KO compared to the control OCI-AML5 cells. Increases in peak-densities were seen in PU.1 targets, while the peak-densities decreased on WNT-β-catenin targets (Figs. 2A, B). H3K27Ac signal-density also decreased at the MYC and CDK6 loci, whereas ITGAM, LY96, and LYZ gene-loci demonstrated increased H3K27Ac occupancy (Fig. S2A). Consistent with this, H3K27Ac peaks also declined on the chromatin of many c-Myc target genes (Figs. S2B, C). ChIP-Seq analysis following LSD1-KO also increased H3K27Ac and BRD4 peaks at the enhancer-promoter regions of GFI1 gene (Fig. S2D), accompanied by increased GFI1 levels in OCI-AML5 cells (Fig. 1A). RNA-Seq analysis following LSD1-KO revealed the heat map of the up- or downregulated mRNAs (Fig. 2C), which included perturbations of PU.1 and CEBPα target-gene expressions (Fig. S2E, F)^33^. Gene set enrichment analyses (GSEA) of the perturbed mRNAs, according to GO and HALLMARK pathways, demonstrated significant positive normalized enrichment scores (NES) for gene-sets, including those of innate immune and inflammatory responses, apoptosis pathways, but negative NES for c-Myc, WNT-β-catenin and translation–initiation pathway genes (Figs. 2D and S2G). Utilizing two custom-designed, nonoverlapping primer sets and qPCR analysis, we also determined enhancer RNA (eRNA) abundance within the known enhancer regions in the MYC SE^33^. LSD1-KO in OCI-AML5 cells depleted eRNA expression from enhancers 3, 4, and 5 but not from enhancer 2 of MYC (Fig. S2H).

### Biologic outcome following depletion of GFI1 versus LSD1 in AML cells

Next, we compared effects of LSD1-KO with those of GFI1 knockout in OCI-AML5 cells. Utilizing two gRNAs designed against exon 2 and 3, CRISPR-Cas9-mediated knockout of GFI1 in OCI-AML5 cells resulted in marked depletion of GFI1, but not of LSD1, with concomitant increase in mRNA expressions of ITGAM, CD86, LY96 and LYZ, as well as increased PU.1, p21 and CD11b protein levels with significant increase in % of cells displaying morphologic differentiation (% increase in myelocytes and metamyelocytes) and expressing CD11b (Fig. 2E–H). Thus, similar to LSD1-KO, GFI1-KO also induced differentiation of AML cells. To compare gene-expression perturbations that accompany differentiation effects due to depletion of LSD1 versus GFI1, we conducted knockdown (KD) of GFI1 and LSD1 with 2 shRNA each, which also exhibited similar effects on differentiation and associated gene expressions in OCI-AML5 (Figs. S3A, B, S4A–F, and data not shown). Following treatment of OCI-AML5 cells with the shRNAs, RNA-Seq analyses showed that considerably greater numbers of mRNA were up or downregulated by LSD1 versus GFI1 shRNAs (Fig. S4G, H). LSD1-KD was associated with global increases in the histone H3K4Me2 and H3K4Me3 levels, as well as increased mRNA expressions of PU.1 and GFI1 targets, along with increased mRNA and/or protein expressions of GFI1, PU.1, p21, p27, CD11b, LY96 and CD86, but depletion of c-Myc (Figs. S4A–F, and S4I). Similar effects following LSD1-KD by shRNAs were also observed in AML MOLM13 cells expressing the MLL-AF9 fusion gene (S4J i–iv. and data not shown). Following KD of GFI1 or LSD1, GSEA of the RNA-seq-determined mRNA expression signatures also demonstrated positive NES for gene-sets including those of interferon α & γ, TNFα, IL2- or IL6-JAK-STAT3/5 and inflammatory pathway, while negative NES were observed for gene-sets including those of oxidative phosphorylation (Fig. S5A). In contrast, negative NESs included those for DNA repair, WNT-β-catenin, MYC and E2F pathway gene-sets, but only in the RNA-Seq signature of LSD1-KD (Fig. S5A). The Venn diagram in Fig. S5B demonstrates considerably more nonoverlapping, up- or downregulated gene-expressions following LSD1-KD, whereas Fig. S5C shows a number of gene expressions that were common and similarly perturbed, including those of known PU.1 targets^13,28^, following either LSD1 or GFI1 KD. The upregulation of PU.1 targets shown in Figure S5C is likely due to both increased expression of PU.1 and inhibition of GFI1-LSD1 axis. Overall, treatment with LSD1 shRNA induced a higher % of differentiation than GFI1 shRNA in OCI-AML5 cells, as determined by increase in % CD11b expression (LSD1 shRNA: 68.7% versus GFI1 shRNA: 30.4%) and morphologic features of myeloid differentiation (LSD1 shRNA: 71.7% and GFI1 shRNA: 33.9%). This suggests that the amount of differentiation induced by knockdown or knockout of LSD1 or GFI1 in AML cells best correlated with increased expression of PU.1 and p21 but depletion of c-Myc. Notably, 96 h post treatment with shRNA to LSD1 or GFI1, a small (<20%) but similar % of apoptotic OCI-AML5 cells were observed (% annexin V-positive cells).

### LSD1 inhibitors disrupt binding of LSD1 to CoREST and GFI1, inducing GFI1 levels, differentiation and cell lethality in AML cells

**Fig. 3:**
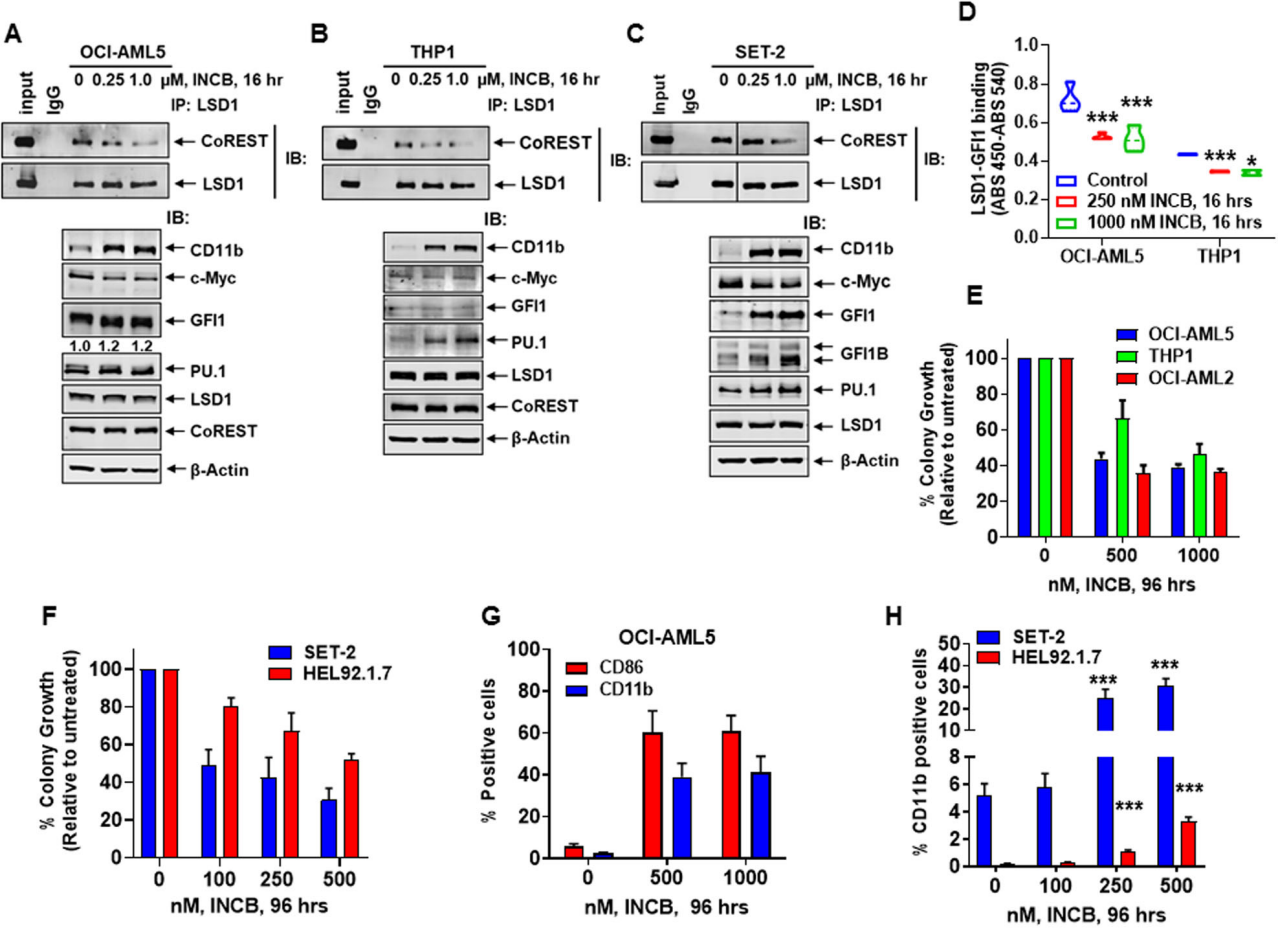
Treatment with LSD1 inhibitor (LSD1i) disrupts binding of LSD1 to CoREST and GFI1, attenuates colony growth, and induces differentiation in AML cells. **A–C** Immunoprecipitation and immunoblot analysis of OCI-AML5, THP1, and SET-2 cells treated with INCB059872 for 16 h. **D** Reduced binding of LSD1 and GFI1 (determined by sandwich ELISA assay) following 16 h of treatment with INCB059872. Mean of two independent experiments performed in duplicate + S.D. *p < 0.05, ***p < 0.005 (determined by a two-tailed, unpaired *t*-test). **E, F** OCI-AML5, OCI-AML2, THP1, SET-2, and HEL92.1.7 cells were treated with the indicated concentrations of INCB059872 for 96 h. Then, cells were plated in Methocult media and incubated at 37 °C. Colony growth was assessed after 7–10 days. Mean of two independent experiments + S.D. **G, H** The % of CD86 and/or CD11b-positive OCI-AML5, SET2 and HEL92.1.7 cells was determined by flow cytometry following treatment with INCB059872 for 96 h. Mean of three experiments + S.E.M. ***p < 0.005 in INCB059872-treated cells compared to untreated cells (determined by a two-tailed, unpaired *t*-test in GraphPad V8).

We next determined effects of the tranylcypromine analog, irreversible LSD1 I inhibitors (LSD1i) INCB059872 (INCB) and ORY-1001 in AML and post-MPN sAML cells^25,35–37^. Co-immunoprecipitation (Co-IP) analyses demonstrated that treatment with INCB reduced binding of LSD1 with CoREST in the AML OCI-AML5, THP1, and in sAML SET-2 cells (Fig. 3A–C)^25^. Sandwich ELISA demonstrated that treatment with INCB decreased binding of LSD1 with GFI1 in the AML cells (Fig. 3D). This was associated with increase in protein levels of GFI1/1B and PU.1 and decline in c-Myc, without significant alterations in CoREST and LSD1 levels in the same cell lysates used for the Co-IP analyses (Fig. 3A–C, and data not shown). INCB treatment inhibited % colony growth of several AML (OCI-AML5, THP1 and OCI-AML2) and post-MPN sAML cell types (SET-2 and HEL92.1.7) (Fig. 3E, F), as well as increased % of AML cells expressing CD11b and CD86 (Fig. 3G, H). Exposure to ORY-1001 exerted similar effects in AML and sAML cells (Fig. S6A–F)^25^. To evaluate whether LSD1i induces differentiation in AML cells solely by disrupting interaction of LSD1 with GFI1, we determined the effects of KD of GFI1 with or without co-treatment with the LSD1i INCB. Fig. S7A–C demonstrate that treatment with INCB significantly enhanced morphologic differentiation induced by GFI1-KD in OCI-AML5 cells, associated with increased % of cells expressing CD11b and CD86 (*p* < 0.05). Although GFI1-KD blocked INCB-induced GFI1 levels, INCB treatment increased differentiation with greater decline in c-Myc and c-Myb levels (Fig. S7D). INCB treatment also augmented GFI1 KD-induced p21 and CD11b levels in OCI-AML5 cells (Fig. S7D). A similar effect of INCB treatment on CD11b induction due to GFI1-KO via CRISPR-Cas9 was also noted in OCI-AML5 cells (*p* < 0.01) (Fig. S7E). These findings suggest that although clearly a marker of LSD1i-induced AML differentiation, accompanying GFI1 induction is not mechanistically involved in AML cell differentiation due to LSD1i-mediated disruption of LSD1 and GFI1 interaction. They also suggest that both scaffold and enzymatic functions of LSD1, which are inhibited by INCB, may be involved in AML cell differentiation induced by INCB^38^.

### LSD1 inhibitor-mediated early alterations in epigenome/transcriptome in AML and post-MPN sAML cells

Since effects of LSD1-KD (utilizing shRNA) or LSD1-KO (via CRISPR-Cas9) on epigenome/transcriptome can only be evaluated several days after gene-editing, we next determined whether these effects also occur soon after a 16-h exposure of AML and post-MPN sAML cells to INCB. Following INCB treatment, ATAC-Seq analysis demonstrated large numbers of lost and gained peaks, including those gained in the bivalent poised enhancers, enhancers, active TSS (transcription start sites) and polycomb-repressed chromatin of OCI-AML5 cells (Fig. S8A, B)^39,40^. TF motifs in the open chromatin included those of CTCF, FOSL1, PU.1, RUNX1, IRF8 and c-Myc, and the gained peaks included those in GFI1 and PU.1-target genes (Fig. S8C–F). Similar gain of ATAC-seq peaks were also noted in INCB-treated SET-2 cells (Fig. S8G, H). Although H3K27Ac ChIP-seq analysis showed only a slight increase in H3K27Ac signal-tag density at active enhancers, ROSE plot highlighted active SEs of RUNX1, GFI1, BCL2, PU.1, IRF8 and SMYD3 in INCB-treated compared to untreated OCI-AML5 cells, accompanied by increased H3K27Ac occupancy at the chromatin of GFI1 and PU.1 target genes (Fig. S8I–K). Notably, INCB treatment also increased BRD4 occupancy at +/− 3 kb of TSSs, and especially at the GFI1 and PU.1-target genes (Fig. S8L–N). RNA-Seq analysis showed that INCB treatment up- and downregulated large numbers of mRNA expressions, with positive NESs for gene-sets including those for interferon α, inflammatory response and E2F-target genes, and negative NES for c-Myc-targets and oxidative phosphorylation gene-sets (Fig. S9A, B). INCB treatment also positively enriched for GFI1-targets, while negatively enriching for c-Myc targets (Fig. S9C, D). Similar positive or negative NESs for these gene sets were also noted for INCB-treated SET-2 cells (Fig. S9E). Compared to untreated, RNA-Seq analyses of INCB-treated OCI-AML5 and SET-2 cells also demonstrated increased mRNA expressions of GFI1, PU.1, and CEBPα target-genes (Fig. S9F–K). The target genes induced were different in OCI-AML5 versus SET2 cells, most likely because of their disparate cell biology due to their de novo AML versus post-MPN sAML derivation. Notably, although LSD1i treatment induced GFI1 expression, because it disrupted binding of CoREST-LSD1 to GFI1, LSD1i treatment prevented GFI1-mediated repression, causing derepression of GFI1 target genes associated with AML cell differentiation.

### Targetable dependencies detected by protein domain-focused CRISPR-Cas9 screen in LSD1i-treated versus untreated AML cells

**Fig. 4:**
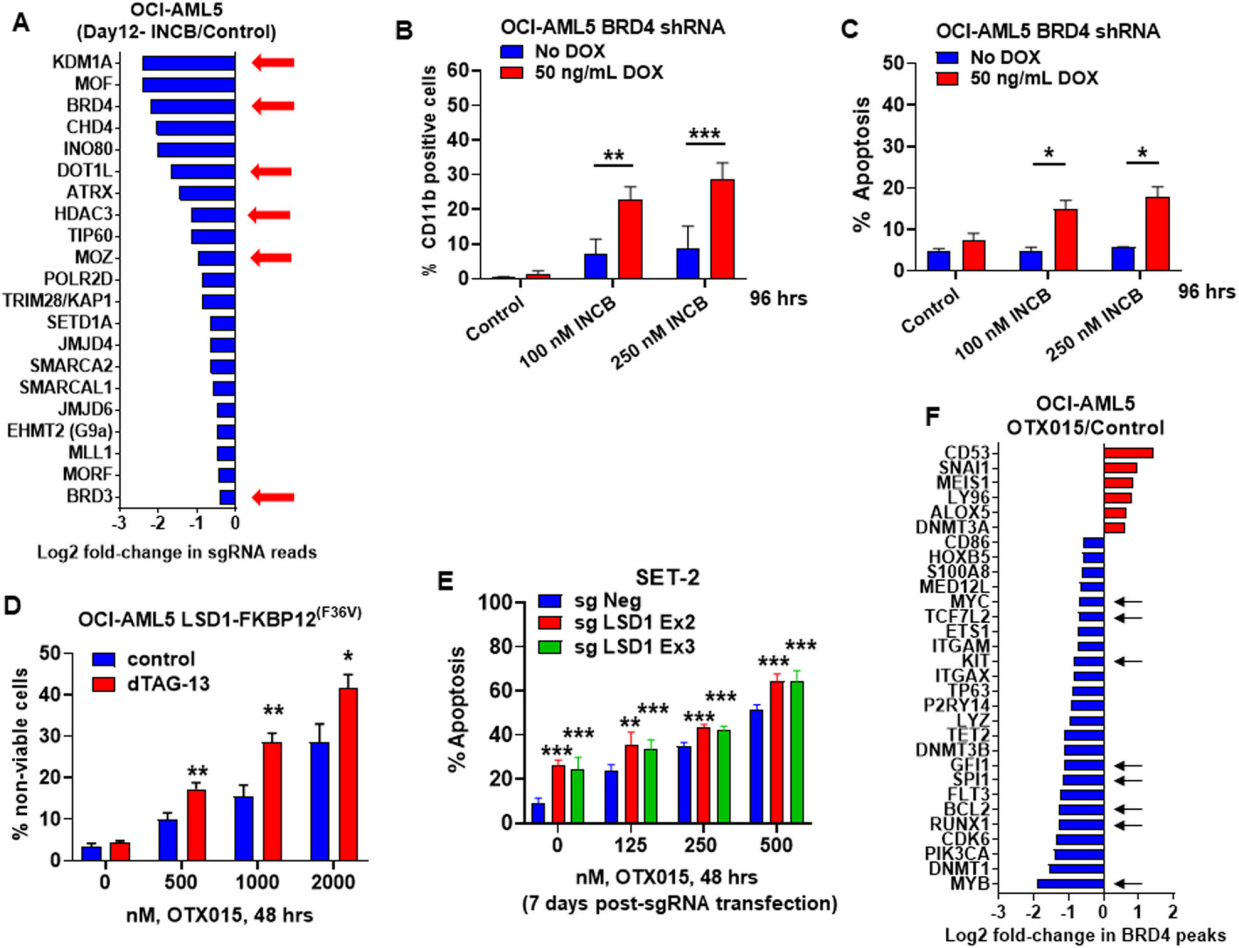
Synthetic lethal activity of treatment with INCB059872 and a CRISPR-Cas9-mediated domain-focused epigenetically targeted sgRNA library in AML cells. **A** OCI-AML5 Cas9 cells were transduced with a library of domain-specific sgRNAs against chromatin modifying proteins and incubated for 8 days. Then, 250 nM of INCB059872 was added and the cells were incubated for an additional 96 h. Live cells were harvested; genomic DNA was isolated and minimally amplified with primers flanking the sgRNA sequences. Sequencing libraries were generated and amplicon-seq was performed. The graph shows log2 fold-changes in selected sgRNAs which dropped out more due to treatment with INCB059872 than control cells at day 12 post-transduction. **B** OCI-AML5 cells with stable expression of DOX-inducible BRD4 shRNA were induced with DOX and treated with the indicated concentrations of INCB059872 for 96 h. Following this, the % of CD11b cells was determined by flow cytometry. Mean of two independent experiments + S.D. **p < 0.01, ***p < 0.005 as determined by a two-tailed, unpaired *t*-test. **C** OCI-AML5 cells with stable expression of DOX-inducible BRD4 shRNA were induced with DOX and then treated with the indicated concentrations of INCB059872 for 96 h. Following this, the % of annexin V-positive, apoptotic cells were determined by flow cytometry. Mean of two independent experiments + S.D. *p < 0.05 as determined by a two-tailed, unpaired *t*-test. **D** OCI-AML5 LSD1-FKBP12^(F36V)^ cells were treated with the indicated concentrations of OTX015 with and without 500 nM of dTAG-13 for 48 h. Then, the % of To-Pro-3 iodide positive cells were determined by flow cytometry. Mean of three experiments + S. E. M. *p < 0.05, **p < 0.01 compared to cells with no dTAG-13 treatment (determined by a two-tailed, unpaired *t*-test). **E** SET-2 cells were transfected with sgNeg or LSD1 sgRNAs and incubated for 5 days. Then, cells were treated with the indicated concentrations of OTX015 for 48 h and the % of apoptotic cells was determined by flow cytometry. Mean of two independent experiments performed in duplicate + S.D. **p < 0.01, ***p < 0.005 relative to sgNeg-transfected cells (determined by a two-tailed, unpaired *t*-test). **F** Log2 fold-changes in BRD4 peaks (determined by DiffReps analysis of BRD4 ChIP-Seq data) on LSD1 target genes and selected AML-relevant genes in OCI-AML5 cells treated with 1000 nM of OTX015 for 8 h.

Utilizing a previously validated, GFP-tagged gRNA library (1390 gRNAs and ~8 gRNA per gene) (Table S1) targeting chromatin regulators transduced into OCI-AML5 cells that stably expressed Cas9, we next conducted a protein domain-focused CRISPR-Cas9 screen to nominate potential drug targets in INCB-treated and untreated OCI-AML5 cells^41^. Eight days after transduction of gRNAs, cells were treated or untreated with INCB (250 nM) for 4 days. NGS was performed to determine negative selection of the gRNAs identified on day 12 in INCB-treated versus untreated OCI-AML5 cells, compared to the gRNA profile sequenced on day-2 following transduction of gRNAs^41^. Fig. S10 demonstrates the decline in % of GFP-positive cells noted on day-12 compared to day-2, with the fold sequencing coverage of guide RNAs maintained above ~400X (data not shown). Fig. 4A demonstrates the loss in gRNA reads targeting specific genes, including LSD1, BRD4, DOT1L, HDAC3, and MOZ. Based on this, we next determined the lethal effect of doxycycline-inducible BRD4 shRNA-mediated BRD4 KD in AML cells that were co-treated with INCB. Figure 4B, C demonstrate that BRD4 KD significantly increased INCB-induced CD11b and apoptosis of OCI-AML5 cells. As previously reported, treatment with the BETi OTX015 dose-dependently induced apoptosis in OCI-AML5 cells expressing LSD1-FKBP12^F36V^, which was significantly enhanced by LSD1 degradation following dTAG-13 treatment (Fig. 4D)^10^. In this setting, the effect of co-treatment with dTAG-13 on differentiation was not discernable. OTX015-induced apoptosis was also significantly enhanced by CRISPR-Cas9-mediated LSD1-KO in the post-MPN sAML SET-2 cells (Fig. 4E). ChIP-seq analysis demonstrated that, compared to control, OTX015 treatment caused downregulation of BRD4 occupancy at enhancers/promoters of genes, including MYC, CDK6, BCL2, MYB, PU.1, RUNX1, TCF7L2, and GFI1 (Fig. 4F). Consistent with this, OTX015 treatment depleted mRNA expressions of these genes, but induced mRNA levels of p21, ITGAM, LYZ, and LY96 (Fig. S11A, and data not shown). Exposure to OTX015 also repressed LSD1, GFI1, c-Myc, c-Myb, and PU.1, while inducing p21 and p27 protein levels in OCI-AML5 and SET-2 cells (Fig. S11B, D, E). OTX015 represses c-Myc and as a result represses LSD1––a target of c-Myc^42^. Co-treatment with INCB enhanced effects of OTX015 on specific mRNAs, decreasing CDK6 and MYC but increasing LY96, LYZ, ITGAM, and p21, as well as augmented effects on protein-expressions, reducing c-Myc, c-Myb, GFI1, and PU.1 but increasing levels of p21 and p27 in OCI-AML5 and SET-2 cells (Fig. S11A, D, E).

### Co-inhibition of GFI1/LSD1 and BRD4, HDAC3, MOZ, or DOT1L induces synergistic lethality in AML and post-MPN sAML cells

We next determined in vitro lethal effects of co-inhibition of LSD1 and the codependencies discovered by the CRISPR-Cas9 screen. Co-treatment with INCB and OTX015 or ABBV-075, a potent BETi^43^, synergistically induced apoptosis in the indicated AML and sAML cell lines with diverse genetic alterations documented by NGS (Figs. S11C, S12A, B). Differentiation due to the combination was not observed (data not shown). Similarly, ORY1001 and OTX015 treatment also exerted synergistic lethality (Fig. S11F). Whereas modestly effective alone, co-treatment with the HDAC3 inhibitor RGFP966^44^ (≥2.5 µM) and LSD1 depletion by dTAG-13 induced more differentiation, not apoptosis, than either drug-treatment alone (Fig. S13A and data not shown). Combined treatment with INCB and RGFP966 induced synergistic lethality in sAML SET2 while enhancing differentiation in OCI-AML5 cells (Bliss synergy scores >5) (Fig. S13B–E). Treatment with the MOZ inhibitor WM1119 (100–1000 nM) alone was also only modestly effective (Fig. S14A)^45^, but co-treatment with dTAG-13 induced significantly more differentiation (Fig. S14A) (*p* < 0.05). Co-treatment with INCB and WM1119 exerted similar effects as INCB and RGFP966 in the AML and sAML cells (Bliss synergy score >5.0 or CI values <1.0) (Fig. S14B–F). Consistent with the CRISPR screen result that DOT1L HMTase is a codependency with INCB treatment, co-treatment with INCB and the DOT1L inhibitor EPZ5676 synergistically induced lethality in AML and sAML cells (Fig. S15A)^46^. As also recently reported, we discovered that co-treatment with an LSD1i (INCB or ORY1001) and a DNA hypomethylating agent (decitabine) synergistically induced loss of viability in AML cells (Fig. S15B, C)^25^.

**Fig. 5:**
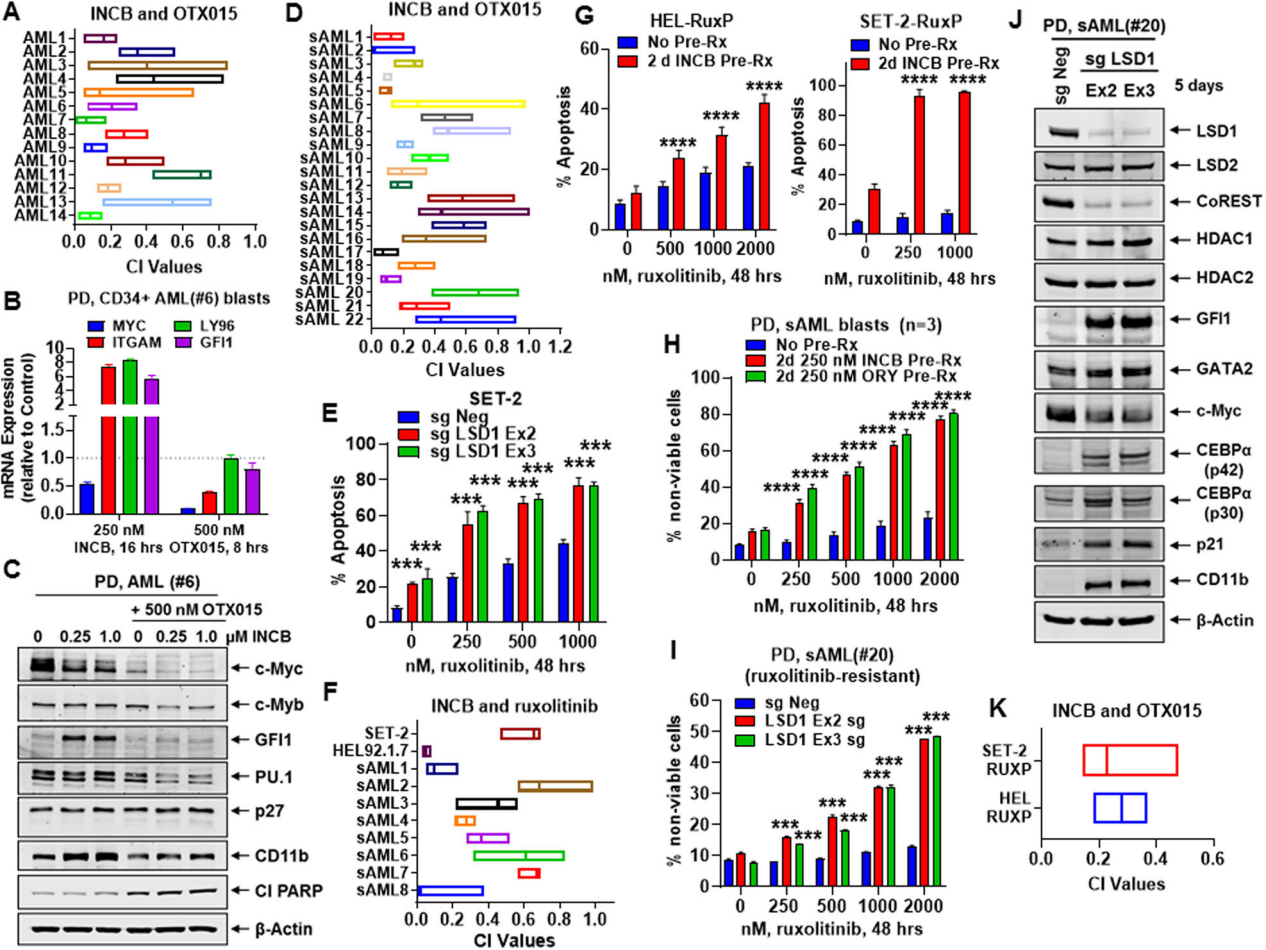
Co-treatment with LSD1i and BET inhibitor (BETi) or ruxolitinib exerts synergistic lethal activity in patient-derived AML and sAML cells. **A** PD CD34+ AML blast cells (*n* = 14) were treated with INCB059872 (100–1000 nM) and/or OTX015 (dose range 250–1000 nM) for 48 h. The % of To-Pro-3 iodide-positive, nonviable cells was determined by flow cytometry. Combination index values were calculated with CompuSyn. Combination index values <1.0 indicate a synergistic interaction of the drugs in the combination. **B** PD, AML #6 blast cells were treated (in triplicate) with INCB059872 or OTX015 as indicated. Total RNA was isolated, reverse transcribed and qPCR analysis was performed with TaqMan probes. The relative mRNA expression of each target was normalized to GAPDH and compared to the untreated control. **C** PD AML #6 cells were treated with the indicated concentrations of INCB059872 and/or OTX015 for 24 h. Total cell lysates were harvested and immunoblot analyses were conducted. The expression levels of β-Actin in the lysates served as the loading control. **D** PD CD34+ post-MPN, sAML blast cells (*n* = 22) were treated with INCB059872 (dose range: 100–1000 nM) and/or OTX015 (dose range 250–1000 nM) for 48 h. The % of To-Pro-3 iodide-positive, nonviable cells was determined by flow cytometry. Combination index values were calculated with CompuSyn. Combination index values <1.0 indicate a synergistic interaction of the drugs in the combination. **E** SET-2 cells transfected with sgNeg or LSD1 sgRNAs for 5 days were treated with the indicated concentrations of ruxolitinib for 48 h. Then, the % of annexin V-positive, To-Pro-3 iodide-positive, apoptotic cells was determined by flow cytometry. Mean of two independent experiments performed in duplicate + S.D. ***p < 0.005 compared to sgNeg-transfected cells treated with ruxolitinib (determined by a two-tailed, unpaired *t*-test). **F** SET-2, HEL92.1.7, and PD CD34+ post-MPN/MF sAML blast cells (*n* = 8) were treated with INCB059872 (100–1000 nM) and/or ruxolitinib (dose range 250–1000 nM) for 48 h. The % of annexin V-positive, apoptotic or To-Pro-3 iodide-positive, nonviable cells was determined by flow cytometry. Combination index values were calculated with CompuSyn. Combination index values < 1.0 indicate a synergistic interaction of the drugs in the combination. **G** HEL-RuxP and SET-2-RuxP cells were treated with the indicated concentrations of ruxolitinib with or without 2 days of pre-treatment with 250 nM of INCB059872. The % of annexin V-positive, apoptotic cells was determined by flow cytometry. Mean of three experiments + S.E.M. ****p < 0.001 compared to cells without INCB059872 pre-treatment (determined by a two-tailed, unpaired *t*-test). **H** PD, sAML blasts (*n* = 3) were treated with the indicated concentrations of ruxolitinib with or without 2 days of pre-treatment with 250 nM of INCB059872 or 250 nM of ORY1001. Then, the % of To-Pro-3 iodide-positive, nonviable cells was determined by flow cytometry. ****p < 0.001 compared to cells without LSD1i pre-treatment (determined by a two-tailed, unpaired *t*-test). **I** PD, sAML (#20) cells transfected (in duplicate) with sgNeg or LSD1 sgRNAs and incubated for 5 days were treated with the indicated concentrations of ruxolitinib for 48 h. The % of To-Pro-3 iodide-positive, nonviable cells was determined by flow cytometry. ***p < 0.005 compared to sgNeg-transfected cells treated with ruxolitinib (determined by a two-tailed, unpaired *t*-test). **J** Immunoblot analysis of PD, sAML (#20) 5 days posttransfection with sgNeg or LSD1 sgRNAs. **K** SET-2-RuxP and HEL-RuxP cells were treated with INCB059872 (100–1000 nM) and/or OTX015 (dose range 250–1000 nM) for 48 h. The % of annexin V-positive, To-Pro-3 iodide-positive, apoptotic cells was determined by flow cytometry. Combination index values were calculated with CompuSyn. Combination index values <1.0 indicate a synergistic interaction of the drugs in the combination.

Next, we evaluated the lethal activity of LSD1i and BETi in patient-derived (PD) AML blasts with genetic alterations documented by NGS (Fig. S12C). We determined that co-treatment with INCB and OTX015 for 48 h induced synergistic loss of viability in 14 samples of AML blasts (Fig. 5A). Exposure to INCB (250 nM for 16 h) reduced mRNA and protein levels of c-Myc, while inducing levels of GFI1, PU.1, ITGAM, (and LY96 mRNA) in PD AML blasts (Fig. 5B, C). In contrast, treatment with OTX015 did not induce mRNA or protein levels encoded by these genes, but attenuated MYC expression in the PD AML blasts (Fig. 5B, C). Co-treatment with INCB and OTX015 induced synergistic loss of viability in 22 samples of PD, post-MPN sAML blasts (combination index values < 1.0) (Figs. 5D and S12D). We also determined the effect of ruxolitinib in sAML SET-2 cells with CRISPR-Cas9 mediated KO of LSD1. As shown in Fig. 5E, ruxolitinib-induced apoptosis was significantly augmented in SET-2 cells with LSD1-KO (*p* < 0.005). Co-treatment with INCB and ruxolitinib also synergistically induced lethality in HEL92.1.7 and SET-2, as well as in samples of PD post-MPN sAML blasts in which genetic mutations were documented by NGS (CI values < 1.0) (Figs. 5F and S12D). Notably, in previously described nongenetic ruxolitinib-persister/resistant HEL92.1.7-RuxP and SET-2-RuxP cells, unlike in parental SET-2 or HEL92.1.7 cells, pre-treatment with INCB for 48 h, markedly enhanced ruxolitinib-induced apoptosis in HEL-RuxP and SET-2-RuxP cells (*p* < 0.001) (Fig. 5G)^47^. Pre-treatment with INCB or ORY1001 for 48 h, sensitized ruxolitinib-resistant PD CD34+ blast progenitor cells (3 samples) to ruxolitinib-induced loss of cell viability (Fig. 5H). Additionally, as shown in Fig. 5I, in a short-term in vitro culture of PD, post-MPN sAML progenitor cells (sAML#20), LSD1-KO via CRISPR-Cas9 significantly enhanced ruxolitinib-induced lethality (*p* < 0.01). In sAML#20, CRISPR-Cas9-mediated LSD1-KO depleted protein levels of LSD1, CoREST and c-Myc, but increased GFI1, GATA2, CEBPα, p21, and CD11b, without altering protein levels of HDACs1/2 (Fig. 5J). These studies suggest that co-treatment with LSD1i and ruxolitinib might overcome development of nongenetic ruxolitinib resistance in post-MPN sAML cells^48^. Co-treatment with INCB and the BETi OTX015 also exerted synergistic lethality in HEL-RuxP and SET-2-RuxP cells (CI values <1.0) (Fig. 5K).

### Pretreatment with LSD1i overcomes nongenetic BETi-resistance in AML and post-MPN sAML blasts

**Fig. 6:**
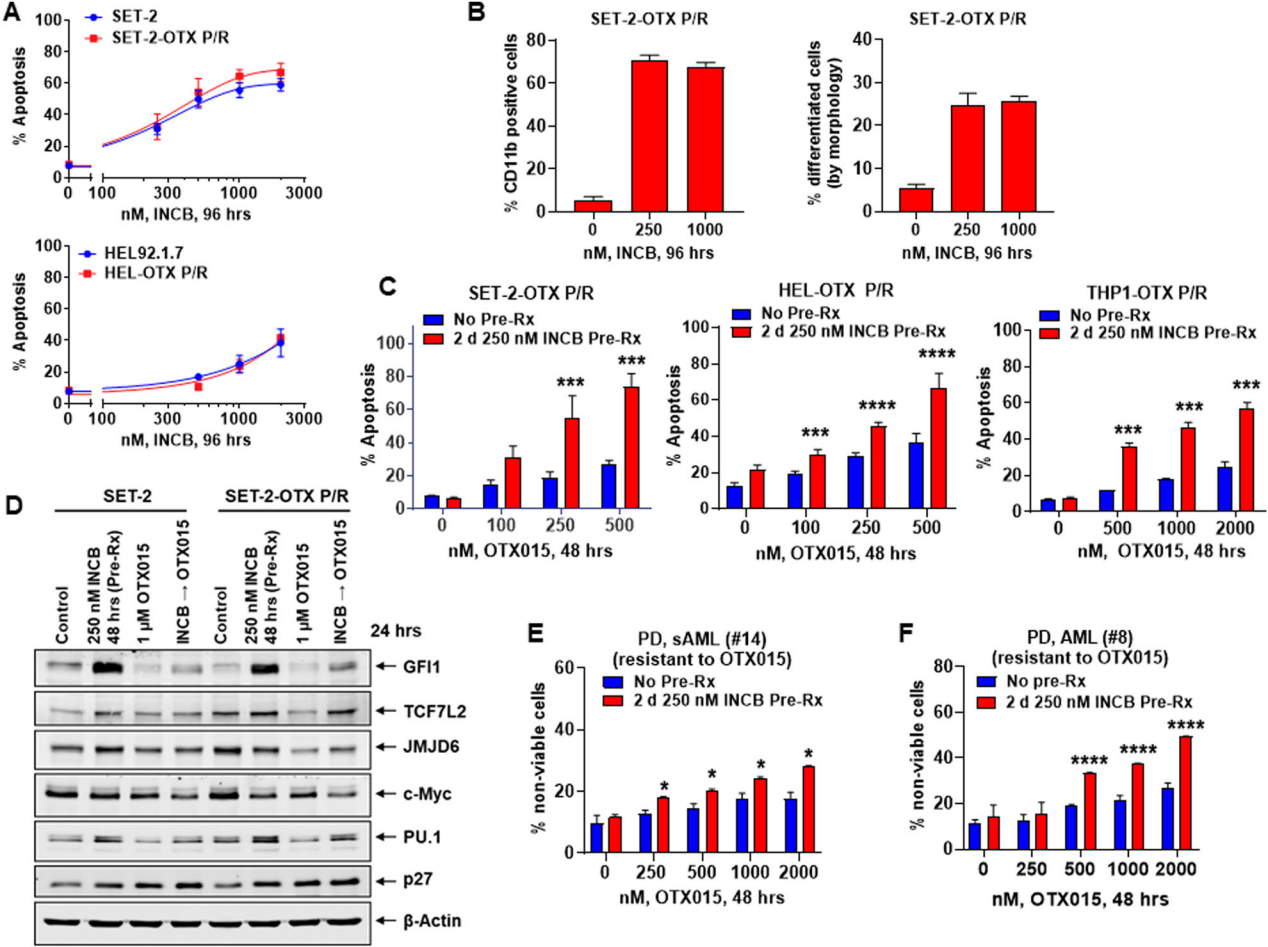
LSD1i pre-treatment sensitizes BETi persister/resistant post-MPN sAML and de novo AML cells to BETi. **A** HEL92.1.7, HEL-OTX P/R, SET-2 and SET-2-OTX P/R cells were treated with INCB059872 for 96 h. The % of annexin V-positive, To-Pro-3 iodide-positive, apoptotic cells was determined by flow cytometry. Mean of three experiments + S.D. **B** SET-2-OTX P/R cells were treated with the indicated concentrations of INCB059872 for 96 h. Differentiated cells were assessed by CD11b flow cytometry and by cell morphology. Mean of three experiments + S.D. **C** SET-2-OTX P/R, HEL-OTX P/R, and THP1-OTX P/R cells were treated with the indicated concentrations of OTX015 with or without 2 days of pre-treatment with 250 nM of INCB059872. The % of annexin V-positive, apoptotic cells was determined by flow cytometry. Mean of three experiments + S.D. ***p < 0.005, ****p < 0.001 compared to cells treated with OTX015 without INCB059872 pre-treatment (determined by a two-tailed, unpaired *t*-test). **D** Immunoblot analyses conducted on total cell lysates from SET-2 and SET-2-OTX P/R cells pre-treated with INCB059872 (48 h) and/or OTX015 (24 h), as indicated. **E** PD, sAML (#14) cells (ex vivo resistant to OTX015) were treated (in duplicate) with the indicated concentrations of OTX015 with or without 2 days of pre-treatment with 250 nM of INCB059872. The % of To-Pro-3 iodide-positive, nonviable cells was determined by flow cytometry. *p < 0.05 compared to cells treated with OTX015 without INCB059872 pre-treatment (determined by a two-tailed, unpaired *t*-test). **F** PD, AML (#8) blast cells (ex vivo resistant to OTX015) were treated (in duplicate) with the indicated concentrations of OTX015 with or without 2 days of pre-treatment with 250 nM of INCB059872. The % of To-Pro-3 iodide-positive, nonviable cells was determined by flow cytometry. ****p < 0.001 versus cells treated with OTX015 without INCB059872 pretreatment (determined by a two-tailed, unpaired *t*-test).

We recently described BETi-persister/resistant (BETi-P/R) post-MPN sAML and AML cells (SET-2-OTX P/R, HEL-OTX P/R, and THP1-OTX P/R cells) that displayed nongenetic mechanism of BETi-resistance, based on increased expression and activity of β-catenin-TCF7L2-JMJD6-c-Myc (Fig. S16A)^33^. Exposure to INCB for 96 h dose-dependently induced differentiation and apoptosis to a similar level in SET-2 and SET-2-OTX P/R as well as HEL92.1.7 and HEL-OTX P/R cells, although exposure to INCB for 48 h was relatively inactive in inducing apoptosis (Figs. 6A, B, and S16B). However, pretreatment with INCB for 48 h, significantly increased BETi-induced apoptosis in SET-2-OTX P/R, HEL-OTX P/R and THP1-OTX P/R cells (Fig. 6C). Pretreatment with ORY-1001 had a similar effect in sensitizing BETi-P/R cells to BETi-induced apoptosis (Fig. S16C, D). In SET-2-OTX P/R cells, qPCR analyses demonstrated that, compared to treatment with INCB alone, pretreatment with INCB followed by OTX015 caused inhibition of mRNA expressions GFI1, TCF7L2, JMJD6, MYC, ITGAM, LY96, BCL2, and BCL2L1 but increased p21 mRNA levels (Fig. S16E). Western analyses showed that pretreatment with INCB followed by OTX015 caused abrogation of GFI1, TCF7L2, and PU.1 induced by INCB treatment alone, with significant decline in c-Myc and JMJD6 but increase in p27 levels (Fig. 6D). A significant increase in OTX015-induced lethality was also observed following pretreatment with INCB of PD AML and sAML blasts (Fig. 6E, F). Similar to a recent report, we also determined the effects of INCB pretreatment on chromatin accessibility and potential enhancer reprogramming that could explain sensitization to BETi^49^, by conducting ATAC-Seq and RNA-Seq analyses in INCB-treated versus -untreated SET-2 OTX P/R cells. INCB treatment caused increases in ATAC-Seq peaks in the chromatin of GFI1, PU.1, and IRF8 target genes (Fig. S17A–C). Consistent with this, RNA-Seq analysis also showed increases in mRNA expressions of GFI1, PU.1, and CEBPα target genes, with concomitant decline in mRNAs of several MYC-target genes (Fig. S17D–G). These findings highlight that INCB pretreatment potentially commissions enhancers of GFI1, PU.1, IRF8, and MYC genes and their targets to sensitize BETi-P/R sAML cells to BETi-induced lethality.

### In vivo efficacy of combination of LSD1i with ruxolitinib or BETi against post-MPN sAML cells

**Fig. 7:**
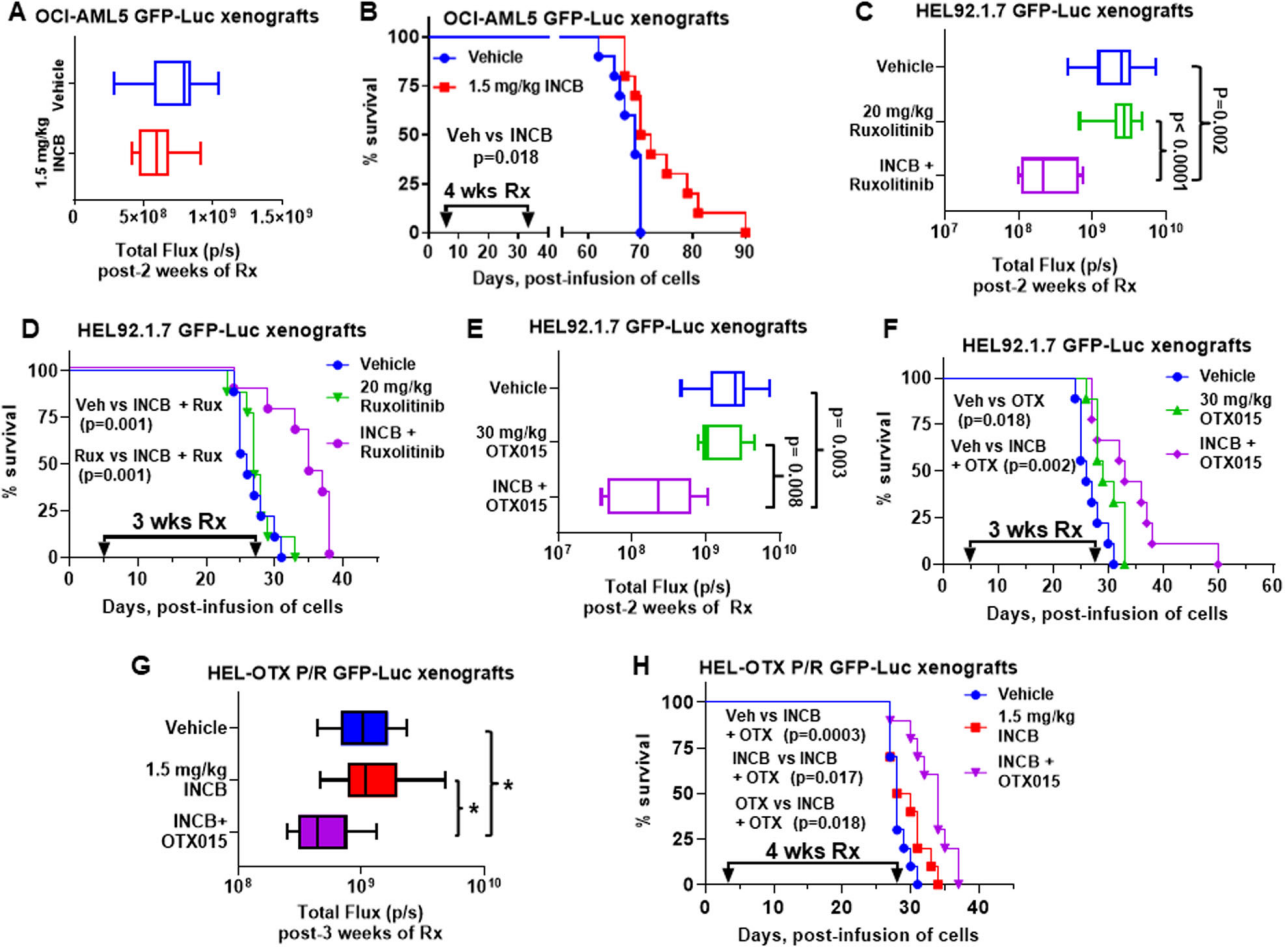
Treatment with INCB059872 and ruxolitinib or OTX015 reduced leukemia burden and improved survival of NSG mice engrafted with sAML xenografts. **A** Total photon counts [flux] (determined by bioluminescent imaging) in NSG mice engrafted with OCI-AML5 GFP-Luc cells and treated for 2 weeks with INCB059872. **B** Kaplan–Meier survival plot of NSG mice engrafted with OCI-AML5 GFP-Luc cells and treated with 1.5 mg/kg of INCB059872 (daily x 5 days, P.O.) for 4 weeks. Significance was calculated by a Mantel–Cox log-rank test. **C** Total photon counts [flux] in NSG mice engrafted with HEL92.1.7 GFP-Luc cells and treated for 2 weeks with INCB059872 and/or ruxolitinib. **D** Kaplan–Meier survival plot of NSG mice engrafted with HEL92.1.7 GFP-Luc cells and treated with 1.5 mg/kg of INCB059872 (daily x 5 days, P.O.) and/or 20 mg/kg of ruxolitinib (daily x 5 days, P.O.) for 3 weeks. Significance calculated by Mantel–Cox log-rank test. **E** Total photon counts [flux] in NSG mice engrafted with HEL92.1.7 GFP-Luc cells and treated for 2 weeks with INCB059872 and/or OTX015. **F** Kaplan–Meier survival plot of NSG mice engrafted with HEL92.1.7 GFP-Luc cells and treated with 1.5 mg/kg of INCB059872 (daily x 5 days, P.O.) and/or 30 mg/kg of OTX015 (daily x 5 days, P.O.) for 3 weeks. Significance calculated by Mantel–Cox log-rank test. **G** Total photon counts [flux] in NSG mice engrafted with HEL-OTX P/R GFP-Luc cells and treated for 1 week with INCB059872 followed by two weeks of INCB059872 and/or OTX015. *p < 0.05 (determined by a two-tailed, unpaired *t*-test). **H** Kaplan–Meier survival plot of NSG mice engrafted with HEL-OTX P/R GFP-Luc cells and treated with 1.5 mg/kg of INCB059872 (daily x 5 days, P.O.) alone for 1 week, followed by 3 weeks of 1.5 mg/kg of INCB059872 (daily x 5 days, P.O.) and/or 50 mg/kg OTX015 (daily x 5 days, P.O.). Significance was calculated by a Mantel–Cox log-rank test.

Given the marked in vitro synergy noted above, we determined in vivo antileukemia efficacy of INCB alone or co-treatment with ruxolitinib or OTX015 in NSG mice engrafted with AML or post-MPN sAML cells. Treatment with INCB alone for 3–4 weeks reduced AML and sAML burden and significantly improved overall survival of NSG mice engrafted with OCI-AML5 or HEL92.1.7 cells, respectively (Figs. 7A, B, S18A, B). Additionally, compared to ruxolitinib alone, which did not favorably affect sAML burden or the NSG mice survival, co-treatment with INCB and ruxolitinib significantly reduced sAML burden and improved overall survival of the NSG mice engrafted with HEL92.1.7 cell (Fig. 7C, D). Next, we determined effect of co-treatment with LSD1i on in vivo efficacy of BETi in NSG mice engrafted with BETi-sensitive or BETi-resistant sAML cells. Compared to monotherapy with OTX015, combined therapy with INCB and OTX015 for 3 weeks significantly reduced sAML burden and improved survival of NSG mice engrafted with BETi-sensitive HEL92.1.7 cells without causing weight loss or other signs of host toxicity (Fig. 7E, F, and data not shown). Notably, co-treatment with INCB and OTX015 also significantly reduced sAML burden and improved overall survival of NSG mice engrafted with BETi-resistant HEL-OTX P/R cells, again without resulting in any weight loss or host toxicity (Fig. 7G, H and data not shown). These findings suggest that combined therapy with LSD1i and BETi may exert in vivo efficacy and overcome nongenetic BETi-P/R in post-MPN sAML cells.

## Discussion

Utilizing the dTAG system to degrade LSD1 and CRISPR-Cas9 to edit and KO LSD1, findings presented here highlight gene-expression perturbations that lead to differentiation of AML and post-MPN sAML blasts. Also, disruption of LSD1 interactions with CoREST-HDAC1/2 complex and with GFI1/1B by LSD1i activates GFI1/1B-repressed enhancers and transcription factors, including PU.1, CEBPα and IRF8 while repressing c-Myc, and their target gene-expressions. This induces cell cycle inhibitors including p21 and p27, up-regulates differentiation-associated gene-expressions such as CD11b, CD86, LY96, and LYZ, and induces morphologic features of AML cell differentiation. Although LSD1i inhibits both the scaffold and enzymatic functions of LSD1, AML cell-differentiation occurs independent of inhibiting enzymatic demethylase function of LSD1^17,23,25,26,50^. CRISPR-suppressor scanning was utilized to show that LSD1 enzyme activity is not required for AML survival^26^. Instead, LSD1 inhibitors disrupt interaction between LSD1 and transcription repressor GFI1/1B on the chromatin. Also, in a knockdown and rescue experiment, the LSD1 K661A catalytic mutant was as capable of rescuing the clonogenic potential of LSD1 knockdown cells as the wild type LSD1 protein^23,50^. Additionally, LSD1 inhibitor treatment induces rapid and extensive transcriptional alterations, which do not correlate with LSD1i-mediated changes in H3K4 mono- and dimethyl-chromatin marks^23,50^.

Additional findings highlighted here are that disruption of LSD1-CoREST-HDAC1/2 complex derepresses GFI1/1B SE/Es to cause early induction of GFI1/1B^47^, which could serve as a predictive early biomarker of achieving biologically effective intracellular levels of LSD1i in AML and post-MPN sAML cells. LSD1-KO also increased H3K27Ac and BRD4 occupancy at the GFI1 locus inducing GFI1 in AML cells. However, it is noteworthy that without intact LSD1-CoREST-HDAC1/2 complex, upregulated GFI1/1B is unable to repress differentiation in AML/sAML cells^19,21^. Studies presented here also involved CRISPR-Cas9-mediated KO of LSD1 or shRNA-mediated KD of LSD1, which showed induction of GFI1/1B, as well as of PU.1, CEBPα, and p21, but repression of c-Myc in both post-MPN-sAML and non-MPN associated AML cells. Notably, reduced H3K27Ac occupancy at the MYC, but increased at the ITGAM, LY96, and LYZ loci, induced ITGAM, LY96, and LYZ expressions, differentiation and cell lethality of AML and post-MPN sAML blasts. Our findings also confirmed that CRISPR-Cas9-mediated GFI1-KO or shRNA-mediated GFI1-KD also induced differentiation markers and morphologic features of differentiation in AML cells, albeit to a lesser extent than knockout or knockdown of LSD1. However, these findings also demonstrated that LSD1i treatment augmented differentiation due to GFI1-KD. This suggests that disruption of LSD1 binding to repressive complexes not involving GFI1/1B may also be contributing to LSD1i-induced differentiation in AML cells^17,18,51^. Alternatively, it also suggests that both scaffold and enzymatic functions of LSD1 (inhibited by INCB) may be involved in AML cell differentiation induced by INCB^38^. Again, LSD1i-induced differentiation was also associated with induction of GFI1, PU.1, and p21, but depletion of c-Myc in both post-MPN-sAML and non-MPN associated AML cells. This highlights targeted degradation and depletion of LSD1, which would disrupt both scaffolding and enzymatic activities of LSD1, as an attractive therapeutic approach. Notably, LSD1-KO decreased H3K27Ac occupancy at enhancers/promoters and mRNA expressions of MYC and WNT-β-catenin-target genes, associated with positive enrichment of HALLMARK gene-sets of innate immunity, inflammatory response and apoptosis in AML cells. LSD1-KD by shRNA also positively enriched similar HALLMARK gene-sets in AML cells. These findings are consistent with recent reports that LSD1i treatment stimulates antitumor immunity by inhibiting Foxp3+ Treg cell function, thus enabling and enhancing immune checkpoint blockade^52,53^. Collectively, these findings highlight testing potential utility of including LSD1i co-treatment with immune therapies in AML.

Utilizing a protein domain-focused CRISPR-Cas9 screen, our studies revealed that BRD4, DOT1L, HDAC3 and MOZ could serve as effective co-targets to achieve synergistic efficacy with LSD1i against AML and sAML cells. Consistent with this, LSD1i and BETi co-treatment exhibited synergistic loss of viability in PD AML and post-MPN sAML blasts displaying diverse genetic alterations. In addition to abrogating LSD1i-induced GFI1 and PU.1, BRD4 inhibition by BETi increased lethal activity of LSD1i most likely due to observed greater decline in c-Myc and increased p21 and p27 protein levels. Additionally, in previous reports, we have highlighted that inclusion of BETi enhances lethal activity of BETi-based combinations due to greater depletion of c-Myc, cell cycle dependent kinases CDK4 and CDK6, as well as concomitant induction of CDK inhibitor p21, pro-apoptotic (BIM) and downregulation of anti-apoptotic (MCL1, BCL-xL, and BCL2) proteins.^33,43,54–56^. Synergistic in vitro lethality was also observed due to co-treatment with LSD1i and agents targeting HDAC3, MOZ or DOT1L. However, whether these LSD1i-based combinations would also exert superior in vivo anti-AML or anti-sAML efficacy remains to be determined. Notably, a dual class I HDACi and LSD1 inhibitor was shown to exert synergistic lethality in AML as well as in tumor models other than AML^30,57,58^. CRISPR-Cas9 screens in AML cells expressing MLL-fusion gene showed superior efficacy of co-targeting mTORC1 with LSD1, which was not revealed by our screen^59^. This difference is because our domain-focused CRISPR-Cas9 screen involved AML cells without MLL fusion gene-expression, and utilized gRNAs targeting chromatin regulators not mTORC1. Our findings also demonstrate that co-treatment with LSD1i and the JAK1/2 inhibitor ruxolitinib exerts synergistic in vitro lethality and exhibited superior in vivo efficacy than LSD1i monotherapy in a mouse xenograft model of post-MPN sAML. A recent report highlighted that LSD1i treatment exhibited clinical efficacy by reducing spleen size and constitutional symptoms in patients with advanced MPN, thus combination therapy with LSD1i and JAKi merits further in vivo evaluation^60^. Our studies demonstrating synergistic in vitro lethality due to combination of LSD1i with decitabine also merits further verification through in vivo AML xenograft studies.

There is a growing evidence and recognition of epigenetic heterogeneity and plasticity in cancer cells, which under selection pressure of targeted therapies facilitates emergence of drug-tolerant persister/resistant cancer cells displaying nongenetic therapy-resistance^61–63^. Agents that target signaling kinases (e.g., JAK1/2) and epigenetic mechanisms (e.g., BET proteins) were shown to result in enhancer reprogramming via newly-marshaled activities of lineage-specific transcriptional regulators which creates the transcriptome/proteome conferring drug tolerant persister/resistance in AML cells^33,48,49^. We previously reported that increased c-Myc expression due to increased levels and activity of β-catenin-TCF7L2-JMJD6-MYC axis induced nongenetic BETi-persister/resistance in AML and sAML blasts^33^. This was due to enhancer reprogramming and increased transcriptional activities of MYC, RUNX1, and TCF7L2, based on H3K27Ac and BRD4 occupancy at their Es/promoters^33^. Our findings demonstrate, for the first time, that LSD1i is as active in inducing differentiation and lethality in the BETi-P/R as in BETi-sensitive sAML cells. Notably, sensitization of BETi-P/R sAML and AML cells to BETi-induced apoptosis by pre-treatment with LSD1i was associated with increased chromatin accessibility and mRNA expressions of GFI1, PU.1, and IRF8-target genes. However, mRNA expressions of c-Myc targets were mostly downregulated. Importantly, our findings also show that LSD1i pre-treatment similarly sensitized sAML-RuxP cells that display nongenetic resistance to ruxolitinib^48^. Taken together, these findings highlight that, by reprogramming of SEs/Es and modifying gene-expressions involved in conferring nongenetic persister/resistance, LSD1i treatment could sensitize AML to BETi, and sAML cells to ruxolitinib or BETi, thereby highlighting novel differentiation and lethal therapies for AML or post-MPN sAML. Findings presented here also beg the question where in AML or post-MPN sAML therapy LSD1i-based combinations should best be clinically tested for their efficacy, i.e., during induction therapy or during consolidation therapy to effectively eliminate the MRD. Due to their demonstrable ability to induce differentiation and eliminate AML blast progenitors, as well as in augmenting immune checkpoint blockade therapy, LSD1i-based combinations should be likely best tested for efficacy in patients with AML in CR but detectable MRD that eventually relapses as therapy-refractory AML.

## Materials and methods

### Cell lines and cell culture

OCI-AML5, OCI-AML2, Mono-Mac-1, MOLM13, SKM-1, and SET-2 cells cells were obtained from the DSMZ. MV4-11, HEL92.1.7, THP1, and HS5 cells were obtained from the ATCC (Manassas, VA). HEK-293T cells were obtained from the Characterized Cell Line Core (CCLC) at M.D. Anderson Cancer Center, Houston TX. Experiments with cell lines were performed within 6 months after thawing or obtaining from ATCC or DSMZ. Cell lines were authenticated in the CCLC at M.D. Anderson Cancer Center. Logarithmically growing, mycoplasma-negative cells were utilized for all experiments.

### Assessment of apoptosis by annexin-V staining

Untreated or drug-treated cells were stained with Annexin-V (Pharmingen, San Diego, CA) and TO-PRO-3 iodide (Life Technologies, Carlsbad, CA) and the % of apoptotic cells were determined by flow cytometry. To analyze synergism, cells were treated with combinations for 48 h and the % of apoptotic or nonviable cells were determined by flow cytometry. The combination index (CI) for each drug combination was calculated utilizing CompuSyn software. We also utilized matrix dosing of agents in combinations to allow synergy assessment by Bliss scores utilizing the SynergyFinder online web application tool.

### RNA isolation and quantitative polymerase chain reaction

Following the designated treatments, total RNA was isolated from AML or sAML cells utilizing a PureLink RNA Mini kit from Ambion, Inc. (Austin, TX) and reverse transcribed. Quantitative real time PCR analysis was performed on cDNA using TaqMan probes. Relative mRNA expression was normalized to GAPDH and compared to the untreated cells.

### Statistical analysis

Significant differences between AML or sAML cells treated with different experimental conditions compared to control cells were determined using the two-tailed, unpaired *t*-test. For the in vivo mouse models, a two-tailed, unpaired *t*-test was utilized for comparing total bioluminescent flux. For survival analysis, a Kaplan–Meier plot and a Mantel–Cox log rank test were utilized for comparisons of different cohorts. *P* values of <0.05 were assigned significance.

## Supplementary information

Reproducibility Checklist

Supplemental Figure legends

Supplemental Figures

Supplemental Materials and Methods

Table S1. Guide RNA targets and sequences

## Data Availability

RNA-Seq, ChIP-Seq, and ATAC-Seq datasets have been deposited in GEO as a super series under accession ID GSE160319. Detailed Methods for conducting ATAC-Seq, ChIP-Seq, and RNA-Seq in AML cells, assessment of leukemia cell differentiation, CRISPR-Cas9-mediated gene editing, domain-scanning CRISPR dropout screen as well as the in vivo models in de novo AML and post-MPN sAML cells are provided in the Supplementary Methods.
