# Supplementary material for: Superior efficacy of co-targeting GFI1/KDM1A and BRD4 against AML and post-MPN secondary AML cells
Source: Blood Cancer J. 2021 May 20;11(5):98. doi: 10.1038/s41408-021-00487-3 (PMC8138012; doi:10.1038/s41408-021-00487-3)
Supplement: Supplementary file 2 — Supplemental Figure legends [file 41408_2021_487_MOESM2_ESM.docx]

**Supplemental Figure Legends**

**Supplemental Figure 1. Knockout of LSD1 by CRISPR/Cas9 or degradation by dTAG-13 induces cell cycle G0/G1 accumulation, decreases cell proliferation and derepresses myeloid differentiation gene CD11b in AML and sAML cells. A.** Cell cycle distribution of OCI-AML5/Cas9 cells transduced with LSD1 sgRNAs and incubated for 8 days. Mean of two independent experiments performed in duplicate + S.D. **B**. Representative immunoblot analysis of SET2 cells transfected with two sgRNAs against LSD1 and incubated for 7 days. **C**. Cell proliferation of SET-2 cells transfected with LSD1 sgRNAs for 5 days. Following this, cells were plated at 0.125 million/mL and cultured for 96 hrs. Mean of two independent experiments + S.D. *** = p<0.005 compared to sgNeg-transfected SET-2 cells (determined by a two-tailed, unpaired t-test). **D**. Expression of c-Kit and CD11b (assessed by flow cytometry) in SET-2 cells with LSD1 knockout, 7 days post-transfection. **E**. Representative immunoblot analysis of OCI-AML5/LSD1-FKBP12(^F36V^) cells treated with the indicated doses of dTAG-13 for 8 hours. **F**. Quantification of selected protein expressions in OCI-AML5/LSD1-FKBP12(^F36V^) cells following 8 hours of treatment with 500 nM of dTAG-13. Mean of two independent experiments + S.D. **G-H**. Immunoblot analysis of OCI-AML5/LSD1-FKBP12(^F36V^) cells treated with the indicated doses of dTAG-13 for 24 hours. **I**. OCI-AML5/LSD1-FKBP12(^F36V^) cells were treated with 500 nM of dTAG-13 for 96 hours. At the end of treatment, CD11b expression was assessed by flow cytometry. Mean of three experiments + S.E.M. **** = p<0.001 relative to untreated cells (determined by a two-tailed, unpaired t-test).

**Supplemental Figure 2. Knockout of LSD1 decreases H3K27Ac occupancy on WNT Beta Catenin and MYC target genes while increasing occupancy on myeloid differentiation genes and inducing mRNA expression of PU.1 target genes in AML cells.** **A**. IGV plots of sequence tag densities at the MYC, CDK6, LY96, LYZ, and ITGAM gene loci in OCI-AML5 LSD1 knockout cells compared to Cas9-only-expressing cells. Locations and log2 fold-changes in peak densities are denoted by blue bars. **B-C**. Log2 fold-change in H3K27Ac ChIP-Seq peaks on MYC target genes I and MYC target genes II (HALLMARK datasets) in LSD1 knockout compared to Cas9 only-expressing OCI-AML5 cells. **D**. IGV plot of sequence tag densities for H3K27Ac and BRD4 ChIP-Seq and super enhancer analysis at the GFI1 locus in OCI-AML5 LSD1 knockout and Cas9-only-expressing cells. Locations and log2 fold-changes in peak densities for H3K27Ac and BRD4 are denoted by blue bars. The super enhancer size and location was determined by ROSE analysis. **E-F**. Log2 fold-change in mRNA expression levels of PU.1 and CEBPα target genes in OCI-AML5 LSD1 knockout cells compared to Cas9-only-expressing cells. **G**. Gene set enrichment analysis of RNA expression changes in OCI-AML5 LSD1 knockout cells compared to HALLMARK pathways. The q-values are < 0.1 in all comparisons. **H**. Relative expression levels of enhancer RNAs within MYC enhancer peaks (E2-E5) were determined by qPCR in OCI-AML5 LSD1 knockout compared to Cas9-only-expressing cells. Mean of two independent experiments + S.D.

**Supplemental Figure 3. Exon structure and protein domains in KDM1A (LSD1) and GFI1. A. (upper)** Exon composition of KDM1A. Red boxes indicate coding exons. Black boxes indicate untranslated regions. (**Lower**) Protein and functional domains of KDM1A. The arrows indicate the position of the shRNAs developed against KDM1A and the domains of the protein they target. **B. (upper)** Exon composition of GFI1. Red boxes indicate coding exons. Black boxes indicate untranslated regions. (**Lower**) Protein and functional domains of GFI1. The arrows indicate the position of the shRNAs developed against GFI1 and the domains of the protein they target.

**Supplemental Figure 4. Depletion of LSD1 by shRNA upregulates myeloid differentiation genes CD11b and CD86 and induces apoptosis in AML cells. A**. OCI-AML5 cells were stably transduced with doxycycline-inducible shRNAs against LSD1 or a non-targeting shRNA. Relative mRNA expression was determined by qPCR analysis (of biologic triplicates) after 96 hours of doxycycline treatment. **B**. Representative immunoblot analysis in OCI-AML5 LSD1 knockdown cells following 96 hour of doxycycline treatment. **C**. Expression of CD86 and CD11b (assessed by flow cytometry) in OCI-AML5 LSD1 shRNA knockdown cells after 96 hours of doxycycline treatment. Mean of three experiments + S.D. **D**. Morphologic differentiation (% myelocytes, meta-myelocytes or bands) in OCI-AML5 LSD1 knockdown cells after 96 hours of doxycycline treatment. Mean of three experiments + S.D. **E**. Relative mRNA expression of LY96, ITGAM, and CD86 in OCI-AML5 GFI1 knockdown cells compared to shNT cells 96 hours post transduction. Expression was normalized to GAPDH. **F**. OCI-AML5 cells were transduced with shNT and GFI1 shRNA-expressing lentivirus and incubated for 96 hours. At the end of treatment, total cell lysates were prepared for immunoblot analysis. **G**. Heat map showing the number of mRNAs depleted or induced > 1.25 fold and p-value < 0.05 (assessed by RNA-Seq analysis in biologic triplicates) in OCI-AML5 LSD1 knockdown cells after 96 hours of doxycycline treatment. **H**. OCI-AML5 cells were transduced with shNT or GFI1 shRNA-expressing lentivirus and incubated for 96 hours. Total RNA was isolated, purified, and utilized for RNA-Seq analysis (biologic duplicates). The heat map shows the number of mRNAs induced or depleted > 1.25 fold and p-value < 0.05. **I**. Log2 fold-change in mRNA expression levels of PU.1 and GFI1 target genes in OCI-AML5 LSD1 knockdown cells compared to sh-NT-expressing cells. **J**. MOLM13 cells were stably transduced with doxycycline-inducible shRNAs against LSD1 or a non-targeting shRNA. (i). Relative mRNA expression (in biologic triplicates) was determined by qPCR analysis after 96 hours of doxycycline treatment. (ii). Representative immunoblot analysis of MOLM13 LSD1 knockdown cells following 96 hour of doxycycline treatment. (iii). Expression of CD86 and CD11b (assessed by flow cytometry) in MOLM13 sh-NT and LSD1 shRNA knockdown cells after 96 hours of doxycycline treatment. Mean of two independent experiments performed in triplicate + S.D. (iv). Morphologic differentiation (% myelocytes, meta-myelocytes or bands) in MOLM13 sh-NT and LSD1 knockdown cells after 96 hours of doxycycline treatment. Mean of three experiments + S.D.

**Supplemental Figure 5. Knockdown of GFI1 by shRNA induces GFI1 target genes and mRNA expressions that overlap with LSD1 knockdown in AML cells. A**. Gene set enrichment analysis of RNA expression changes (determined by RNA-Seq) in OCI-AML5 LSD1 knockdown (**top**) and GFI1 knockdown (**bottom**) cells compared to HALLMARK pathways. The q-values are < 0.1 in all comparisons. **B**. Venn diagram of up and downregulated genes in OCI-AML5 LSD1 and GFI1 shRNA-expressing cells as determined by RNA Seq analysis. **C**. Log2 fold-change of selected gene expression alterations (determined by RNA-Seq) common to LSD1 and GFI1 shRNA knockdown in OCI-AML5 cells. * indicates a PU.1 target gene.

**Supplemental Figure 6. Treatment with LSD1 inhibitor ORY1001 disrupts LSD1 binding to CoREST, inhibits colony growth and induces differentiation of AML cells. A-B**. Immunoprecipitation and immunoblot analysis of OCI-AML5 or THP1 cells treated with the indicated concentrations of ORY1001 for 16 hours. **C**. SET-2 cells were treated with the indicated concentrations of ORY1001 for 16 hours and immunoblot analysis was conducted. **D**. OCI-AML5 and MOLM13 cells were treated with ORY1001 for 96 hours then plated in methylcellulose and incubated at 37°C. Colony growth was assessed after 7-10 days. Mean of two independent experiments performed in duplicate + S.D. **E-F**. OCI-AML5, THP1 and MOLM13 cells were treated with ORY1001 for 96 hours. The % of CD86 and CD11b-positive cells were determined by flow cytometry. Mean of two independent experiments performed in duplicate + S.D.

**Supplemental Figure 7. Depletion of GFI1 increases sensitivity to LSD1 inhibitor-mediated induction of myeloid differentiation and cell death. A-B**. OCI-AML5 cells with GFI1 knockdown (by shRNA) were treated with the indicated concentrations of INCB059872 for 96 hours. At the end of treatment, the % of CD86 and CD11b-positive cells were determined by flow cytometry. Mean of two independent experiments performed in duplicate + S.D. *= p< 0.05; **= p< 0.01; ****= p< 0.001 (determined by two-tailed, unpaired t-test). **C**. OCI-AML5 cells with GFI1 knockdown were treated with the indicated concentrations of INCB059872 for 96 hours. At the end of treatment, cells were cytospun onto glass slides and differentiation was assessed by cell morphology. Mean of two independent experiments + S.D. **D**. OCI-AML5 cells were transduced with GFI1 shRNA and selected for 96 hours. Then, cells were treated with 250 nM of INCB059872 for 24 hours. Total cell lysates were prepared and immunoblot analyses were conducted. **E**. OCI-AML5 cells with GFI1 knockout were treated with the indicated concentrations of INCB059872 for 96 hours. The % of CD11b-positive cells was determined by flow cytometry. Mean of two independent experiments + S.D. *= p< 0.05; ***= p< 0.005 (determined by a two-tailed, unpaired t-test).

**Supplemental Figure 8. Treatment with INCB059872 alters chromatin accessibility as well as H3K27Ac and BRD4 occupancy in AML cells. A**. ATAC-Seq-determined gained and lost peaks in OCI-AML5 cells treated with 1000 nM of INCB059872 for 16 hours. **B**. Log2 odds-ratio of the location of ATAC-Seq peaks following treatment with LSD1 inhibitors segmented according to the major chromatin states defined by the NIH Roadmap Epigenomics Consortium. **C**. Motif analysis was conducted on the gained ATAC-Seq peaks in INCB059872-treated OCI-AML5 cells. A ranked order (based on –log10 p-value) of transcription factor motifs in the gained ATAC-Seq peaks is shown. **D**. Ranked order and consensus binding site of myeloid lineage transcription factor motifs in gained ATAC-Seq peaks following 16 hours of treatment with INCB059872 in OCI-AML5 cells. **E-F**. Log2 fold-change in ATAC-Seq peaks at TSS + 3 kb (promoters) or gene body + 10 kb (enhancers) of GFI1 and PU.1 target genes in OCI-AML5 cells treated with INCB059872 for 16 hours. **G**. ATAC-Seq-determined gained and lost peaks in SET2 cells treated with 250 nM of INCB059872 for 16 hours. **H**. Log2 fold-change in ATAC-Seq peaks at TSS + 3 kb (promoters) or gene body + 10 kb (enhancers) of GFI1 and PU.1 target genes in SET-2 cells treated with INCB059872 for 16 hours. **I**. OCI-AML5 cells were treated with 1000 nM of INCB059872 for 16 hours and cross-linked for H3K27Ac ChIP analysis. The number of active enhancers marked with H3K27Ac in OCI-AML5 cells treated with INCB059872 compared to control cells is shown. **J**. Ranked ordering of super enhancers (ROSE) analysis in INCB059872-treated OCI-AML5 cells as determined by H3K27Ac ChIP-Seq. **K**. Log2 fold-changes in H3K27Ac ChIP-Seq peaks at GFI1 and PU.1 target genes in INCB059872-treated versus control OCI-AML5 cells. **L**. Log2 fold-changes in BRD4 occupancy at TSS +/-3 kb in INCB059872-treated versus control OCI-AML5 cells. **M-N**. Log2 fold-change in BRD4 occupancy on GFI1 and PU.1 target genes in INCB059872-treated OCI-AML5 cells versus control cells.

**Supplemental Figure 9. Treatment with INCB059872 induces greater upregulation in mRNA expressions than depletion in AML and post-MPN sAML cells**. **A**. Heat map showing the number of RNA-Seq-determined mRNAs depleted or induced > 1.25 fold and p-value < 0.05 in OCI-AML5 (biologic triplicates) and SET2 (biologic duplicates) cells treated with INCB059872 for 16 hours. **B**. Gene set enrichment analysis of INCB059872-treated OCI-AML5 cells compared to HALLMARK pathways. All q-values for normalized enrichment scores were < 0.1. **C-D**. Representative GSEA plots for INCB059872-treated OCI-AML5 cells compared to GFI1 transcription factor target genes (GFI1_01) and to HALLMARK_MYC_TARGETS_V1 genes. **E**. Gene set enrichment analysis of INCB059872-treated SET-2 cells compared to HALLMARK pathways. All q-values for normalized enrichment scores were < 0.1. **F-G**. Log2 fold-changes in mRNA expression of GFI1 target genes in INCB059872-treated OCI-AML5 and SET2 cells as determined by RNA-Seq analysis. **H-I**. Log2 fold-changes in mRNA expression of PU.1 target genes in INCB059872-treated OCI-AML5 and SET2 cells as determined by RNA-Seq analysis. **J-K**. Log2 fold-changes in mRNA expression of CEBPα target genes in INCB059872-treated OCI-AML5 and SET2 cells as determined by RNA-Seq analysis.

**Supplemental Figure S10. Monitoring of GFP-positive cells from OC-AML5-Cas9 expressing cells transduced with domain-specific gRNAs against chromatin modifying proteins**. OCI-AML5 Cas9 cells transduced with a domain-specific gRNA library were analyzed for GFP expression in live cells by flow cytometry 2 and 12 days post transduction (with and without INCB059872 treatment for 96 hours). The % GFP-positive cells in each condition are shown.

**Supplemental Figure 11. Co-treatment with LSD1i and BET inhibitor (BETi) exerts synergistic lethal activity in AML and post-MPN, sAML cells**. **A**. Relative mRNA expression of OCI-AML5 cells treated (in biologic triplicates) with OTX015 for 8 hours or with INCB059872 for 16 hours prior to treatment with OTX015 for 8 hours. Expression of each mRNA was normalized to GAPDH and compared relative to the untreated cells. **B**. OCI-AML5 cells were treated with the indicated concentrations of OTX015 for 18 hours. Total cell lysates were harvested and immunoblot analyses were conducted. The expression levels of β-Actin in the lysates served as the loading control. Vertical bars indicate a repositioned gel image. **C**. Combination index (CI) values calculated for AML and sAML cells treated with INCB059872 (dose range: 100-1000 nM) and OTX015 (dose range: 125-1000 nM) for 48 hours. The % of annexin V-positive, To-Pro-3 iodide-positive, apoptotic cells was determined by flow cytometry. CI values < 1.0 indicate a synergistic interaction of the drugs in the combination. **D-E.** Immunoblot analysis of OCI-AML5 and SET2 cells treated with INCB059872 and/or OTX015 for 24 hours. The expression levels of β-Actin in the lysates served as the loading control. **F**. Combination index (CI) values calculated for AML and sAML cells treated with ORY1001 (dose range: 100-1000 nM) and OTX015 (dose range: 125-1000 nM) for 48 hours. The % of annexin V-positive, To-Pro-3 iodide-positive, apoptotic cells was determined by flow cytometry. CI values < 1.0 indicate a synergistic interaction of the drugs in the combination. **G**. SET2 cells were treated with the indicated concentrations of OTX015 with or without 2 days pretreatment with INCB059872. The % annexin V-positive, To-Pro-3 iodide-positive, apoptotic cells were determined by flow cytometry. Mean of three experiments + S. D.

**Supplemental Figure 12. Treatment with INCB059872 and BET inhibitor ABBV075 exhibits synergistic lethal activity in AML and post-MPN sAML cells. A**. Oncoplot of next generation sequencing (NGS)-detected mutations in cultured AML and sAML cell lines. **B**. OCI-AML5, MOLM13, SET-2 and HEL92.1.7 cells were treated with INCB059872 (dose range: 100-1000 nM) and/or ABBV075 (dose range: 10-250 nM) for 48 hours. At the end of treatment, the % of annexin V-positive, To-Pro-3 iodide-positive, apoptotic cells was determined by flow cytometry. Combination index (CI) values were calculated with CompuSyn. CI values < 1.0 indicate a synergistic interaction of the drugs in the combination. **C**. Oncoplot of next generation sequencing (NGS)-detected mutations in patient-derived de novo AML blasts. **D**. Oncoplot of next generation sequencing (NGS)-detected mutations in patient-derived, post-MPN/MF sAML blasts utilized in these studies.

**Supplemental Figure 13. Co-treatment with INCB059872 and HDAC3-specific inhibitor exerts synergistic anti-leukemia activity in AML and sAML cells.** **A**. OCI-AML5 LSD1-FKBP12^F36V^ cells were treated with 500 nM of dTAG-13 and/or the indicated concentrations of RGFP966 for 96 hours. The % of CD11b-positive cells was determined by flow cytometry. Mean of three experiments + S. D. *= p< 0.05 compared to cells treated with dTAG-13 alone (determined by two-tailed, unpaired t-test). **B-C**. OCI-AML5 cells were treated with the indicated concentrations of INCB059872 and/or HDAC3-specific inhibitor RGFP966 for 96 hours. The % of CD11b positive cells were determined by flow cytometry. Synergy of combinations (from two independent experiments) was determined utilizing SynergyFinder and the Bliss Synergy Score. Panel (**C**) shows the individual Bliss score for each combination. **D-E**. SET-2 cells were treated with the indicated concentrations of INCB059872 and/or HDAC3-specific inhibitor RGFP966 for 96 hours. The % of To-Pro-3 iodide-positive, non-viable cells were determined by flow cytometry. Synergy of combinations (from two independent experiments) was determined utilizing SynergyFinder and the Bliss Synergy Score. Panel (E) shows the individual Bliss score for each combination.

**Supplemental Figure 14. Co-treatment with INCB059872 and MOZ inhibitor WM1119 exerts synergistic anti-leukemia activity in AML and sAML cells**. **A**. OCI-AML5 LSD1-FKBP12^F36V^ cells were treated with 500 nM of dTAG-13 and/or the indicated concentrations of WM1119 for 96 hours. The % of CD11b-positive cells was determined by flow cytometry. Mean of three experiments + S. D. *= p< 0.05; **=p<0.01; *** = p<0.005 compared to cells treated with dTAG-13 alone (determined by two-tailed, unpaired t-test). **B-C**. OCI-AML5 cells were treated with the indicated concentrations of INCB059872 and/or MOZ inhibitor WM1119 for 96 hours. The % of CD11b positive cells were determined by flow cytometry. Synergy of combinations (from two independent experiments) was determined utilizing SynergyFinder and the Bliss Synergy Score. Panel (**C**) shows the individual Bliss score for each combination. **D-E**. SET-2 cells were treated with the indicated concentrations of INCB059872 and/or MOZ inhibitor for 96 hours. The % of To-Pro-3 iodide-positive, non-viable cells were determined by flow cytometry. Synergy of combinations (from two independent experiments) was determined utilizing SynergyFinder and the Bliss Synergy Score. Panel (**E**) shows the individual Bliss score for each combination. **F.** PD, sAML cells were treated with INCB059872 (dose range: 100-1000 nM) and/or WM1119 (dose range: 250-2000 nM) for 96 hours. The % of To-Pro-3 iodide-positive, non-viable cells was determined by flow cytometry. Combination index values were calculated with CompuSyn. Combination index values < 1.0 indicate a synergistic interaction of the drugs in the combination.

**Supplemental Figure 15. Co-treatment with LSD1 inhibitor and DOT1L inhibitor, decitabine or FLT3 antagonist, quizartinib (AC220) exerts synergistic lethal activity in AML cells. A**. OCI-AML5, SET-2, and PD, sAML cells were treated with EPZ5676 (dose range: 100-1000 nM) and/or INCB059872 (100-500 nM) for 96 hours. The % of CD11b positive cells (OCI-AML5) or the % of To-Pro-3 iodide positive cells (MOLM13, MV4-11, SET2, sAML17, and sAML19) were determined by flow cytometry. Combination index values were calculated with CompuSyn. Combination index values < 1.0 indicate a synergistic interaction of the drugs in the combination. **B-C**. MV4-11, MOLM13, THP1, and SKM1 cells were treated with INCB059872 (dose range: 100-1000 nM) or ORY1001 (dose range: 50-500 nM) and/or decitabine (100-500 nM) for 48 hours. At the end of treatment, the % of annexin V-positive, To-Pro-3 iodide-positive, apoptotic cells was determined by flow cytometry. CI values < 1.0 indicate a synergistic interaction of the drugs in the combination.

**Supplemental Figure 16**. **Pre-treatment with LSD1i ORY1001 re-sensitizes BETi persister/resistant post-MPN sAML and de novo AML blast cells to BETi treatment**. **A**. SET-2, HEL92.1.7, THP1 and their BETi-persistent derivatives were treated with the indicated concentrations of OTX015 for 48 hours. The % of annexin V-positive, apoptotic cells was determined by flow cytometry. Mean of three experiments + S. D. **B**. HEL92.1.7, HEL-OTX P/R, SET-2 and SET-2-OTX P/R cells were treated with INCB059872 for 48 hours. The % of annexin V-positive, apoptotic cells was determined by flow cytometry. Mean of three experiments + S. D. **C-D**. SET-2 OTX P/R and THP1 OTX P/R were treated with the indicated concentrations of OTX015 with or without 2 days of pre-treatment with 250 nM of ORY1001. The % of annexin V-positive, apoptotic cells was determined by flow cytometry. Mean of three experiments + S. D. *** = p<0.005; **** = p<0.001 compared to cells treated with OTX015 without ORY1001 pre-treatment (determined by two-tailed, unpaired t-test). **E**. SET-2-OTX P/R cells were treated (in biologic triplicates) with INCB059872 and OTX015, as indicated. Total RNA was isolated and reverse-transcribed. The resulting cDNA was utilized for qPCR analysis. GAPDH was utilized as the normalization control and the relative mRNA expression of each target is compared to the untreated control cells. * = p<0.05, ** = p<0.01 compared to cells treated with INCB059872 alone (determined by two-tailed, unpaired t-test).

**Supplemental Figure 17. Treatment with INCB059872 alters chromatin accessibility and mRNA expression of GFI1, PU.1, CEBPα, IRF8, and MYC target genes in BET inhibitor persister/resistant SET-2-OTX P/R cells. A-C**. Log2 fold-change in ATAC-Seq peaks at TSS + 3 kb (promoters) or gene body + 10 kb (enhancers) of GFI1, PU.1, and IRF8 target genes in SET-2-OTX P/R cells treated with 250 nM of INCB059872 for 16 hours. **D-G**. Log2 fold-changes (as determined by RNA Seq analysis: > 1.25 fold and p-value < 0.05 in INCB059872-treated compared to untreated control cells) in GFI1, PU.1, MYC, and CEBPα target genes in BET inhibitor persister-resistant SET-2-OTX P/R cells treated with INCB059872 for 16 hours.

**Supplemental Figure 18. Treatment with INCB059872 reduces leukemia burden and improves survival of NSG mice engrafted with sAML xenografts**. **A**. Total photon counts [flux] (determined by bioluminescent imaging) in NSG mice engrafted with HEL92.1.7 GFP-Luc cells and treated for 2 weeks with INCB059872. **B**. Kaplan-Meier survival plot of NSG mice engrafted with HEL92.1.7 GFP-Luc cells and treated with 1.5 mg/kg of INCB059872 (daily x 5 days, P.O.) for 3 weeks. Significance was calculated by Mantel-Cox log-rank test.
