# Supplementary material for: Superior efficacy of co-targeting GFI1/KDM1A and BRD4 against AML and post-MPN secondary AML cells
Source: Blood Cancer J. 2021 May 20;11(5):98. doi: 10.1038/s41408-021-00487-3 (PMC8138012; doi:10.1038/s41408-021-00487-3)
Supplement: Supplementary file 3 — Supplemental Figures [file 41408_2021_487_MOESM3_ESM.pdf]

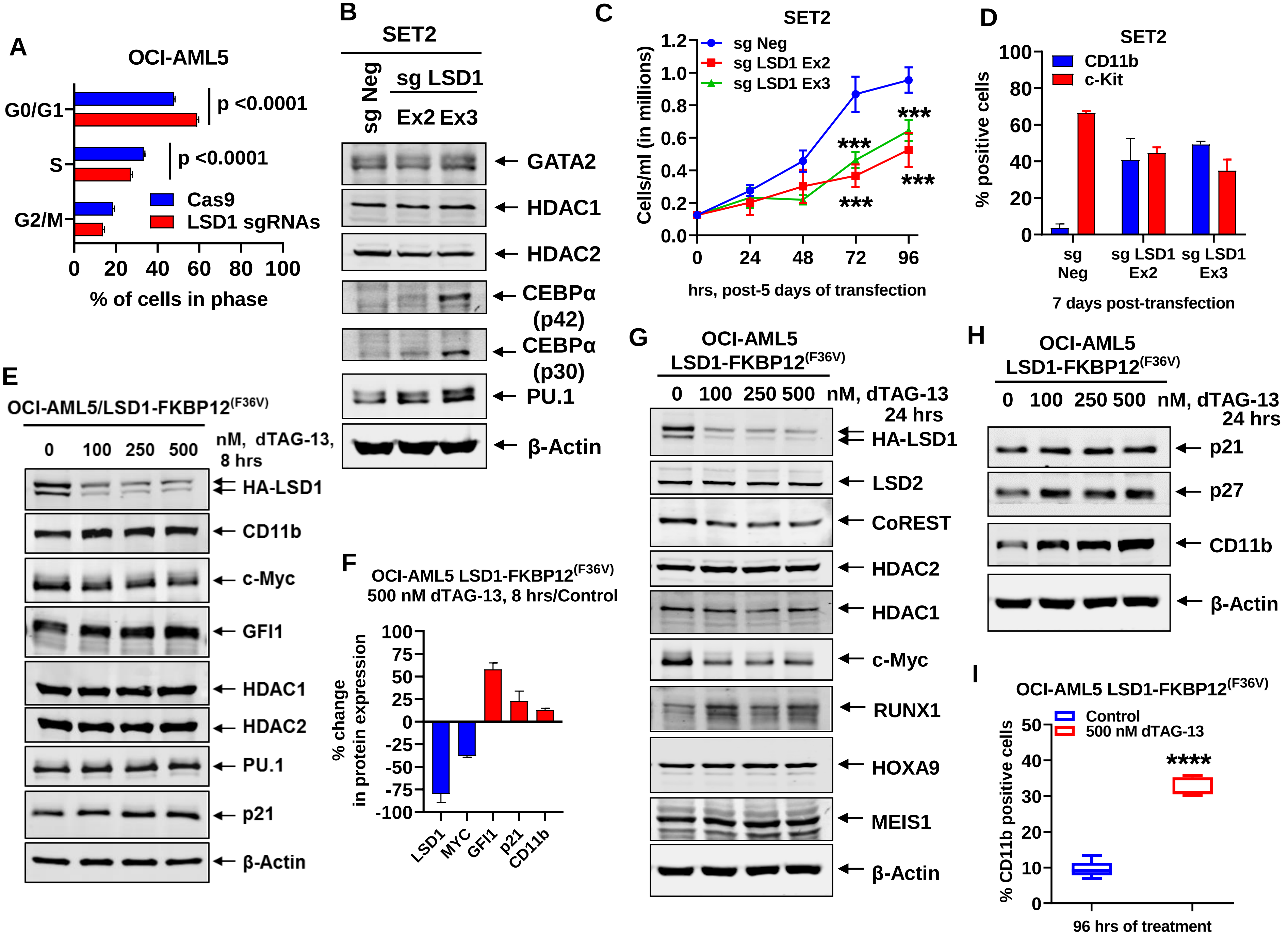
A
OCI-AML5
G0/G1
S
G2/M
p <0.0001
p <0.0001
Cas9
LSD1 sgRNAs
0 20 40 60 80 100
% of cells in phase
B
SET2
sg Neg
sg LSD1
Ex2 Ex3
GATA2
HDAC1
HDAC2
CEBPα (p42)
CEBPα (p30)
PU.1
β-Actin
C
SET2
sg Neg
sg LSD1 Ex2
sg LSD1 Ex3
Cells/ml (in millions)
***
***
***
***
hrs, post-5 days of transfection
D
SET2
CD11b
c-Kit
% positive cells
sg Neg
sg LSD1 Ex2
sg LSD1 Ex3
7 days post-transfection
E
OCI-AML5/LSD1-FKBP12(F36V)
0 100 250 500
nM, dTAG-13, 8 hrs
HA-LSD1
CD11b
c-Myc
GFI1
HDAC1
HDAC2
PU.1
p21
β-Actin
F
OCI-AML5 LSD1-FKBP12(F36V)
500 nM dTAG-13, 8 hrs/Control
% change in protein expression
LSD1
MYC
GFI1
p21
CD11b
G
OCI-AML5
LSD1-FKBP12(F36V)
0 100 250 500 nM, dTAG-13
24 hrs
HA-LSD1
LSD2
CoREST
HDAC2
HDAC1
c-Myc
RUNX1
HOXA9
MEIS1
β-Actin
H
OCI-AML5
LSD1-FKBP12(F36V)
0 100 250 500 nM, dTAG-13
24 hrs
p21
p27
CD11b
β-Actin
I
OCI-AML5 LSD1-FKBP12(F36V)
Control
500 nM dTAG-13
% CD11b positive cells
****
96 hrs of treatment

Figure S1

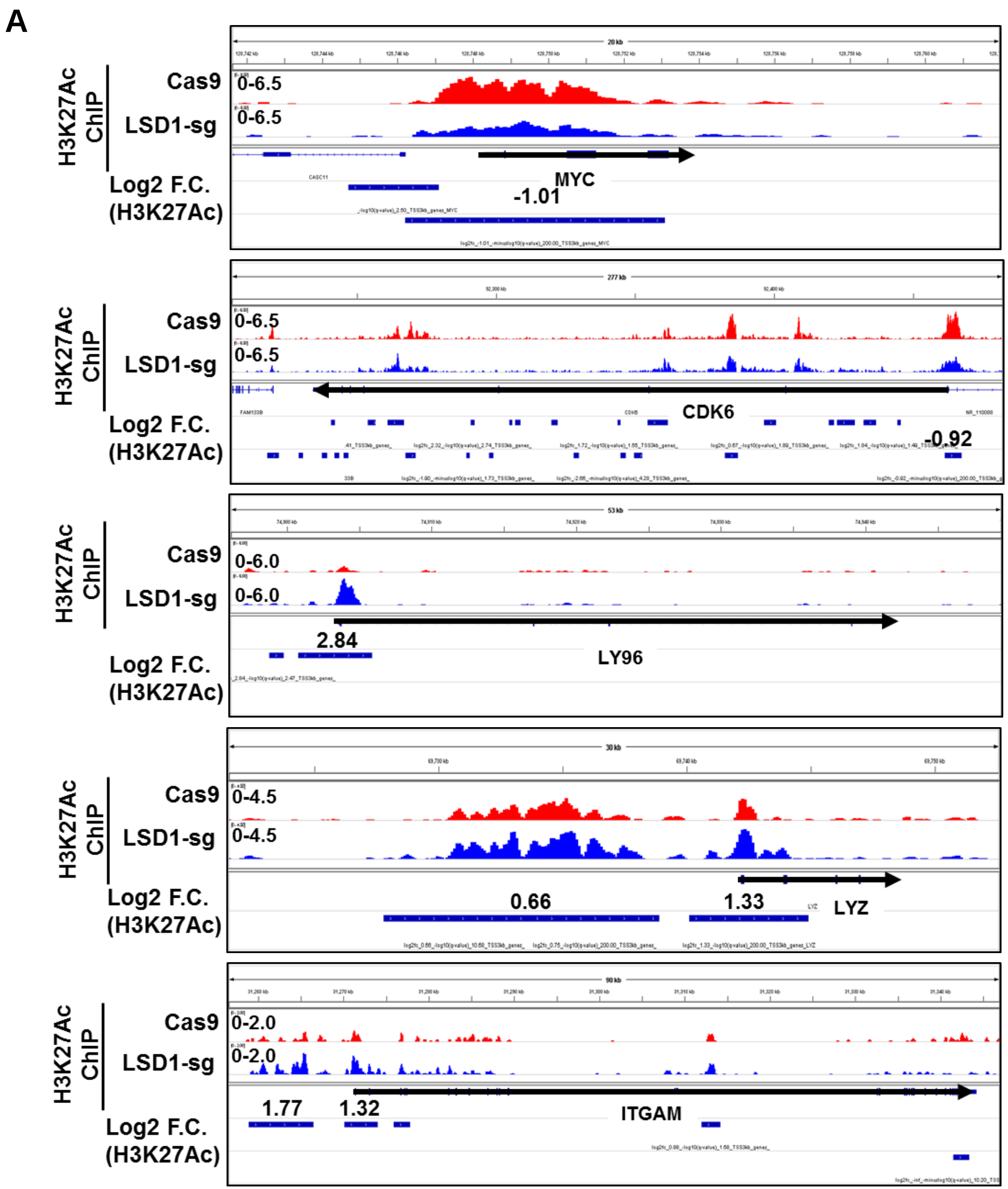

A
H3K27Ac ChIP
Cas9
0-6.5
LSD1-sg
0-6.5
Log2 F.C. (H3K27Ac)
MYC
-1.01
20 kb
H3K27Ac ChIP
Cas9
0-6.5
LSD1-sg
0-6.5
Log2 F.C. (H3K27Ac)
CDK6
-0.92
277 kb
H3K27Ac ChIP
Cas9
0-6.0
LSD1-sg
0-6.0
Log2 F.C. (H3K27Ac)
2.84
LY96
53 kb
H3K27Ac ChIP
Cas9
0-4.5
LSD1-sg
0-4.5
Log2 F.C. (H3K27Ac)
0.66
1.33
LYZ
30 kb
H3K27Ac ChIP
Cas9
0-2.0
LSD1-sg
0-2.0
Log2 F.C. (H3K27Ac)
1.77
1.32
ITGAM
90 kb

Figure S2

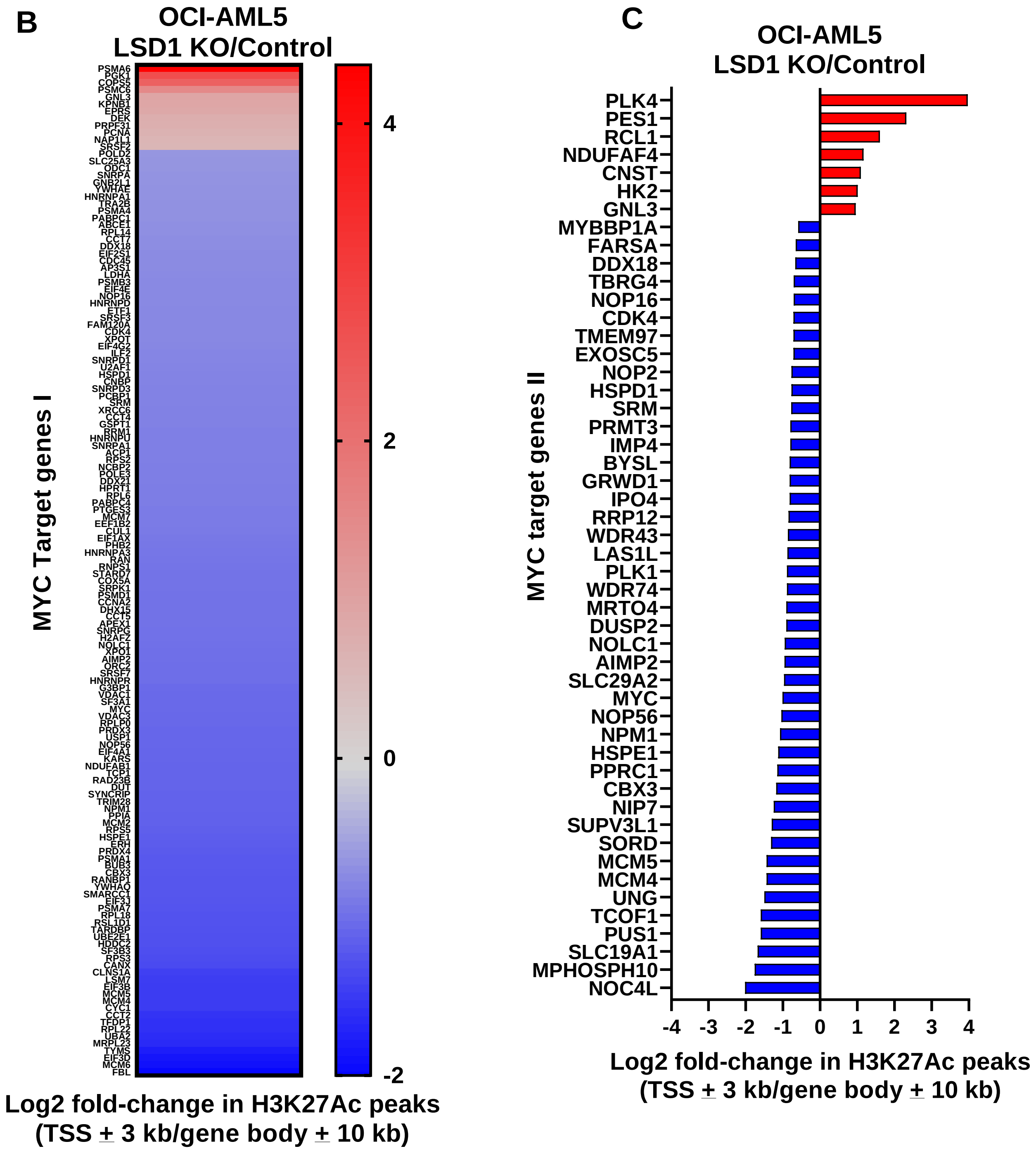
B
OCI-AML5
LSD1 KO/Control
MYC Target genes I
PSMA6
PGK1
COPS5
PSMC6
GNL3
KPNB1
EPRS
DEK
PRPF31
PCNA
NAP1L1
SRSF2
POLD2
SLC25A3
ODC1
SNRPA
GNB2L1
YWHAE
HNRNPA1
TRA2B
PSMA4
PABPC1
ABCE1
RPL14
CCT7
DDX18
EIF2S1
CDC45
AP3S1
LDHA
PSMB3
EIF4E
NOP16
HNRNPD
ETF1
SRSF3
FAM120A
CDK4
XPOT
EIF4G2
ILF2
SNRPD1
U2AF1
HSPD1
CNBP
SNRPD3
PCBP1
SRM
XRCC6
CCT4
GSPT1
RRM1
HNRNPU
SNRPA1
ACP1
RPS2
NCBP2
POLE3
DDX21
HPRT1
RPL6
PABPC4
PTGES3
MCM7
EEF1B2
CUL1
EIF1AX
PHB2
HNRNPA3
RAN
RNPS1
STARD7
COX5A
SRPK1
PSMD1
CCNA2
DHX15
CCT5
APEX1
SNRPG
H2AFZ
NOLC1
XPO1
AIMP2
ORC2
SRSF7
HNRNPR
G3BP1
VDAC1
SF3A1
MYC
VDAC3
RPLP0
PRDX3
USP1
NOP56
EIF4A1
KARS
NDUFAB1
TCP1
RAD23B
DUT
SYNCRIP
TRIM28
NPM1
PPIA
MCM2
RPS5
HSPE1
ERH
PRDX4
PSMA1
BUB3
CBX3
RANBP1
YWHAQ
SMARCC1
EIF3J
PSMA7
RPL18
RSL1D1
TARDBP
UBE2E1
HDDC2
SF3B3
RPS3
CANX
CLNS1A
LSM7
EIF3B
MCM5
MCM4
CYC1
CCT2
TFDP1
RPL22
UBA2
MRPL23
TYMS
EIF3D
MCM6
FBL
4
2
0
-2
Log2 fold-change in H3K27Ac peaks
(TSS ± 3 kb/gene body ± 10 kb)
C
OCI-AML5
LSD1 KO/Control
MYC target genes II
PLK4
PES1
RCL1
NDUFAF4
CNST
HK2
GNL3
MYBBP1A
FARSA
DDX18
TBRG4
NOP16
CDK4
TMEM97
EXOSC5
NOP2
HSPD1
SRM
PRMT3
IMP4
BYSL
GRWD1
IPO4
RRP12
WDR43
LAS1L
PLK1
WDR74
MRTO4
DUSP2
NOLC1
AIMP2
SLC29A2
MYC
NOP56
NPM1
HSPE1
PPRC1
CBX3
NIP7
SUPV3L1
SORD
MCM5
MCM4
UNG
TCOF1
PUS1
SLC19A1
MPHOSPH10
NOC4L
-4
-3
-2
-1
0
1
2
3
4
Log2 fold-change in H3K27Ac peaks
(TSS ± 3 kb/gene body ± 10 kb)

Figure S2

D

BRD4 and H3K27Ac occupancy on the GFI1 gene and super enhancer following LSD1 knockout in OCI-AML5 cells

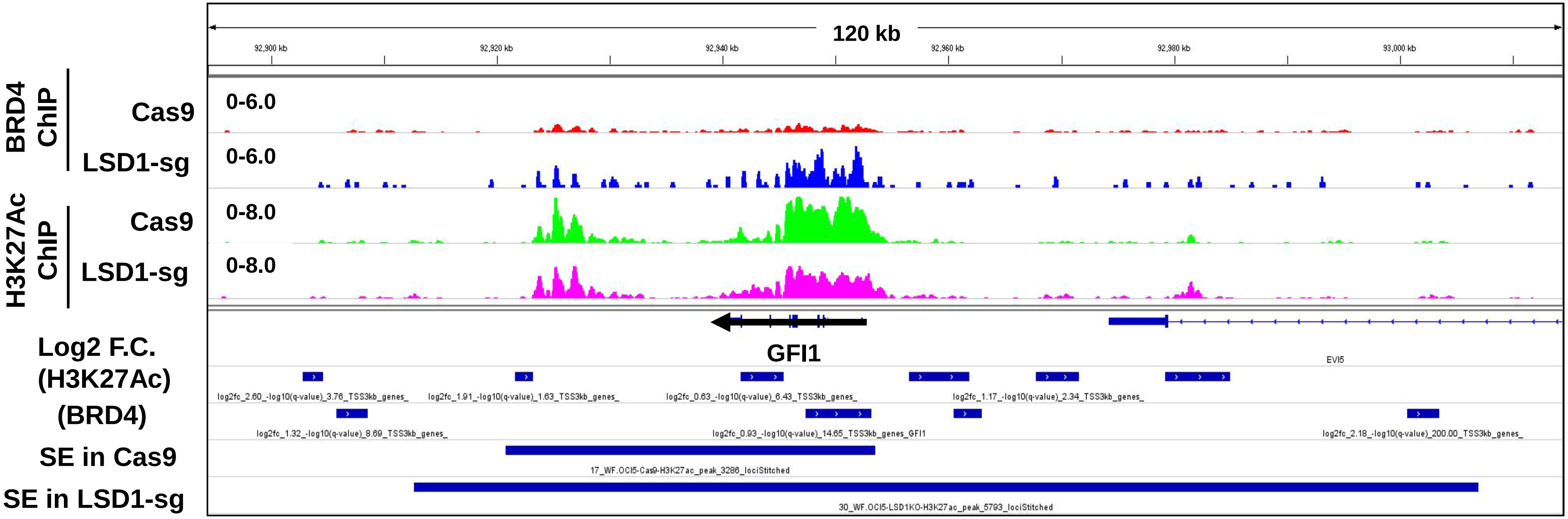

Figure S2

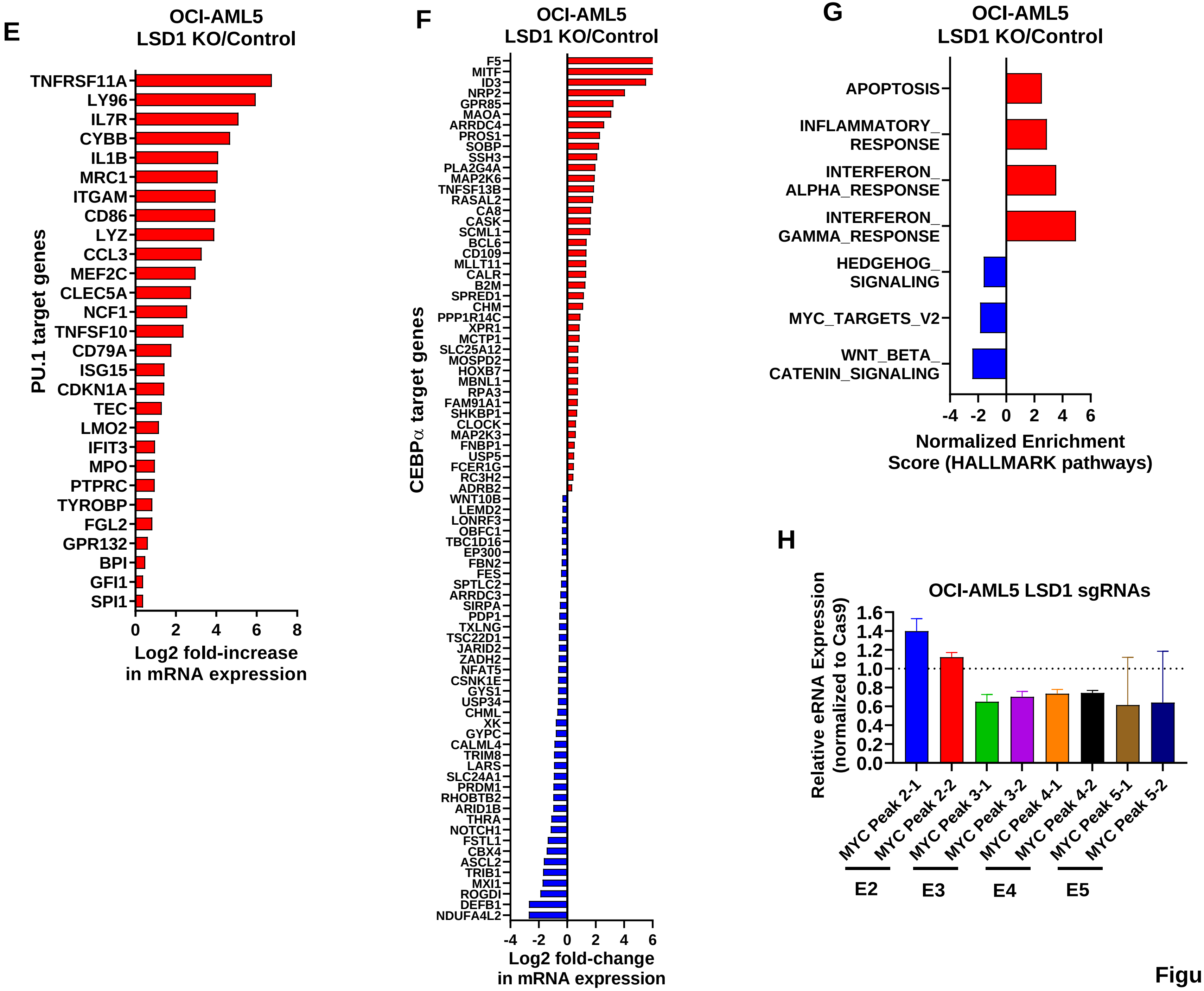
E
OCI-AML5
LSD1 KO/Control
PU.1 target genes
TNFRSF11A
LY96
IL7R
CYBB
IL1B
MRC1
ITGAM
CD86
LYZ
CCL3
MEF2C
CLEC5A
NCF1
TNFSF10
CD79A
ISG15
CDKN1A
TEC
LMO2
IFIT3
MPO
PTPRC
TYROBP
FGL2
GPR132
BPI
GFI1
SPI1
0 2 4 6 8
Log2 fold-increase
in mRNA expression
F
OCI-AML5
LSD1 KO/Control
CEBPα target genes
F5
MITF
ID3
NRP2
GPR85
MAOA
ARRDC4
PROS1
SOBP
SSH3
PLA2G4A
MAP2K6
TNFSF13B
RASAL2
CA8
CASK
SCML1
BCL6
CD109
MLLT11
CALR
B2M
SPRED1
CHM
PPP1R14C
XPR1
MCTP1
SLC25A12
MOSPD2
HOXB7
MBNL1
RPA3
FAM91A1
SHKBP1
CLOCK
MAP2K3
FNBP1
USP5
FCER1G
RC3H2
ADRB2
WNT10B
LEMD2
LONRF3
OBFC1
TBC1D16
EP300
FBN2
FES
SPTLC2
ARRDC3
SIRPA
PDP1
TXLNG
TSC22D1
JARID2
ZADH2
NFAT5
CSNK1E
GYS1
USP34
CHML
XK
GYPC
CALML4
TRIM8
LARS
SLC24A1
PRDM1
RHOBTB2
ARID1B
THRA
NOTCH1
FSTL1
CBX4
ASCL2
TRIB1
MXI1
ROGDI
DEFB1
NDUFA4L2
-4 -2 0 2 4 6
Log2 fold-change
in mRNA expression
G
OCI-AML5
LSD1 KO/Control
APOPTOSIS
INFLAMMATORY_
RESPONSE
INTERFERON_
ALPHA_RESPONSE
INTERFERON_
GAMMA_RESPONSE
HEDGEHOG_
SIGNALING
MYC_TARGETS_V2
WNT_BETA_
CATENIN_SIGNALING
-4 -2 0 2 4 6
Normalized Enrichment
Score (HALLMARK pathways)
H
OCI-AML5 LSD1 sgRNAs
Relative eRNA Expression
(normalized to Cas9)
1.6
1.4
1.2
1.0
0.8
0.6
0.4
0.2
0.0
MYC Peak 2-1
MYC Peak 2-2
MYC Peak 3-1
MYC Peak 3-2
MYC Peak 4-1
MYC Peak 4-2
MYC Peak 5-1
MYC Peak 5-2
E2
E3
E4
E5

Figure S2

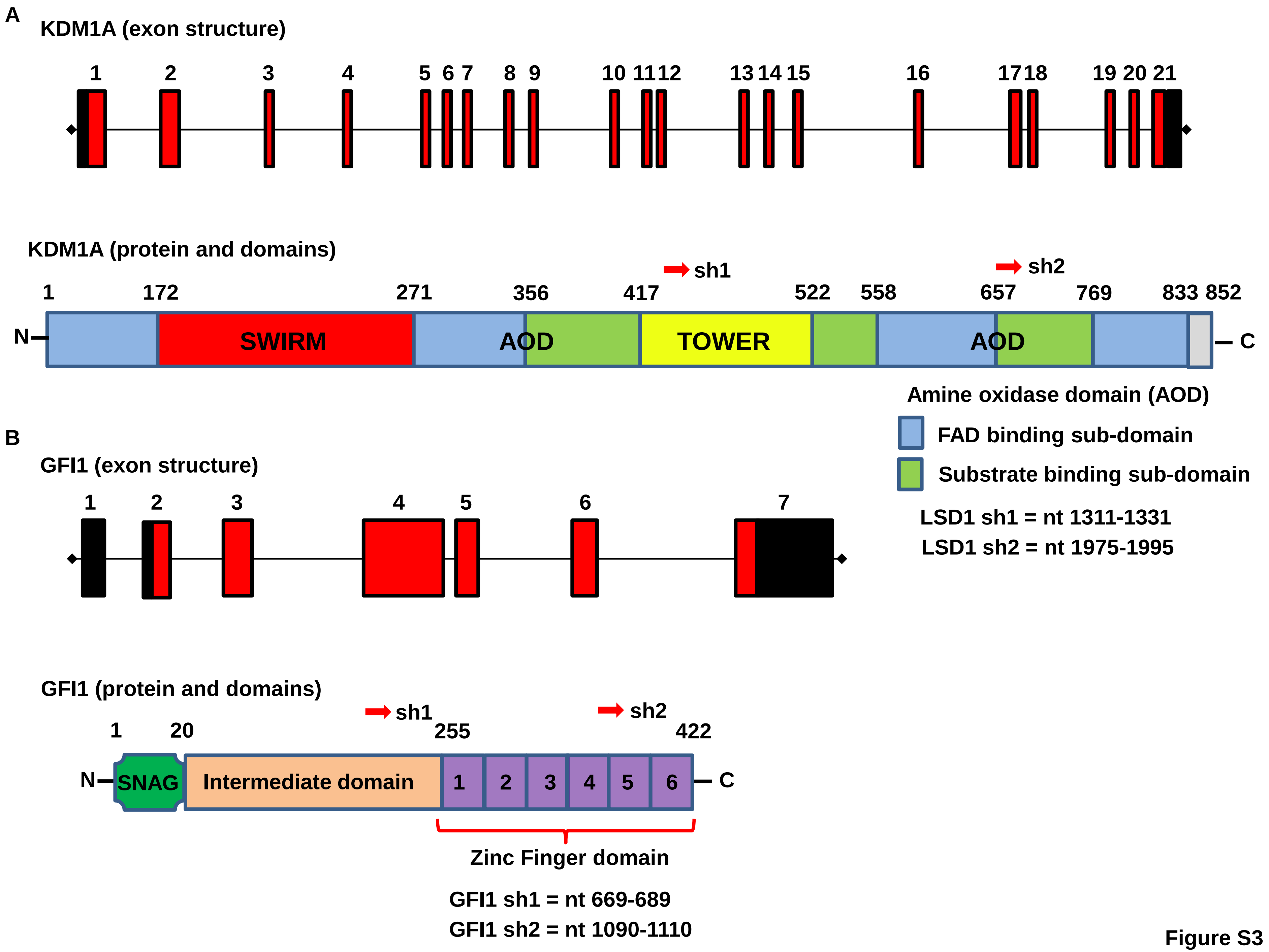
A
KDM1A (exon structure)
1 2 3 4 5 6 7 8 9 10 11 12 13 14 15 16 17 18 19 20 21
KDM1A (protein and domains)
sh1
sh2
1 172 271 356 417 522 558 657 769 833 852
N
SWIRM
AOD
TOWER
AOD
C
Amine oxidase domain (AOD)
FAD binding sub-domain
Substrate binding sub-domain
LSD1 sh1 = nt 1311-1331
LSD1 sh2 = nt 1975-1995
B
GFI1 (exon structure)
1 2 3 4 5 6 7
GFI1 (protein and domains)
sh1
sh2
1 20 255 422
N
SNAG
Intermediate domain
1 2 3 4 5 6
C
Zinc Finger domain
GFI1 sh1 = nt 669-689
GFI1 sh2 = nt 1090-1110

Figure S3

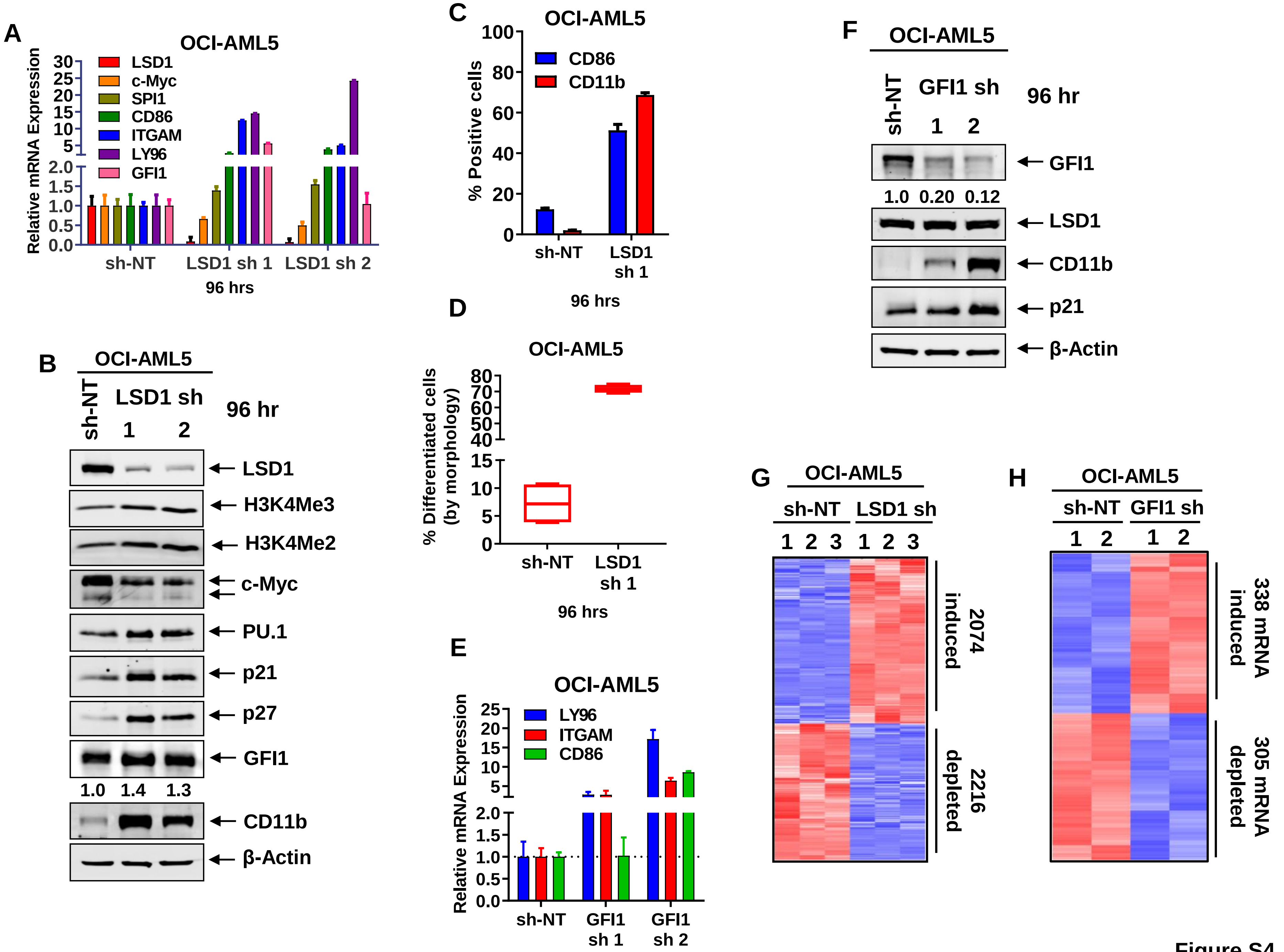
A
OCI-AML5
Relative mRNA Expression
LSD1
c-Myc
SPI1
CD86
ITGAM
LY96
GFI1
sh-NT
LSD1 sh 1
LSD1 sh 2
96 hrs
B
OCI-AML5
sh-NT
LSD1 sh
1
2
96 hr
LSD1
H3K4Me3
H3K4Me2
c-Myc
PU.1
p21
p27
GFI1
1.0
1.4
1.3
CD11b
β-Actin
C
OCI-AML5
% Positive cells
CD86
CD11b
sh-NT
LSD1 sh 1
96 hrs
D
OCI-AML5
% Differentiated cells (by morphology)
sh-NT
LSD1 sh 1
96 hrs
E
OCI-AML5
Relative mRNA Expression
LY96
ITGAM
CD86
sh-NT
GFI1 sh 1
GFI1 sh 2
F
OCI-AML5
sh-NT
GFI1 sh
1
2
96 hr
GFI1
1.0
0.20
0.12
LSD1
CD11b
p21
β-Actin
G
OCI-AML5
sh-NT
LSD1 sh
1 2 3
1 2 3
2074 induced
2216 depleted
H
OCI-AML5
sh-NT
GFI1 sh
1 2
1 2
338 mRNA induced
305 mRNA depleted

Figure S4

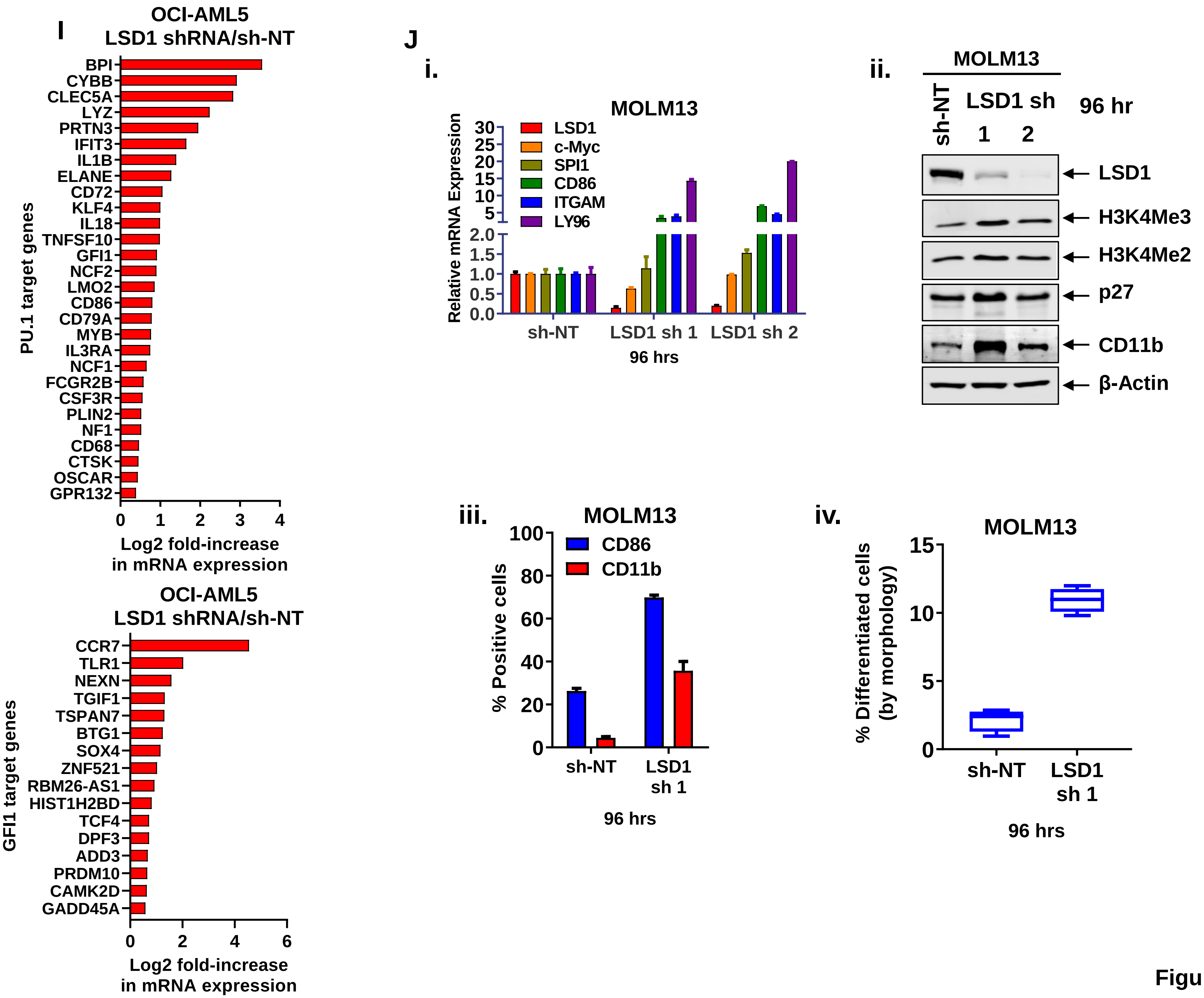
I
OCI-AML5
LSD1 shRNA/sh-NT
BPI
CYBB
CLEC5A
LYZ
PRTN3
IFIT3
IL1B
ELANE
CD72
KLF4
IL18
TNFSF10
GFI1
NCF2
LMO2
CD86
CD79A
MYB
IL3RA
NCF1
FCGR2B
CSF3R
PLIN2
NF1
CD68
CTSK
OSCAR
GPR132
PU.1 target genes
0 1 2 3 4
Log2 fold-increase
in mRNA expression
OCI-AML5
LSD1 shRNA/sh-NT
CCR7
TLR1
NEXN
TGIF1
TSPAN7
BTG1
SOX4
ZNF521
RBM26-AS1
HIST1H2BD
TCF4
DPF3
ADD3
PRDM10
CAMK2D
GADD45A
GFI1 target genes
0 2 4 6
Log2 fold-increase
in mRNA expression
J
i.
MOLM13
Relative mRNA Expression
30 25 20 15 10 5
2.0 1.5 1.0 0.5 0.0
LSD1
c-Myc
SPI1
CD86
ITGAM
LY96
sh-NT
LSD1 sh 1
LSD1 sh 2
96 hrs
ii.
MOLM13
sh-NT
LSD1 sh
1
2
96 hr
LSD1
H3K4Me3
H3K4Me2
p27
CD11b
β-Actin
iii.
MOLM13
% Positive cells
100 80 60 40 20 0
CD86
CD11b
sh-NT
LSD1
sh 1
96 hrs
iv.
MOLM13
% Differentiated cells
(by morphology)
15 10 5 0
sh-NT
LSD1
sh 1
96 hrs

Figure S4

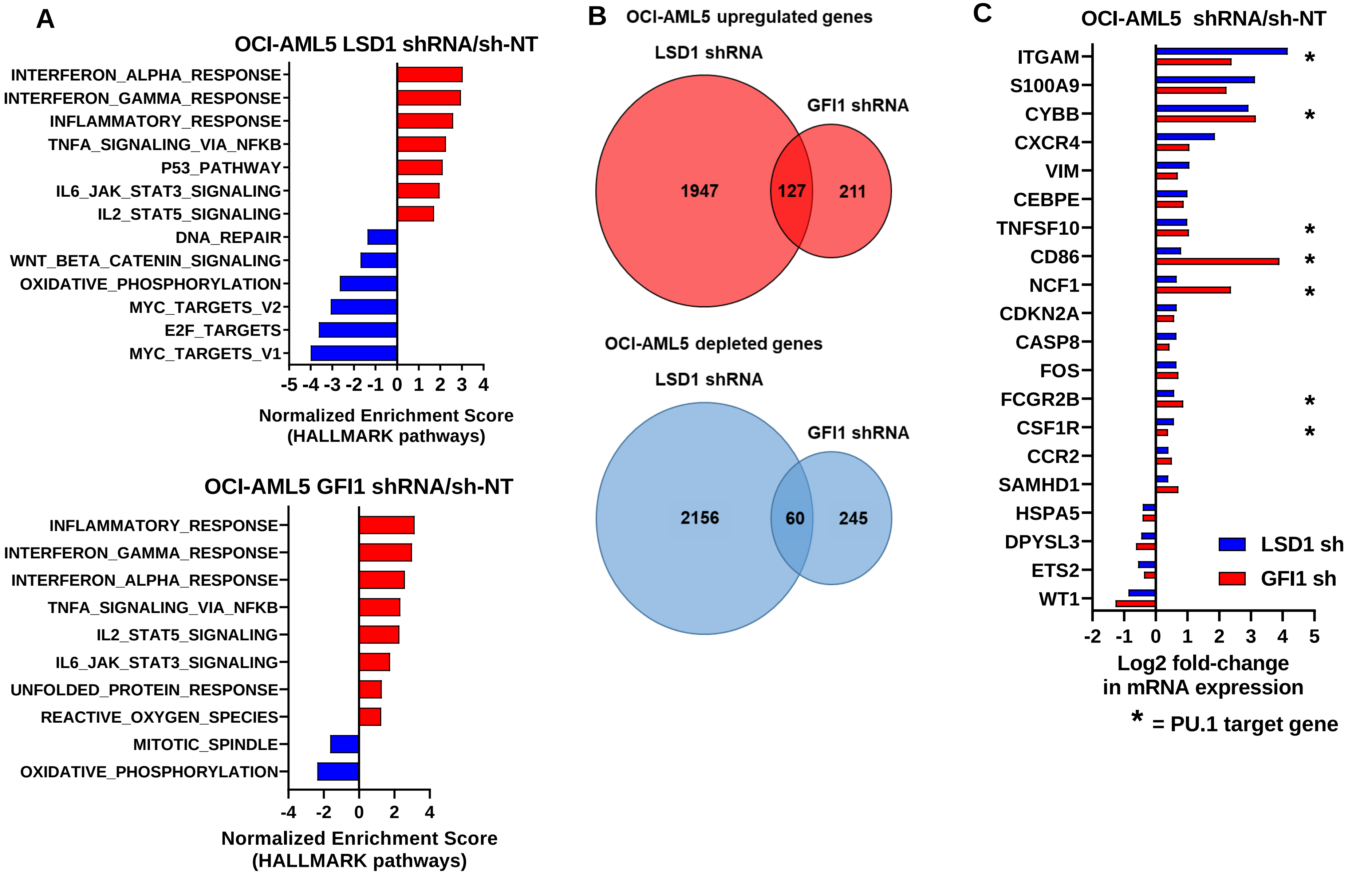
A
OCI-AML5 LSD1 shRNA/sh-NT
INTERFERON_ALPHA_RESPONSE
INTERFERON_GAMMA_RESPONSE
INFLAMMATORY_RESPONSE
TNFA_SIGNALING_VIA_NFKB
P53_PATHWAY
IL6_JAK_STAT3_SIGNALING
IL2_STAT5_SIGNALING
DNA_REPAIR
WNT_BETA_CATENIN_SIGNALING
OXIDATIVE_PHOSPHORYLATION
MYC_TARGETS_V2
E2F_TARGETS
MYC_TARGETS_V1
-5 -4 -3 -2 -1 0 1 2 3 4
Normalized Enrichment Score
(HALLMARK pathways)
OCI-AML5 GFI1 shRNA/sh-NT
INFLAMMATORY_RESPONSE
INTERFERON_GAMMA_RESPONSE
INTERFERON_ALPHA_RESPONSE
TNFA_SIGNALING_VIA_NFKB
IL2_STAT5_SIGNALING
IL6_JAK_STAT3_SIGNALING
UNFOLDED_PROTEIN_RESPONSE
REACTIVE_OXYGEN_SPECIES
MITOTIC_SPINDLE
OXIDATIVE_PHOSPHORYLATION
-4 -2 0 2 4
Normalized Enrichment Score
(HALLMARK pathways)
B
OCI-AML5 upregulated genes
LSD1 shRNA
GFI1 shRNA
1947
127
211
OCI-AML5 depleted genes
LSD1 shRNA
GFI1 shRNA
2156
60
245
C
OCI-AML5 shRNA/sh-NT
ITGAM
S100A9
CYBB
CXCR4
VIM
CEBPE
TNFSF10
CD86
NCF1
CDKN2A
CASP8
FOS
FCGR2B
CSF1R
CCR2
SAMHD1
HSPA5
DPYSL3
ETS2
WT1
*
*
*
*
*
*
*
LSD1 sh
GFI1 sh
-2 -1 0 1 2 3 4 5
Log2 fold-change
in mRNA expression
* = PU.1 target gene

Figure S5

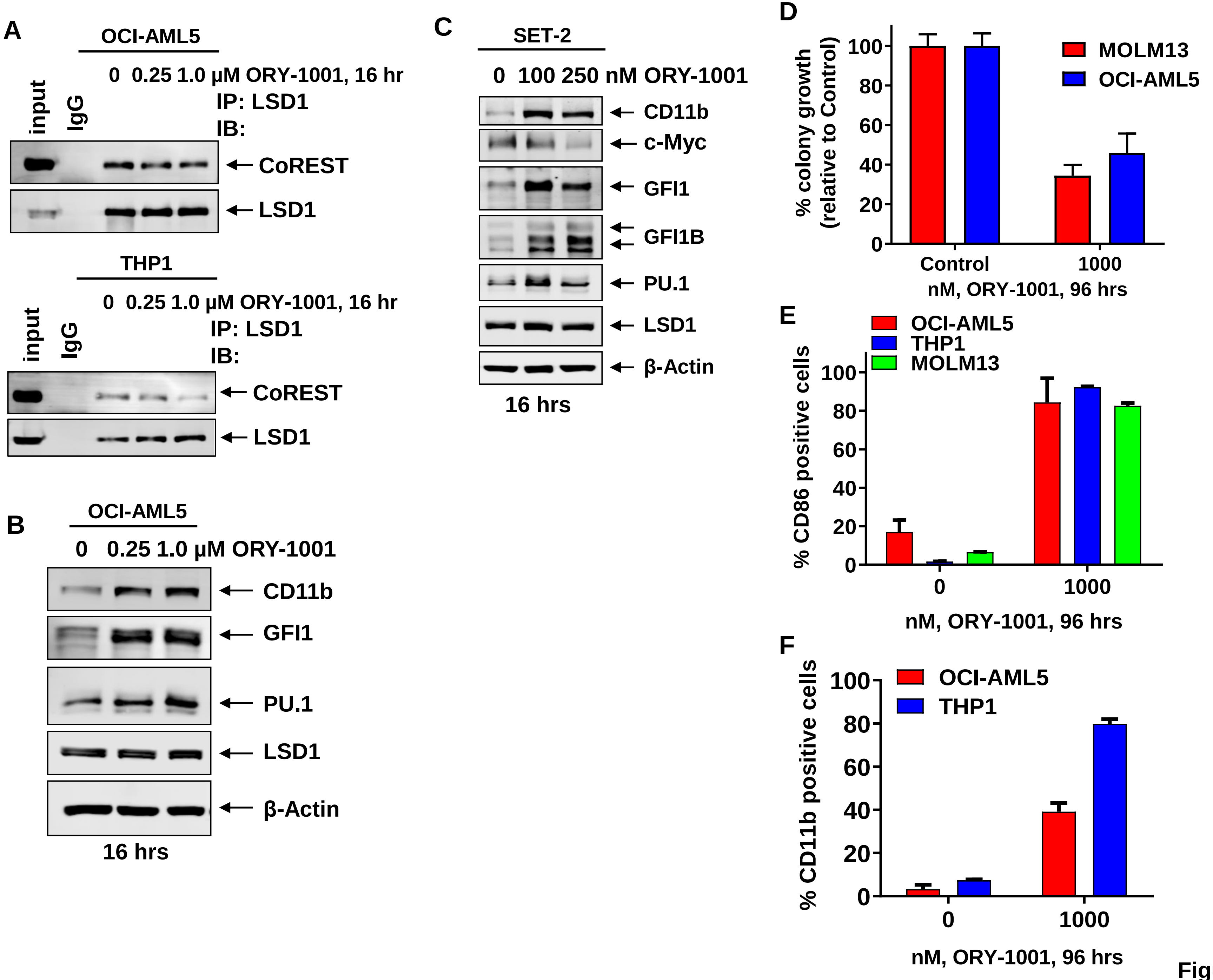

A
OCI-AML5
0 0.25 1.0 μM ORY-1001, 16 hr
IP: LSD1
IB:
input
IgG
CoREST
LSD1
THP1
0 0.25 1.0 μM ORY-1001, 16 hr
IP: LSD1
IB:
input
IgG
CoREST
LSD1
B
OCI-AML5
0 0.25 1.0 μM ORY-1001
CD11b
GFI1
PU.1
LSD1
β-Actin
16 hrs
C
SET-2
0 100 250 nM ORY-1001
CD11b
c-Myc
GFI1
GFI1B
PU.1
LSD1
β-Actin
16 hrs
D
% colony growth (relative to Control)
MOLM13
OCI-AML5
Control
1000
nM, ORY-1001, 96 hrs
E
OCI-AML5
THP1
MOLM13
% CD86 positive cells
0
1000
nM, ORY-1001, 96 hrs
F
OCI-AML5
THP1
% CD11b positive cells
0
1000
nM, ORY-1001, 96 hrs

Figure S6

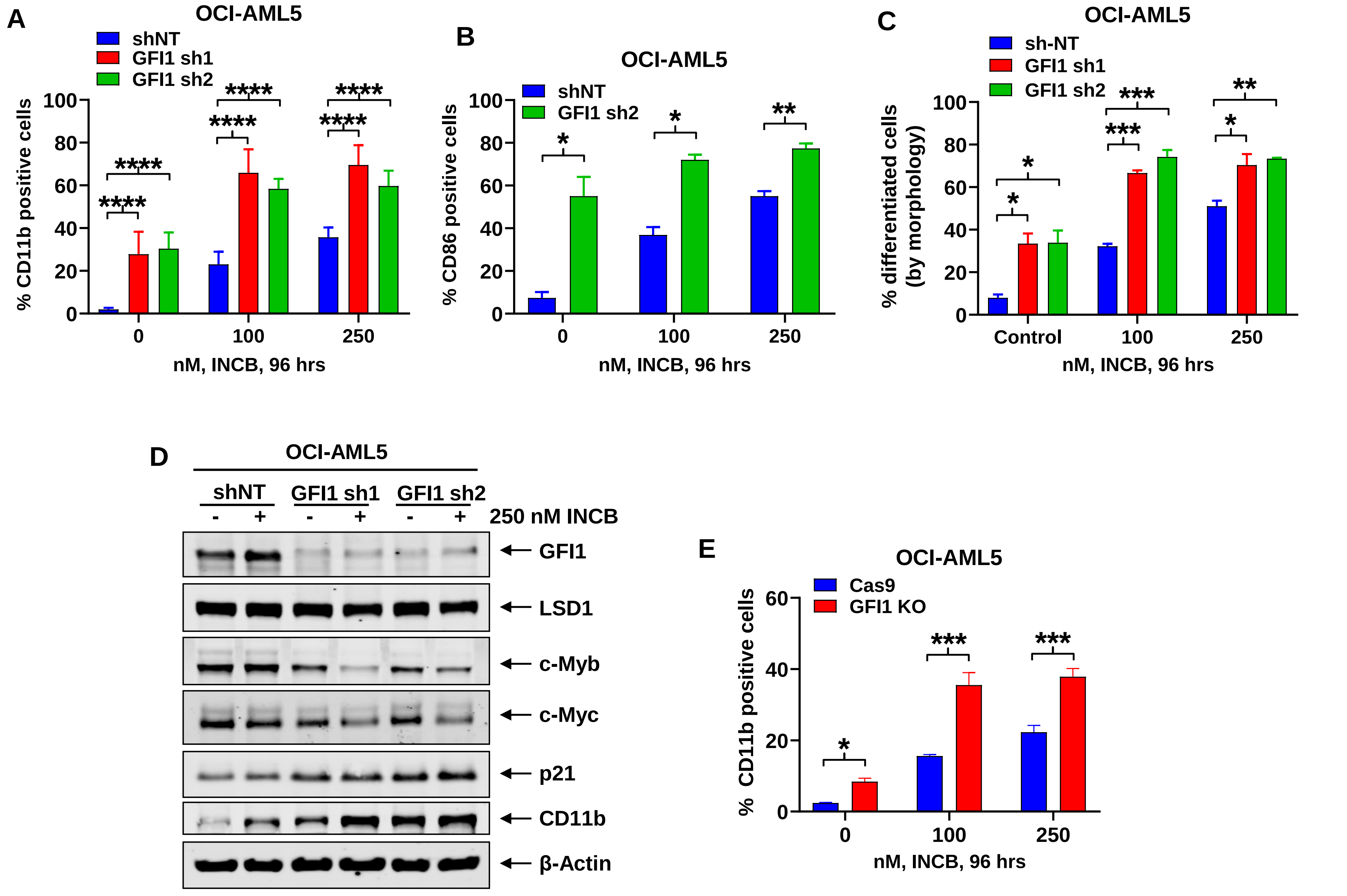
A
OCI-AML5
shNT
GFI1 sh1
GFI1 sh2
% CD11b positive cells
nM, INCB, 96 hrs
B
OCI-AML5
shNT
GFI1 sh2
% CD86 positive cells
nM, INCB, 96 hrs
C
OCI-AML5
sh-NT
GFI1 sh1
GFI1 sh2
% differentiated cells (by morphology)
Control
nM, INCB, 96 hrs
D
OCI-AML5
shNT
GFI1 sh1
GFI1 sh2
250 nM INCB
GFI1
LSD1
c-Myb
c-Myc
p21
CD11b
β-Actin
E
OCI-AML5
Cas9
GFI1 KO
% CD11b positive cells
nM, INCB, 96 hrs

Figure S7

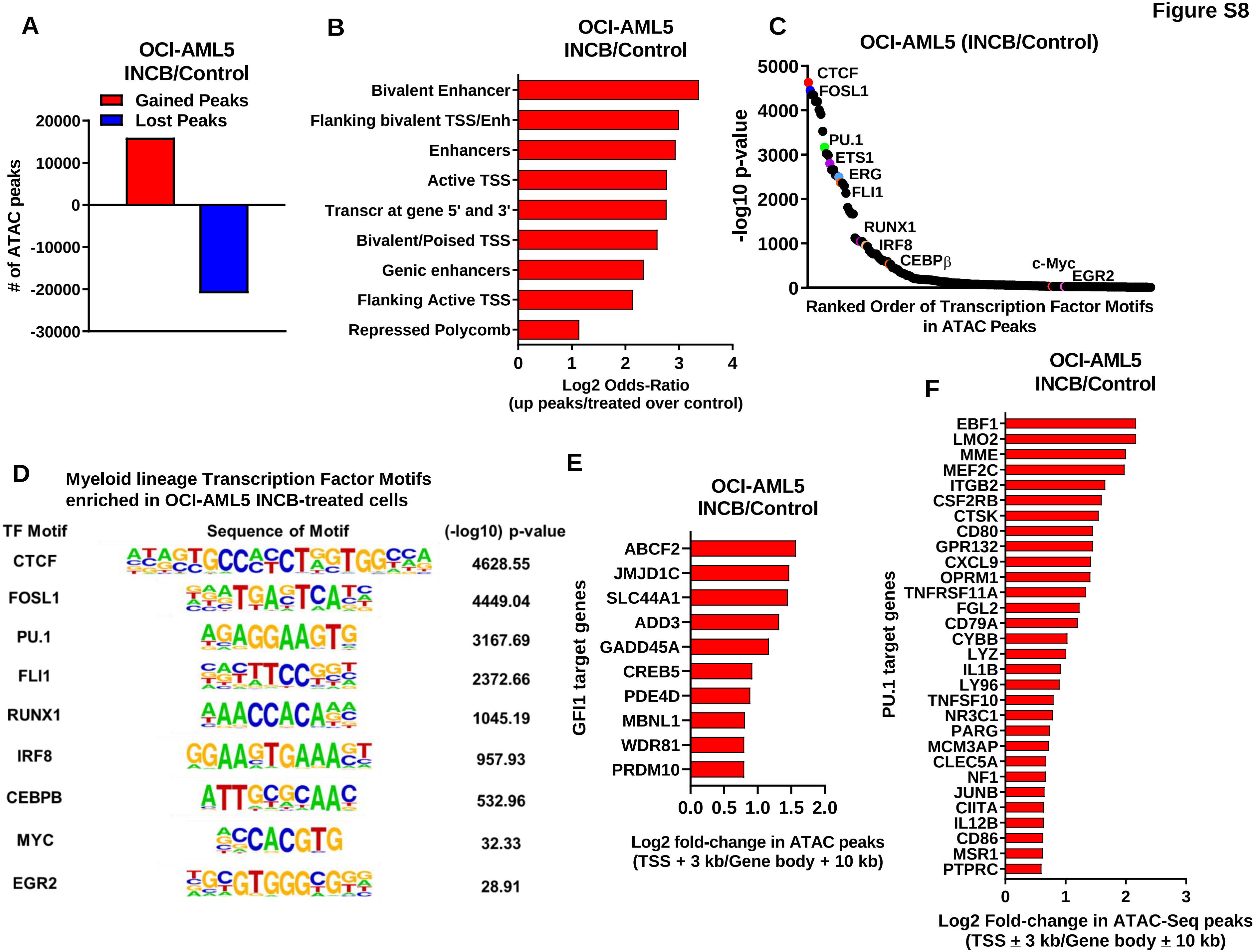

A
OCI-AML5 INCB/Control
Gained Peaks
Lost Peaks
# of ATAC peaks
20000
10000
0
-10000
-20000
-30000
B
OCI-AML5 INCB/Control
Bivalent Enhancer
Flanking bivalent TSS/Enh
Enhancers
Active TSS
Transcr at gene 5' and 3'
Bivalent/Poised TSS
Genic enhancers
Flanking Active TSS
Repressed Polycomb
0
1
2
3
4
Log2 Odds-Ratio (up peaks/treated over control)
C
OCI-AML5 (INCB/Control)
-log10 p-value
5000
4000
3000
2000
1000
0
CTCF
FOSL1
PU.1
ETS1
ERG
FLI1
RUNX1
IRF8
CEBPβ
c-Myc
EGR2
Ranked Order of Transcription Factor Motifs in ATAC Peaks
D
Myeloid lineage Transcription Factor Motifs enriched in OCI-AML5 INCB-treated cells
TF Motif
Sequence of Motif
(-log10) p-value
CTCF
4628.55
FOSL1
4449.04
PU.1
3167.69
FLI1
2372.66
RUNX1
1045.19
IRF8
957.93
CEBPB
532.96
MYC
32.33
EGR2
28.91
E
OCI-AML5 INCB/Control
GFI1 target genes
ABCF2
JMJD1C
SLC44A1
ADD3
GADD45A
CREB5
PDE4D
MBNL1
WDR81
PRDM10
0.0
0.5
1.0
1.5
2.0
Log2 fold-change in ATAC peaks (TSS ± 3 kb/Gene body ± 10 kb)
F
OCI-AML5 INCB/Control
PU.1 target genes
EBF1
LMO2
MME
MEF2C
ITGB2
CSF2RB
CTSK
CD80
GPR132
CXCL9
OPRM1
TNFRSF11A
FGL2
CD79A
CYBB
LYZ
IL1B
LY96
TNFSF10
NR3C1
PARG
MCM3AP
CLEC5A
NF1
JUNB
CIITA
IL12B
CD86
MSR1
PTPRC
0
1
2
3
Log2 Fold-change in ATAC-Seq peaks (TSS ± 3 kb/Gene body ± 10 kb)

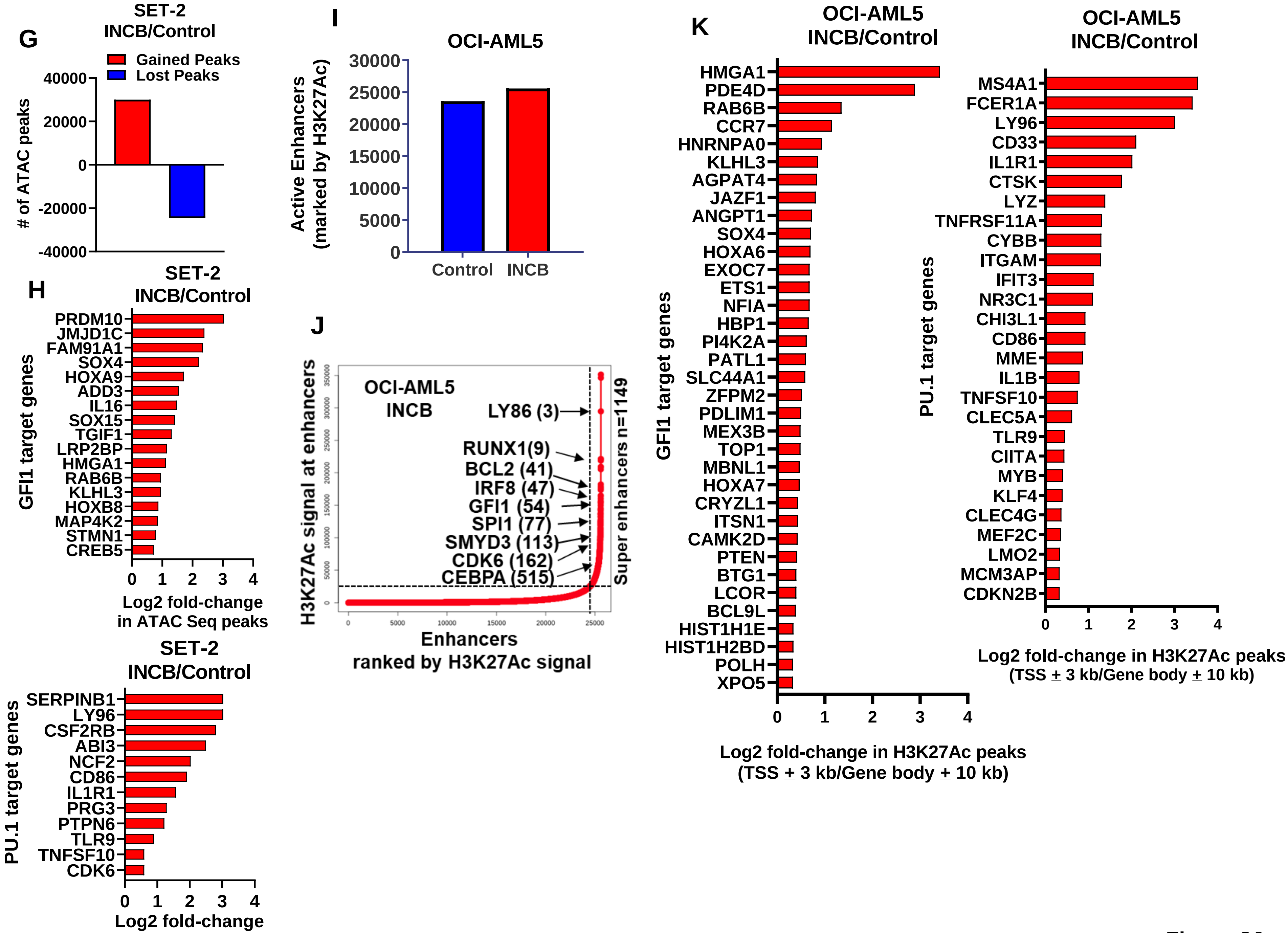
G
SET-2
INCB/Control
Gained Peaks
Lost Peaks
# of ATAC peaks
40000
20000
0
-20000
-40000
I
OCI-AML5
Active Enhancers
(marked by H3K27Ac)
30000
25000
20000
15000
10000
5000
0
Control
INCB
H
SET-2
INCB/Control
GFI1 target genes
PRDM10
JMJD1C
FAM91A1
SOX4
HOXA9
ADD3
IL16
SOX15
TGIF1
LRP2BP
HMGA1
RAB6B
KLHL3
HOXB8
MAP4K2
STMN1
CREB5
0
1
2
3
4
Log2 fold-change
in ATAC Seq peaks
J
OCI-AML5
INCB
H3K27Ac signal at enhancers
LY86 (3)
RUNX1(9)
BCL2 (41)
IRF8 (47)
GFI1 (54)
SPI1 (77)
SMYD3 (113)
CDK6 (162)
CEBPA (515)
Super enhancers n=1149
Enhancers
ranked by H3K27Ac signal
SET-2
INCB/Control
PU.1 target genes
SERPINB1
LY96
CSF2RB
ABI3
NCF2
CD86
IL1R1
PRG3
PTPN6
TLR9
TNFSF10
CDK6
0
1
2
3
4
Log2 fold-change
in ATAC Seq peaks
K
OCI-AML5
INCB/Control
GFI1 target genes
HMGA1
PDE4D
RAB6B
CCR7
HNRNPA0
KLHL3
AGPAT4
JAZF1
ANGPT1
SOX4
HOXA6
EXOC7
ETS1
NFIA
HBP1
PI4K2A
PATL1
SLC44A1
ZFPM2
PDLIM1
MEX3B
TOP1
MBNL1
HOXA7
CRYZL1
ITSN1
CAMK2D
PTEN
BTG1
LCOR
BCL9L
HIST1H1E
HIST1H2BD
POLH
XPO5
0
1
2
3
4
Log2 fold-change in H3K27Ac peaks
(TSS ± 3 kb/Gene body ± 10 kb)
OCI-AML5
INCB/Control
PU.1 target genes
MS4A1
FCER1A
LY96
CD33
IL1R1
CTSK
LYZ
TNFRSF11A
CYBB
ITGAM
IFIT3
NR3C1
CHI3L1
CD86
MME
IL1B
TNFSF10
CLEC5A
TLR9
CIITA
MYB
KLF4
CLEC4G
MEF2C
LMO2
MCM3AP
CDKN2B
0
1
2
3
4
Log2 fold-change in H3K27Ac peaks
(TSS ± 3 kb/Gene body ± 10 kb)

Figure S8

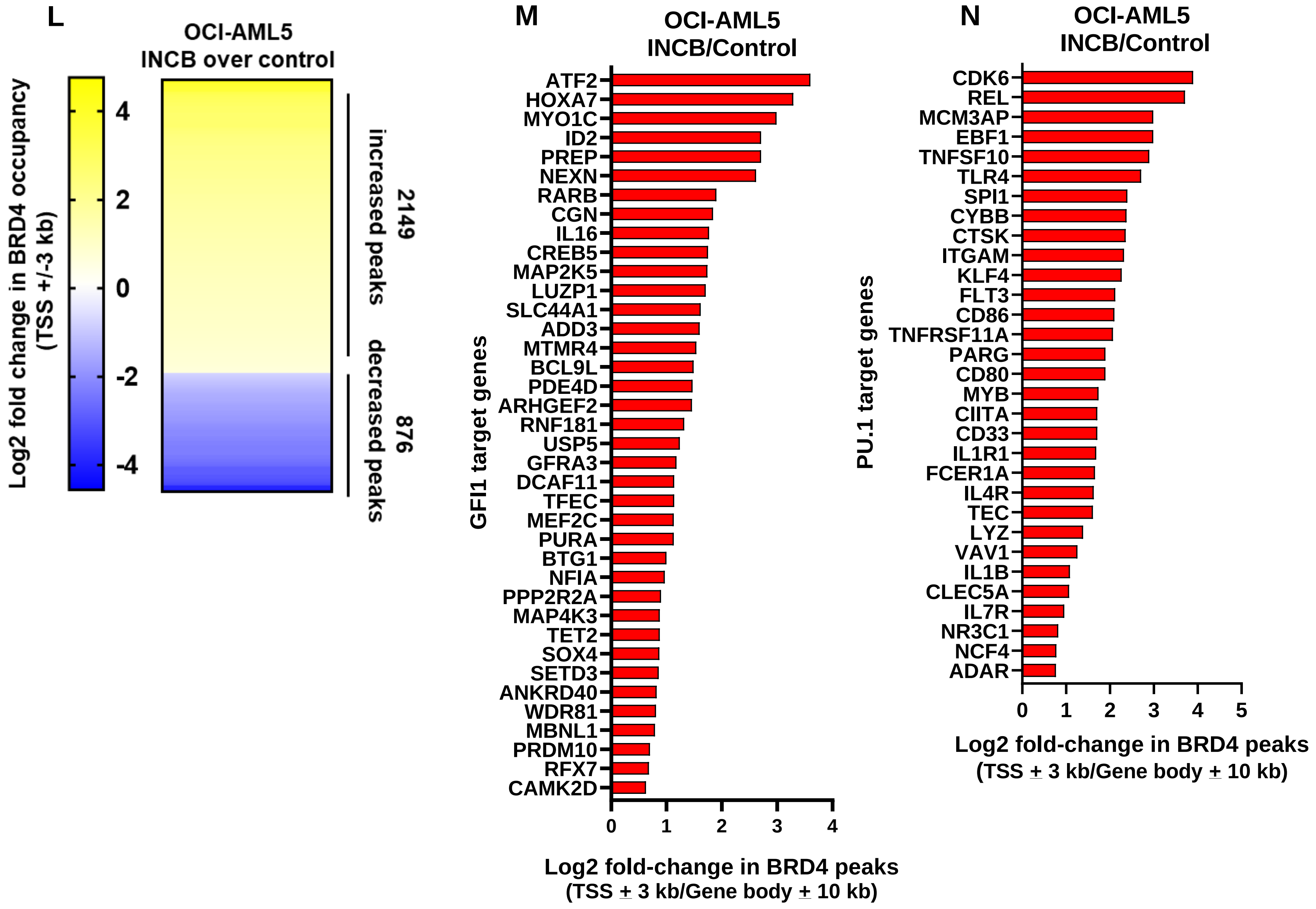

L
OCI-AML5
INCB over control
Log2 fold change in BRD4 occupancy
(TSS +/-3 kb)
4
2
0
-2
-4
increased peaks
2149
decreased peaks
876
M
OCI-AML5
INCB/Control
GFI1 target genes
ATF2
HOXA7
MYO1C
ID2
PREP
NEXN
RARB
CGN
IL16
CREB5
MAP2K5
LUZP1
SLC44A1
ADD3
MTMR4
BCL9L
PDE4D
ARHGEF2
RNF181
USP5
GFRA3
DCAF11
TFEC
MEF2C
PURA
BTG1
NFIA
PPP2R2A
MAP4K3
TET2
SOX4
SETD3
ANKRD40
WDR81
MBNL1
PRDM10
RFX7
CAMK2D
0
1
2
3
4
Log2 fold-change in BRD4 peaks
(TSS + 3 kb/Gene body + 10 kb)
N
OCI-AML5
INCB/Control
PU.1 target genes
CDK6
REL
MCM3AP
EBF1
TNFSF10
TLR4
SPI1
CYBB
CTSK
ITGAM
KLF4
FLT3
CD86
TNFRSF11A
PARG
CD80
MYB
CIITA
CD33
IL1R1
FCER1A
IL4R
TEC
LYZ
VAV1
IL1B
CLEC5A
IL7R
NR3C1
NCF4
ADAR
0
1
2
3
4
5
Log2 fold-change in BRD4 peaks
(TSS + 3 kb/Gene body + 10 kb)

Figure S8

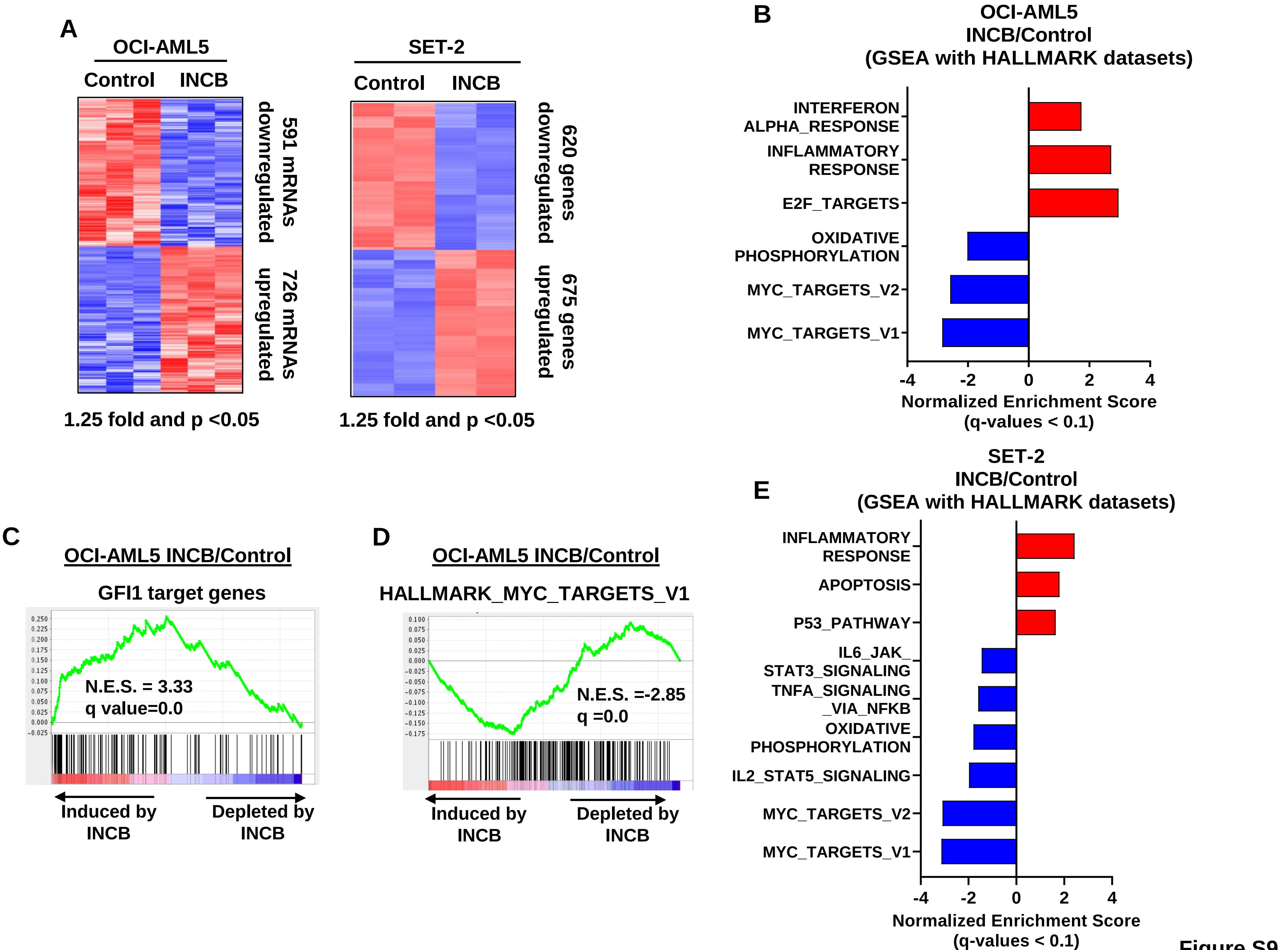

A
OCI-AML5
Control
INCB
591 mRNAs downregulated
726 mRNAs upregulated
1.25 fold and p <0.05
SET-2
Control
INCB
620 genes downregulated
675 genes upregulated
1.25 fold and p <0.05
B
OCI-AML5
INCB/Control
(GSEA with HALLMARK datasets)
INTERFERON ALPHA_RESPONSE
INFLAMMATORY RESPONSE
E2F_TARGETS
OXIDATIVE PHOSPHORYLATION
MYC_TARGETS_V2
MYC_TARGETS_V1
-4
-2
0
2
4
Normalized Enrichment Score
(q-values < 0.1)
C
OCI-AML5 INCB/Control
GFI1 target genes
N.E.S. = 3.33
q value=0.0
Induced by INCB
Depleted by INCB
D
OCI-AML5 INCB/Control
HALLMARK_MYC_TARGETS_V1
N.E.S. =-2.85
q =0.0
Induced by INCB
Depleted by INCB
E
SET-2
INCB/Control
(GSEA with HALLMARK datasets)
INFLAMMATORY RESPONSE
APOPTOSIS
P53_PATHWAY
IL6_JAK_ STAT3_SIGNALING
TNFA_SIGNALING _VIA_NFKB
OXIDATIVE PHOSPHORYLATION
IL2_STAT5_SIGNALING
MYC_TARGETS_V2
MYC_TARGETS_V1
-4
-2
0
2
4
Normalized Enrichment Score
(q-values < 0.1)

Figure S9

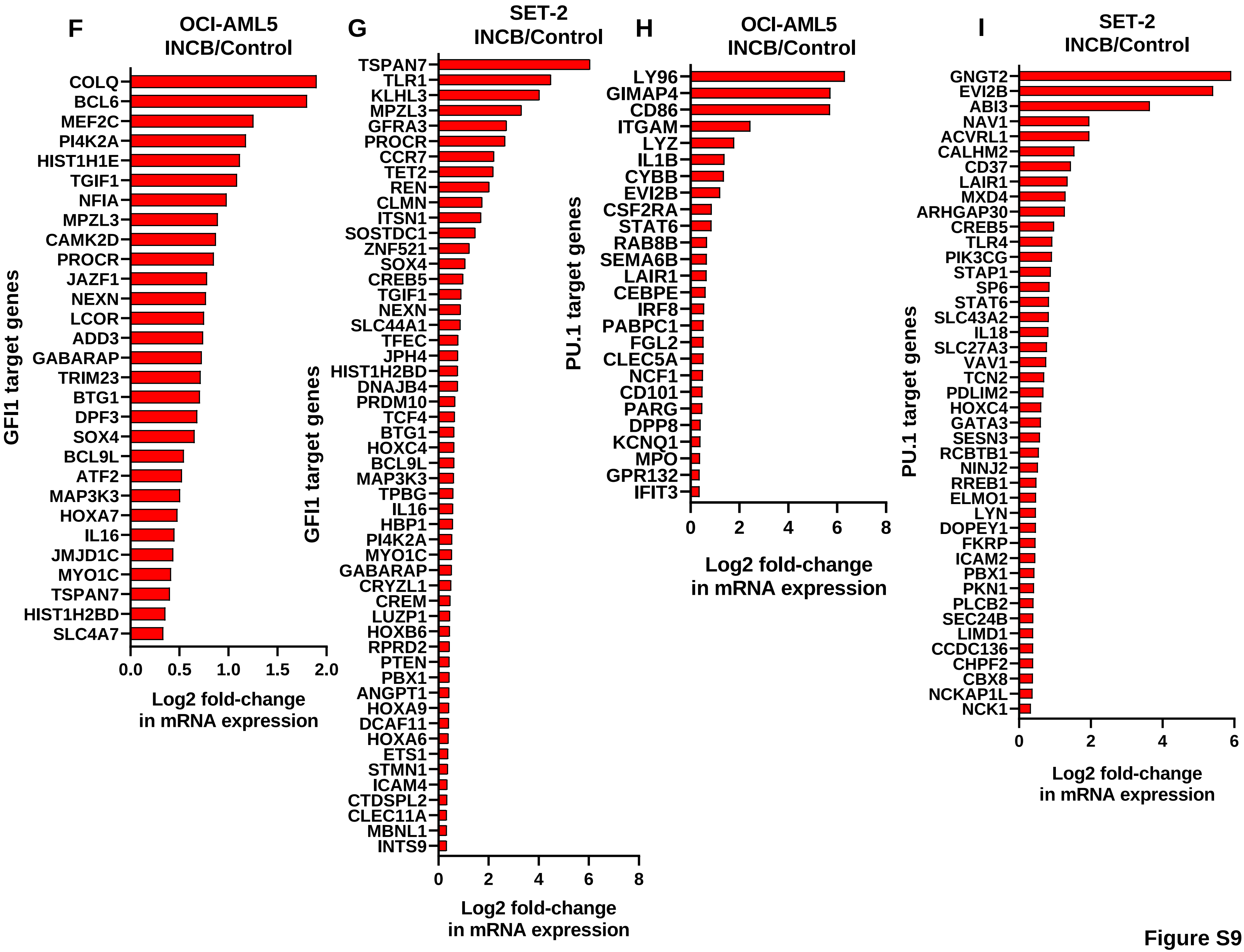

F
OCI-AML5
INCB/Control
GFI1 target genes
COLQ
BCL6
MEF2C
PI4K2A
HIST1H1E
TGIF1
NFIA
MPZL3
CAMK2D
PROCR
JAZF1
NEXN
LCOR
ADD3
GABARAP
TRIM23
BTG1
DPF3
SOX4
BCL9L
ATF2
MAP3K3
HOXA7
IL16
JMJD1C
MYO1C
TSPAN7
HIST1H2BD
SLC4A7
0.0
0.5
1.0
1.5
2.0
Log2 fold-change
in mRNA expression
G
SET-2
INCB/Control
GFI1 target genes
TSPAN7
TLR1
KLHL3
MPZL3
GFRA3
PROCR
CCR7
TET2
REN
CLMN
ITSN1
SOSTDC1
ZNF521
SOX4
CREB5
TGIF1
NEXN
SLC44A1
TFEC
JPH4
HIST1H2BD
DNAJB4
PRDM10
TCF4
BTG1
HOXC4
BCL9L
MAP3K3
TPBG
IL16
HBP1
PI4K2A
MYO1C
GABARAP
CRYZL1
CREM
LUZP1
HOXB6
RPRD2
PTEN
PBX1
ANGPT1
HOXA9
DCAF11
HOXA6
ETS1
STMN1
ICAM4
CTDSPL2
CLEC11A
MBNL1
INTS9
0
2
4
6
8
Log2 fold-change
in mRNA expression
H
OCI-AML5
INCB/Control
PU.1 target genes
LY96
GIMAP4
CD86
ITGAM
LYZ
IL1B
CYBB
EVI2B
CSF2RA
STAT6
RAB8B
SEMA6B
LAIR1
CEBPE
IRF8
PABPC1
FGL2
CLEC5A
NCF1
CD101
PARG
DPP8
KCNQ1
MPO
GPR132
IFIT3
0
2
4
6
8
Log2 fold-change
in mRNA expression
I
SET-2
INCB/Control
PU.1 target genes
GNGT2
EVI2B
ABI3
NAV1
ACVRL1
CALHM2
CD37
LAIR1
MXD4
ARHGAP30
CREB5
TLR4
PIK3CG
STAP1
SP6
STAT6
SLC43A2
IL18
SLC27A3
VAV1
TCN2
PDLIM2
HOXC4
GATA3
SESN3
RCBTB1
NINJ2
RREB1
ELMO1
LYN
DOPEY1
FKRP
ICAM2
PBX1
PKN1
PLCB2
SEC24B
LIMD1
CCDC136
CHPF2
CBX8
NCKAP1L
NCK1
0
2
4
6
Log2 fold-change
in mRNA expression

Figure S9

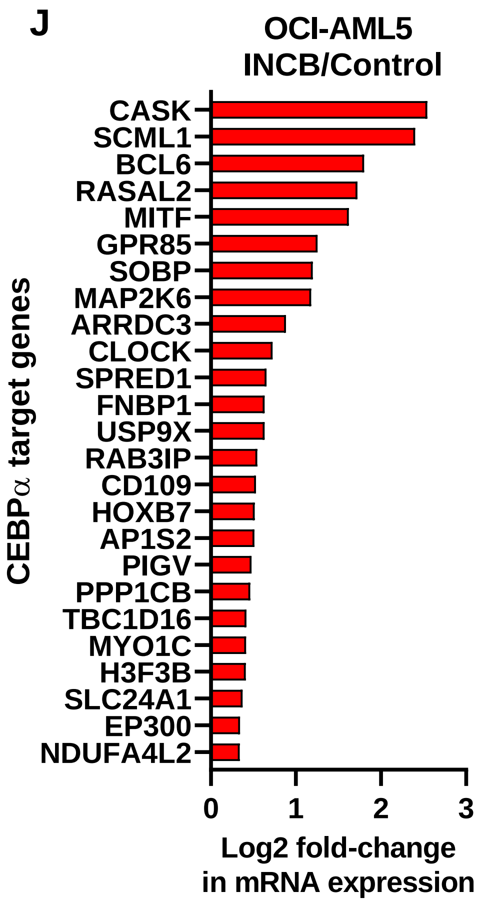
J
OCI-AML5
INCB/Control
CEBPα target genes
CASK
SCML1
BCL6
RASAL2
MITF
GPR85
SOBP
MAP2K6
ARRDC3
CLOCK
SPRED1
FNBP1
USP9X
RAB3IP
CD109
HOXB7
AP1S2
PIGV
PPP1CB
TBC1D16
MYO1C
H3F3B
SLC24A1
EP300
NDUFA4L2
0
1
2
3
Log2 fold-change
in mRNA expression

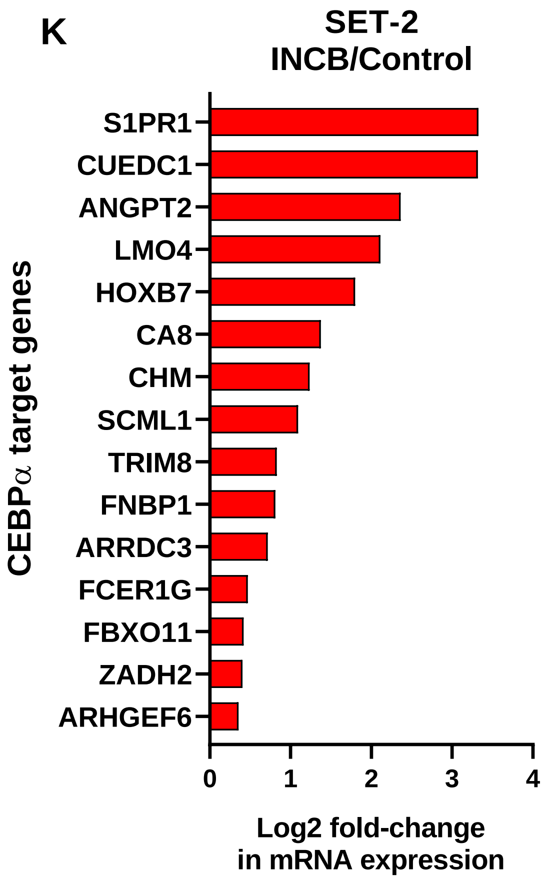
K
SET-2
INCB/Control
CEBPα target genes
S1PR1
CUEDC1
ANGPT2
LMO4
HOXB7
CA8
CHM
SCML1
TRIM8
FNBP1
ARRDC3
FCER1G
FBXO11
ZADH2
ARHGEF6
0
1
2
3
4
Log2 fold-change
in mRNA expression

Figure S9

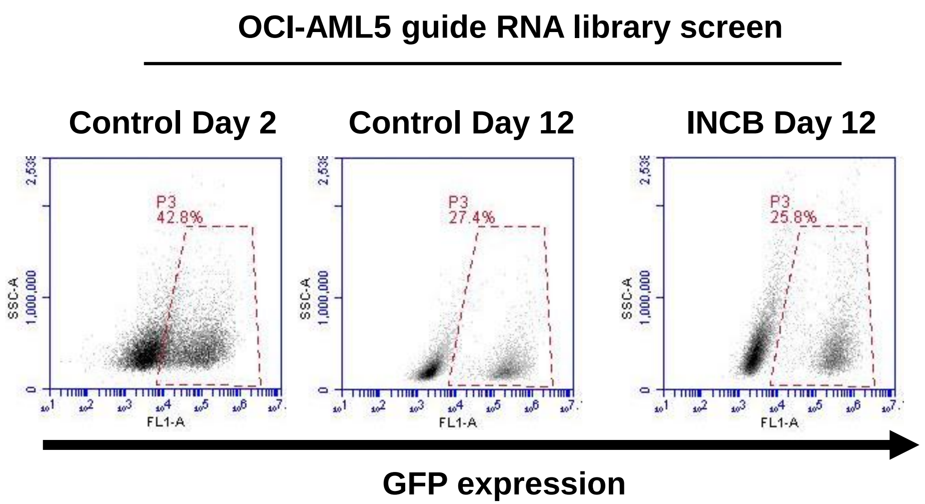
OCI-AML5 guide RNA library screen
Control Day 2
Control Day 12
INCB Day 12
P3
42.8%
P3
27.4%
P3
25.8%
SSC-A
FL1-A
GFP expression

Figure S10

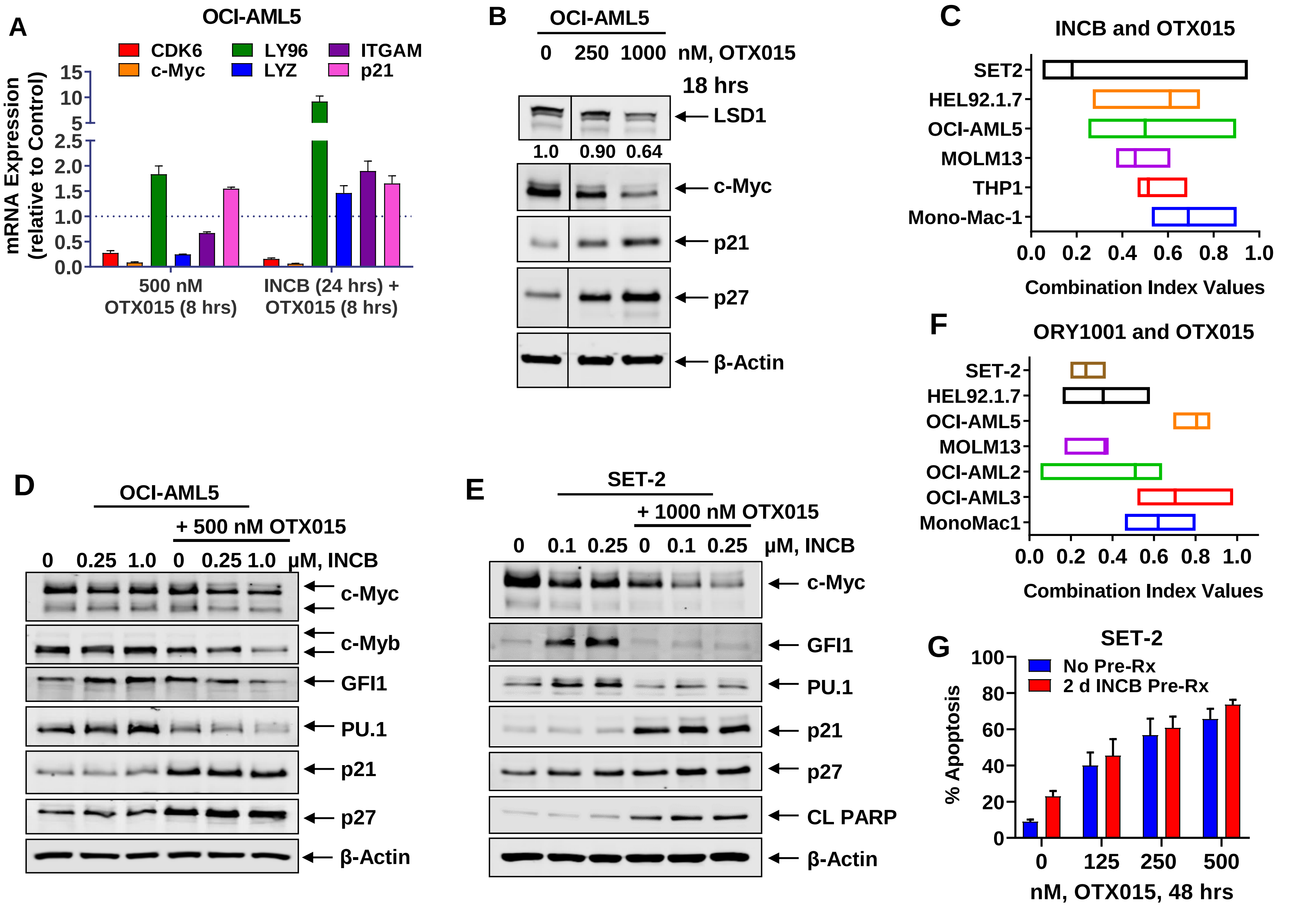
A
OCI-AML5
CDK6
c-Myc
LY96
LYZ
ITGAM
p21
mRNA Expression
(relative to Control)
500 nM
OTX015 (8 hrs)
INCB (24 hrs) +
OTX015 (8 hrs)
B
OCI-AML5
0 250 1000 nM, OTX015
18 hrs
LSD1
1.0 0.90 0.64
c-Myc
p21
p27
β-Actin
C
INCB and OTX015
SET2
HEL92.1.7
OCI-AML5
MOLM13
THP1
Mono-Mac-1
Combination Index Values
D
OCI-AML5
+ 500 nM OTX015
0 0.25 1.0 0 0.25 1.0 μM, INCB
c-Myc
c-Myb
GFI1
PU.1
p21
p27
β-Actin
E
SET-2
+ 1000 nM OTX015
0 0.1 0.25 0 0.1 0.25 μM, INCB
c-Myc
GFI1
PU.1
p21
p27
CL PARP
β-Actin
F
ORY1001 and OTX015
SET-2
HEL92.1.7
OCI-AML5
MOLM13
OCI-AML2
OCI-AML3
MonoMac1
Combination Index Values
G
SET-2
No Pre-Rx
2 d INCB Pre-Rx
% Apoptosis
nM, OTX015, 48 hrs

Figure S11

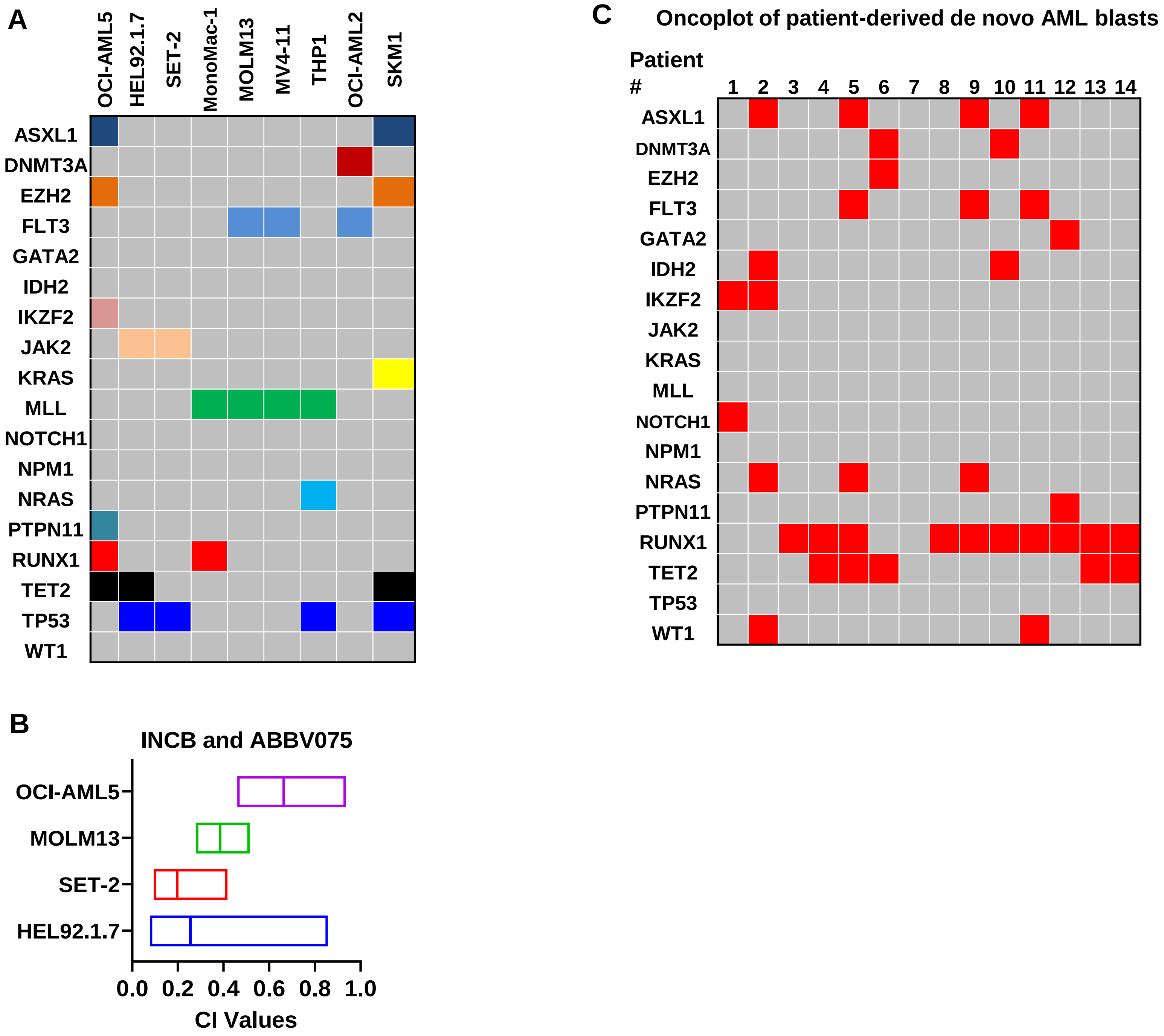
A
OCI-AML5
HEL92.1.7
SET-2
MonoMac-1
MOLM13
MV4-11
THP1
OCI-AML2
SKM1
ASXL1
DNMT3A
EZH2
FLT3
GATA2
IDH2
IKZF2
JAK2
KRAS
MLL
NOTCH1
NPM1
NRAS
PTPN11
RUNX1
TET2
TP53
WT1
B
INCB and ABBV075
OCI-AML5
MOLM13
SET-2
HEL92.1.7
0.0 0.2 0.4 0.6 0.8 1.0
CI Values
C
Oncoplot of patient-derived de novo AML blasts
Patient #
1 2 3 4 5 6 7 8 9 10 11 12 13 14
ASXL1
DNMT3A
EZH2
FLT3
GATA2
IDH2
IKZF2
JAK2
KRAS
MLL
NOTCH1
NPM1
NRAS
PTPN11
RUNX1
TET2
TP53
WT1

Figure S12

D

Oncoplot of patient-derived post-MPN sAML blasts

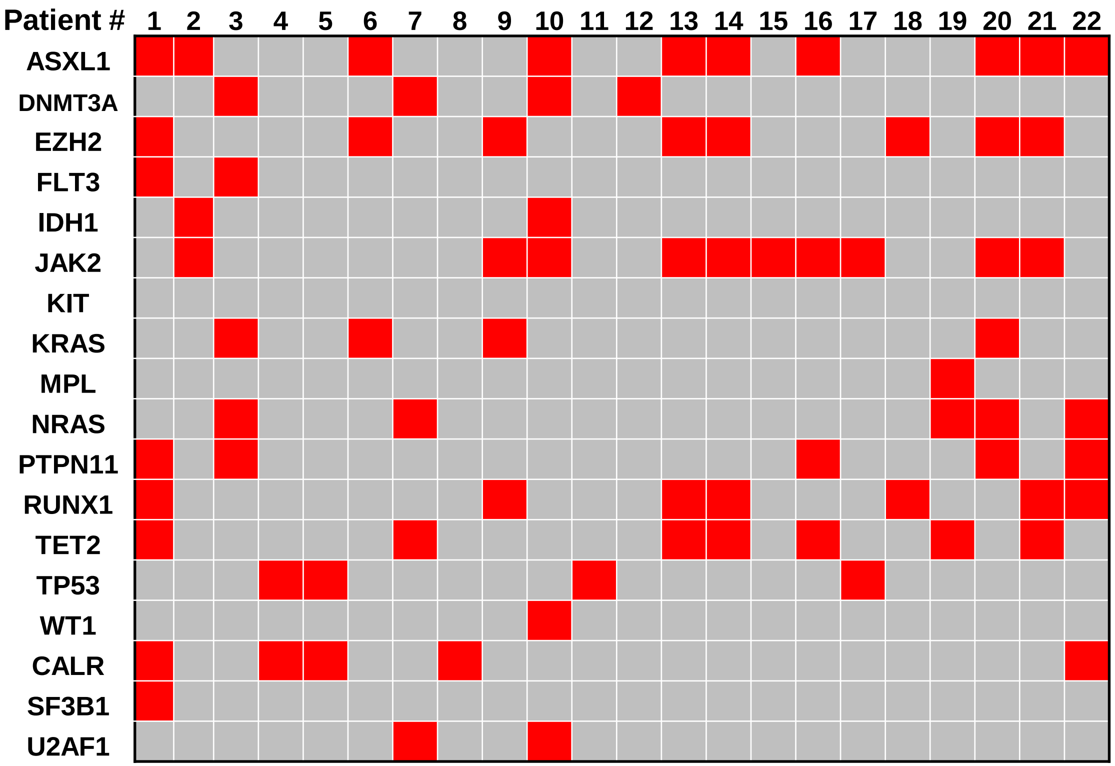

Figure S12

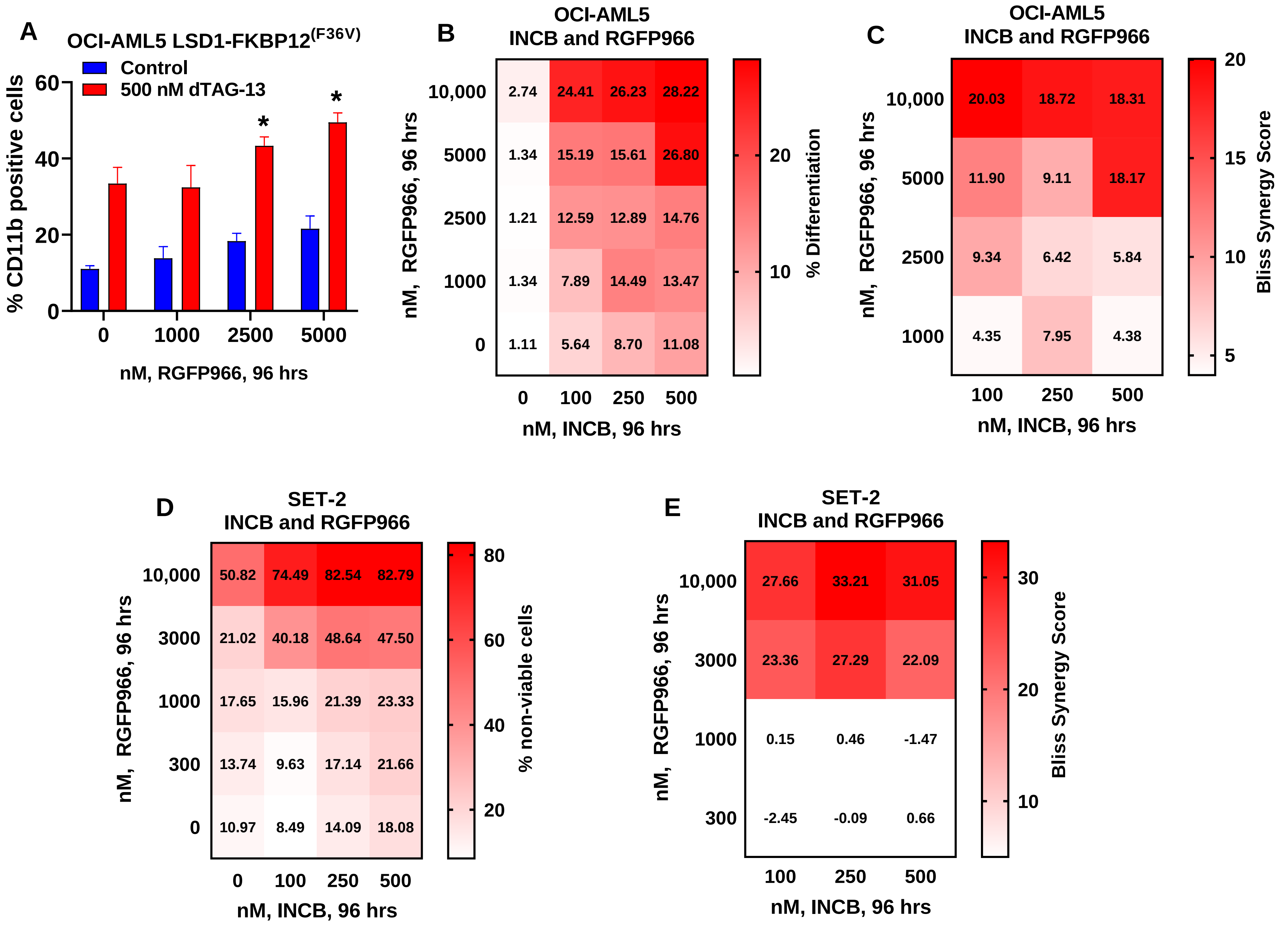
A
OCI-AML5 LSD1-FKBP12(F36V)
Control
500 nM dTAG-13
% CD11b positive cells
60
40
20
0
*
*
0
1000
2500
5000
nM, RGFP966, 96 hrs
B
OCI-AML5
INCB and RGFP966
nM, RGFP966, 96 hrs
10,000
5000
2500
1000
0
2.74 24.41 26.23 28.22
1.34 15.19 15.61 26.80
1.21 12.59 12.89 14.76
1.34 7.89 14.49 13.47
1.11 5.64 8.70 11.08
0
100
250
500
nM, INCB, 96 hrs
% Differentiation
20
10
C
OCI-AML5
INCB and RGFP966
nM, RGFP966, 96 hrs
10,000
5000
2500
1000
20.03 18.72 18.31
11.90 9.11 18.17
9.34 6.42 5.84
4.35 7.95 4.38
100
250
500
nM, INCB, 96 hrs
Bliss Synergy Score
20
15
10
5
D
SET-2
INCB and RGFP966
nM, RGFP966, 96 hrs
10,000
3000
1000
300
0
50.82 74.49 82.54 82.79
21.02 40.18 48.64 47.50
17.65 15.96 21.39 23.33
13.74 9.63 17.14 21.66
10.97 8.49 14.09 18.08
0
100
250
500
nM, INCB, 96 hrs
% non-viable cells
80
60
40
20
E
SET-2
INCB and RGFP966
nM, RGFP966, 96 hrs
10,000
3000
1000
300
27.66 33.21 31.05
23.36 27.29 22.09
0.15 0.46 -1.47
-2.45 -0.09 0.66
100
250
500
nM, INCB, 96 hrs
Bliss Synergy Score
30
20
10

Figure S13

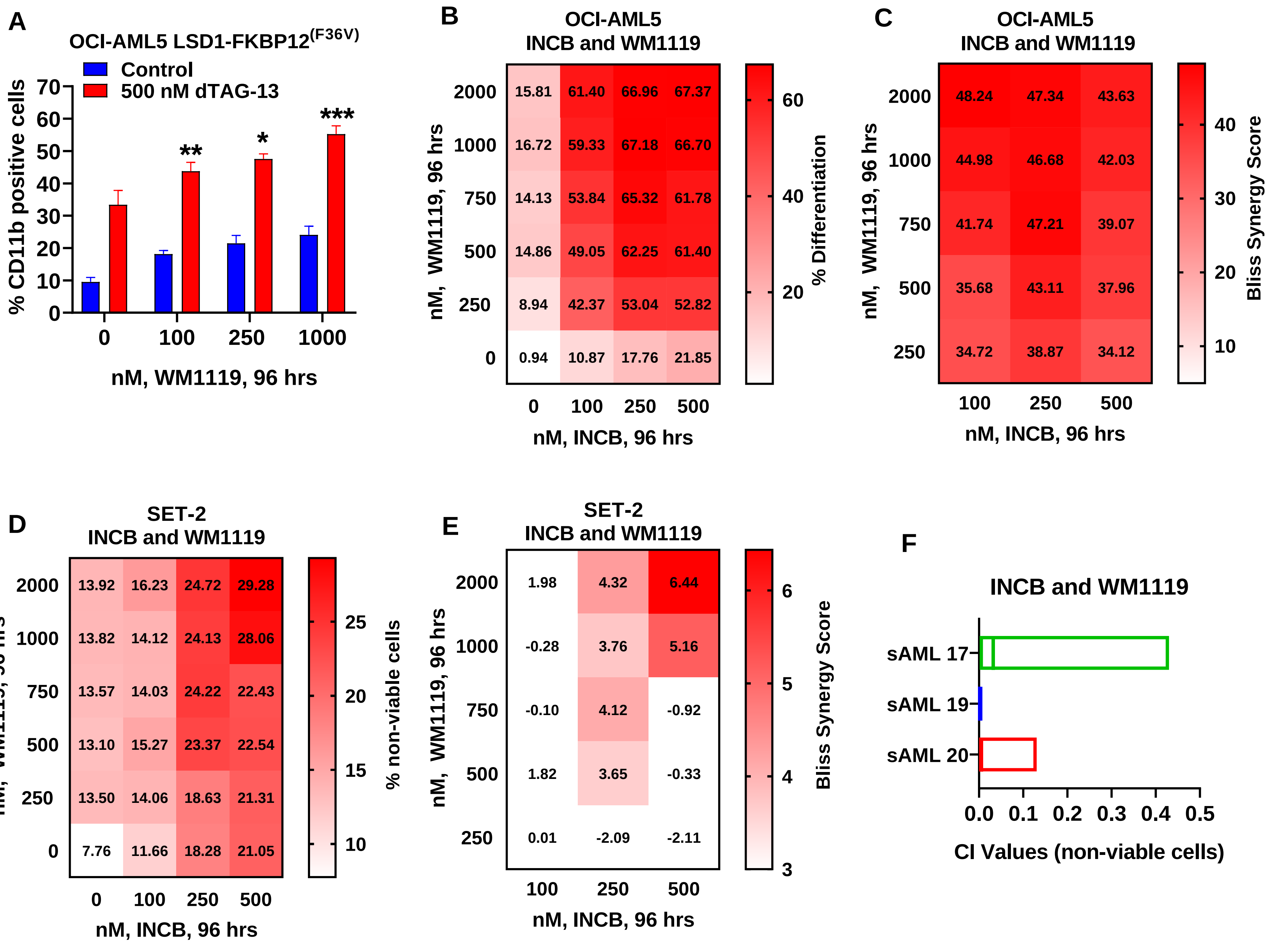
A
OCI-AML5 LSD1-FKBP12(F36V)
Control
500 nM dTAG-13
% CD11b positive cells
0
10
20
30
40
50
60
70
**
*
***
0
100
250
1000
nM, WM1119, 96 hrs
B
OCI-AML5
INCB and WM1119
2000
1000
750
500
250
0
15.81 61.40 66.96 67.37
16.72 59.33 67.18 66.70
14.13 53.84 65.32 61.78
14.86 49.05 62.25 61.40
8.94 42.37 53.04 52.82
0.94 10.87 17.76 21.85
nM, WM1119, 96 hrs
0
100
250
500
nM, INCB, 96 hrs
60
40
20
% Differentiation
C
OCI-AML5
INCB and WM1119
2000
1000
750
500
250
48.24 47.34 43.63
44.98 46.68 42.03
41.74 47.21 39.07
35.68 43.11 37.96
34.72 38.87 34.12
nM, WM1119, 96 hrs
100
250
500
nM, INCB, 96 hrs
40
30
20
10
Bliss Synergy Score
D
SET-2
INCB and WM1119
2000
1000
750
500
250
0
13.92 16.23 24.72 29.28
13.82 14.12 24.13 28.06
13.57 14.03 24.22 22.43
13.10 15.27 23.37 22.54
13.50 14.06 18.63 21.31
7.76 11.66 18.28 21.05
nM, WM1119, 96 hrs
0
100
250
500
nM, INCB, 96 hrs
25
20
15
10
% non-viable cells
E
SET-2
INCB and WM1119
2000
1000
750
500
250
1.98 4.32 6.44
-0.28 3.76 5.16
-0.10 4.12 -0.92
1.82 3.65 -0.33
0.01 -2.09 -2.11
nM, WM1119, 96 hrs
100
250
500
nM, INCB, 96 hrs
6
5
4
3
Bliss Synergy Score
F
INCB and WM1119
sAML 17
sAML 19
sAML 20
0.0
0.1
0.2
0.3
0.4
0.5
CI Values (non-viable cells)

Figure S14

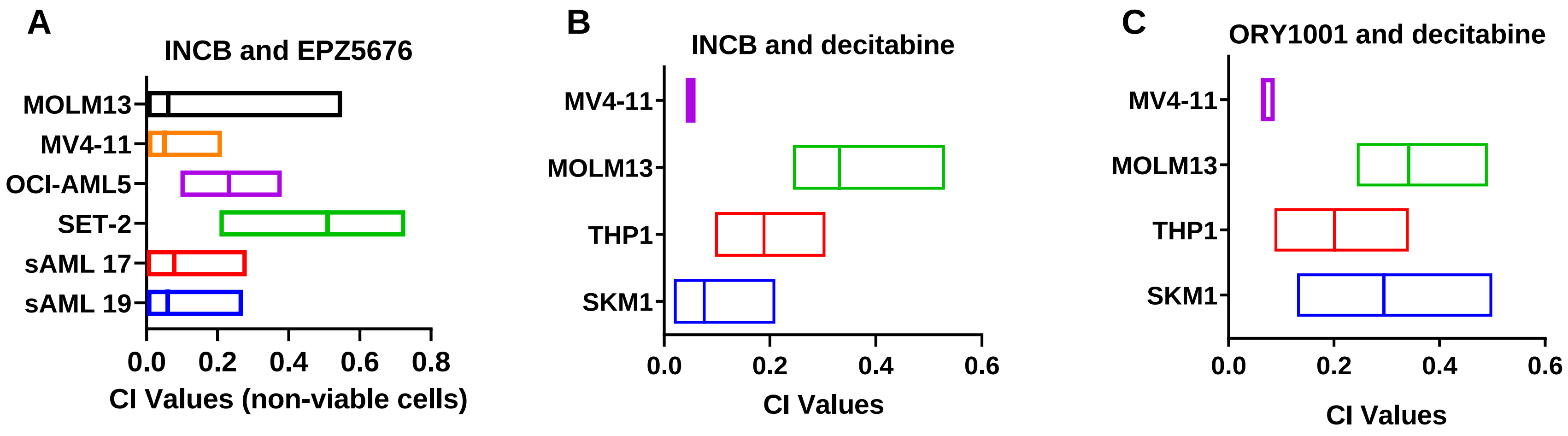

A
INCB and EPZ5676
MOLM13
MV4-11
OCI-AML5
SET-2
sAML 17
sAML 19
0.0
0.2
0.4
0.6
0.8
CI Values (non-viable cells)
B
INCB and decitabine
MV4-11
MOLM13
THP1
SKM1
0.0
0.2
0.4
0.6
CI Values
C
ORY1001 and decitabine
MV4-11
MOLM13
THP1
SKM1
0.0
0.2
0.4
0.6
CI Values

Figure S15

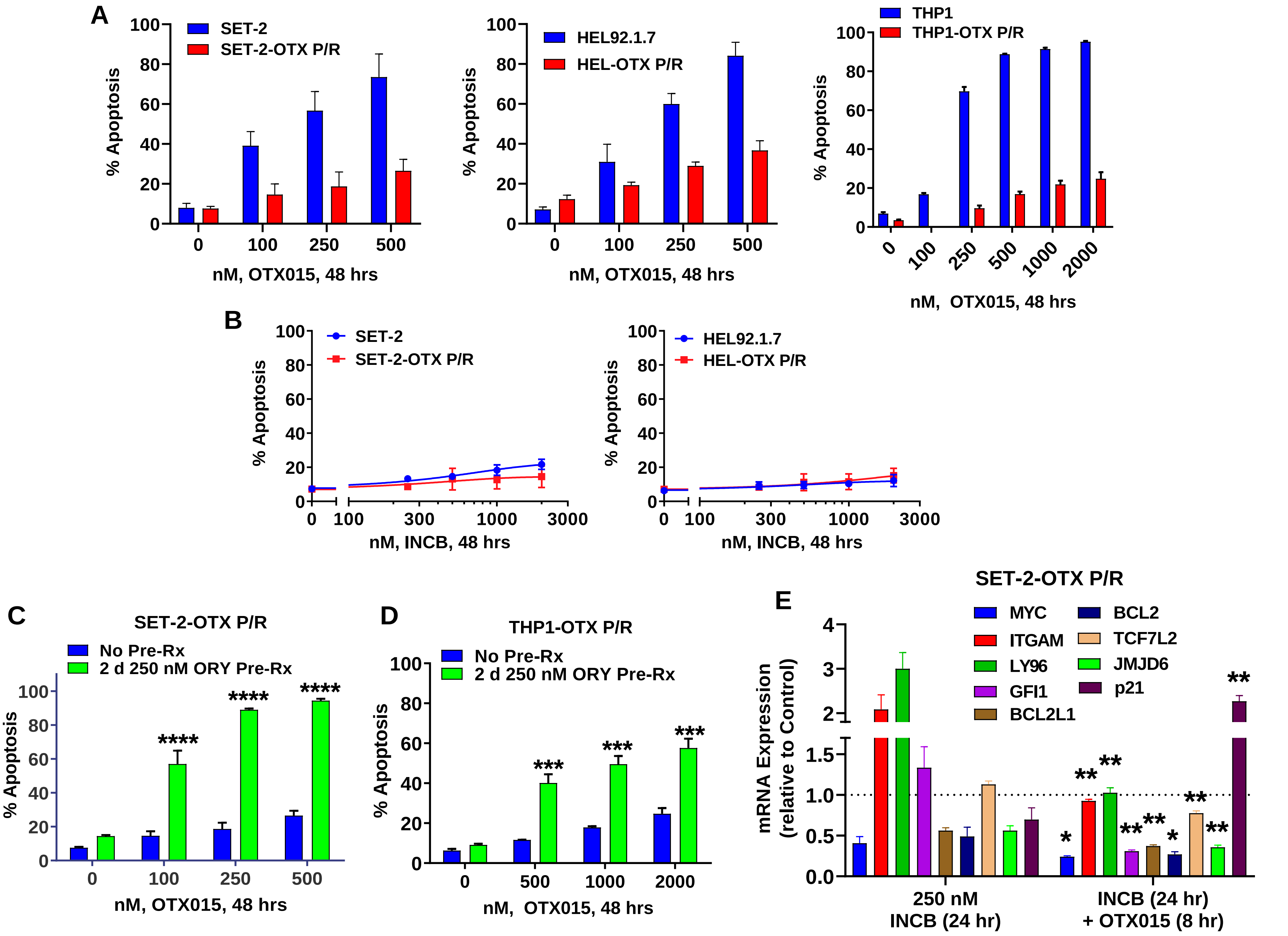
A
% Apoptosis
SET-2
SET-2-OTX P/R
nM, OTX015, 48 hrs
HEL92.1.7
HEL-OTX P/R
THP1
THP1-OTX P/R
B
nM, INCB, 48 hrs
C
SET-2-OTX P/R
No Pre-Rx
2 d 250 nM ORY Pre-Rx
D
THP1-OTX P/R
E
SET-2-OTX P/R
mRNA Expression
(relative to Control)
MYC
ITGAM
LY96
GFI1
BCL2L1
BCL2
TCF7L2
JMJD6
p21
250 nM
INCB (24 hr)
INCB (24 hr)
+ OTX015 (8 hr)

Figure S16

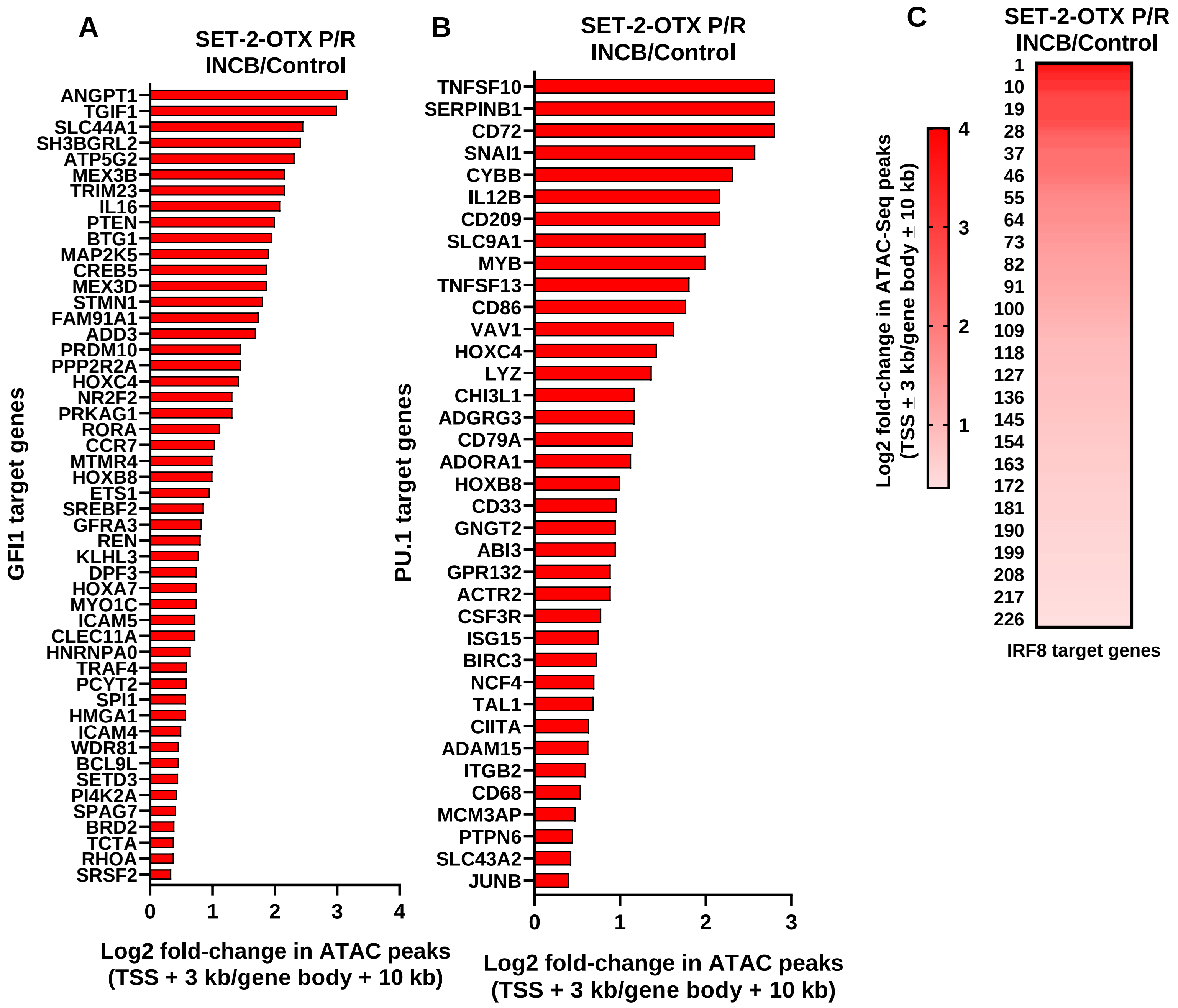
A
SET-2-OTX P/R
INCB/Control
GFI1 target genes
ANGPT1
TGIF1
SLC44A1
SH3BGRL2
ATP5G2
MEX3B
TRIM23
IL16
PTEN
BTG1
MAP2K5
CREB5
MEX3D
STMN1
FAM91A1
ADD3
PRDM10
PPP2R2A
HOXC4
NR2F2
PRKAG1
RORA
CCR7
MTMR4
HOXB8
ETS1
SREBF2
GFRA3
REN
KLHL3
DPF3
HOXA7
MYO1C
ICAM5
CLEC11A
HNRNPA0
TRAF4
PCYT2
SPI1
HMGA1
ICAM4
WDR81
BCL9L
SETD3
PI4K2A
SPAG7
BRD2
TCTA
RHOA
SRSF2
0
1
2
3
4
Log2 fold-change in ATAC peaks
(TSS ± 3 kb/gene body ± 10 kb)
B
SET-2-OTX P/R
INCB/Control
PU.1 target genes
TNFSF10
SERPINB1
CD72
SNAI1
CYBB
IL12B
CD209
SLC9A1
MYB
TNFSF13
CD86
VAV1
HOXC4
LYZ
CHI3L1
ADGRG3
CD79A
ADORA1
HOXB8
CD33
GNGT2
ABI3
GPR132
ACTR2
CSF3R
ISG15
BIRC3
NCF4
TAL1
CIITA
ADAM15
ITGB2
CD68
MCM3AP
PTPN6
SLC43A2
JUNB
0
1
2
3
Log2 fold-change in ATAC peaks
(TSS ± 3 kb/gene body ± 10 kb)
C
SET-2-OTX P/R
INCB/Control
Log2 fold-change in ATAC-Seq peaks
(TSS ± 3 kb/gene body ± 10 kb)
4
3
2
1
1
10
19
28
37
46
55
64
73
82
91
100
109
118
127
136
145
154
163
172
181
190
199
208
217
226
IRF8 target genes

Figure S17

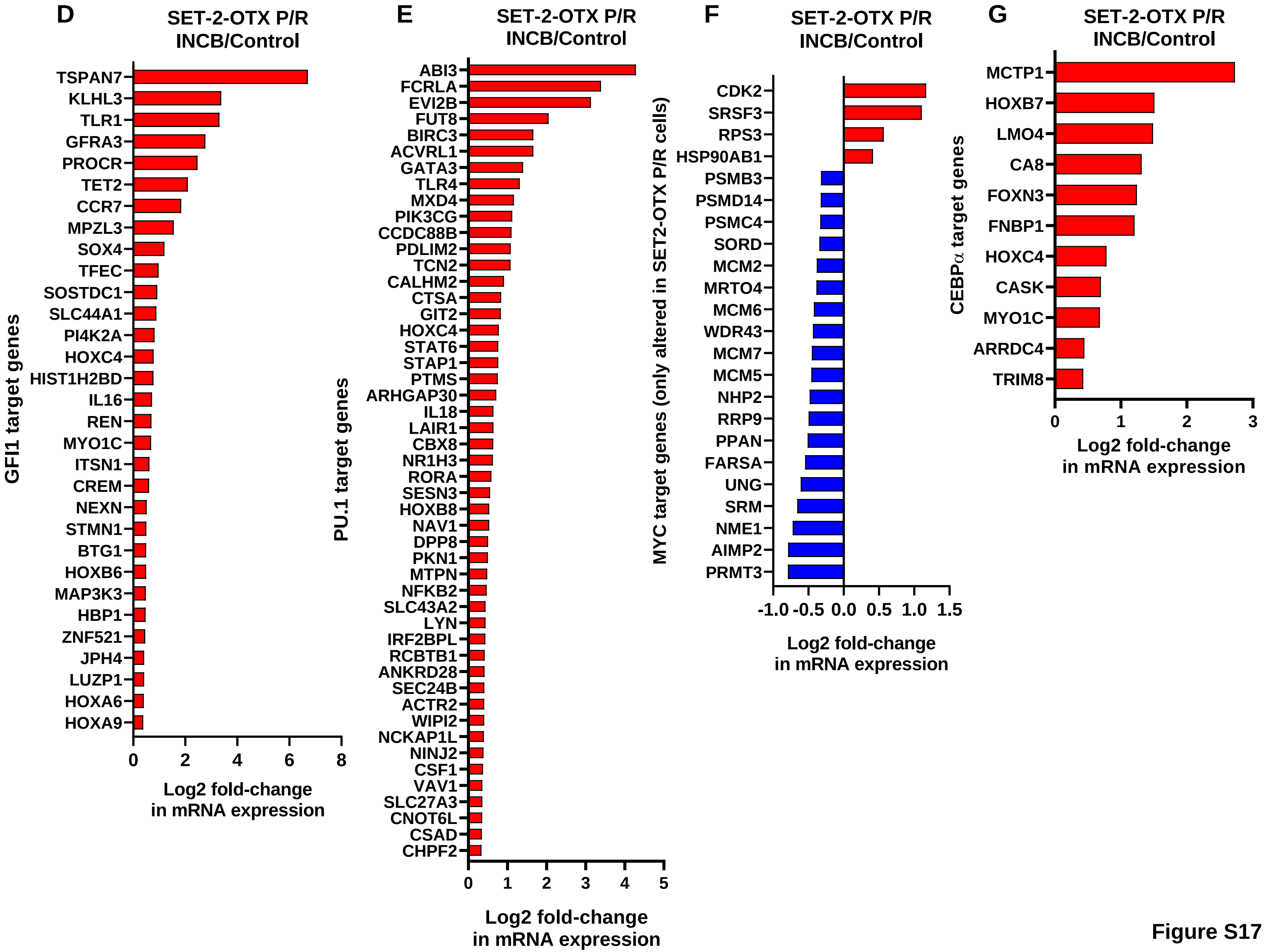
D
SET-2-OTX P/R
INCB/Control
GFI1 target genes
TSPAN7
KLHL3
TLR1
GFRA3
PROCR
TET2
CCR7
MPZL3
SOX4
TFEC
SOSTDC1
SLC44A1
PI4K2A
HOXC4
HIST1H2BD
IL16
REN
MYO1C
ITSN1
CREM
NEXN
STMN1
BTG1
HOXB6
MAP3K3
HBP1
ZNF521
JPH4
LUZP1
HOXA6
HOXA9
0
2
4
6
8
Log2 fold-change
in mRNA expression
E
SET-2-OTX P/R
INCB/Control
PU.1 target genes
ABI3
FCRLA
EVI2B
FUT8
BIRC3
ACVRL1
GATA3
TLR4
MXD4
PIK3CG
CCDC88B
PDLIM2
TCN2
CALHM2
CTSA
GIT2
HOXC4
STAT6
STAP1
PTMS
ARHGAP30
IL18
LAIR1
CBX8
NR1H3
RORA
SESN3
HOXB8
NAV1
DPP8
PKN1
MTPN
NFKB2
SLC43A2
LYN
IRF2BPL
RCBTB1
ANKRD28
SEC24B
ACTR2
WIPI2
NCKAP1L
NINJ2
CSF1
VAV1
SLC27A3
CNOT6L
CSAD
CHPF2
0
1
2
3
4
5
Log2 fold-change
in mRNA expression
F
SET-2-OTX P/R
INCB/Control
MYC target genes (only altered in SET2-OTX P/R cells)
CDK2
SRSF3
RPS3
HSP90AB1
PSMB3
PSMD14
PSMC4
SORD
MCM2
MRTO4
MCM6
WDR43
MCM7
MCM5
NHP2
RRP9
PPAN
FARSA
UNG
SRM
NME1
AIMP2
PRMT3
-1.0
-0.5
0.0
0.5
1.0
1.5
Log2 fold-change
in mRNA expression
G
SET-2-OTX P/R
INCB/Control
CEBPα target genes
MCTP1
HOXB7
LMO4
CA8
FOXN3
FNBP1
HOXC4
CASK
MYO1C
ARRDC4
TRIM8
0
1
2
3
Log2 fold-change
in mRNA expression

Figure S17

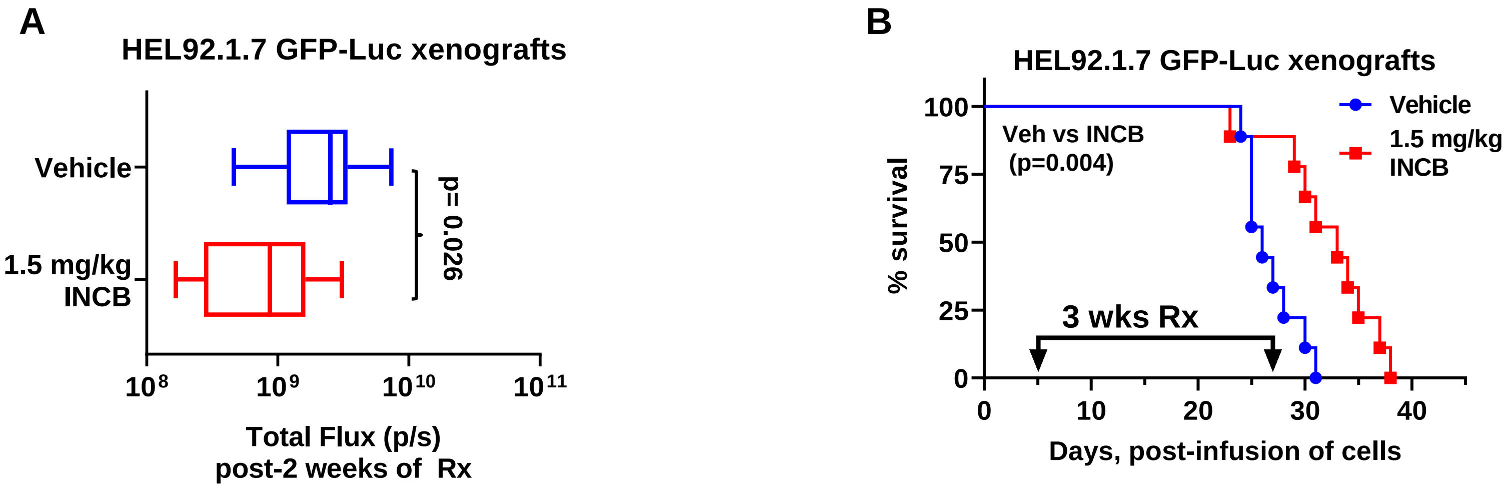
A
HEL92.1.7 GFP-Luc xenografts
Vehicle
1.5 mg/kg INCB
p= 0.026
10^8
10^9
10^{10}
10^{11}
Total Flux (p/s) post-2 weeks of Rx
B
HEL92.1.7 GFP-Luc xenografts
Vehicle
1.5 mg/kg INCB
Veh vs INCB (p=0.004)
% survival
100
75
50
25
0
3 wks Rx
0
10
20
30
40
Days, post-infusion of cells

Figure S18
