# Supplementary material for: Superior efficacy of co-targeting GFI1/KDM1A and BRD4 against AML and post-MPN secondary AML cells
Source: Blood Cancer J. 2021 May 20;11(5):98. doi: 10.1038/s41408-021-00487-3 (PMC8138012; doi:10.1038/s41408-021-00487-3)
Supplement: Supplementary file 4 — Supplemental Materials and Methods [file 41408_2021_487_MOESM4_ESM.docx]

**Supplemental Methods:**

**Contact for Reagent sharing. Kapil N Bhalla. Department of Leukemia, MD. Anderson Cancer Center, 1400 Holcombe Blvd, Unit428, Houston, TX, 77030. kbhalla@mdanderson.org**

**Reagents and antibodies.** INCB059872 and ruxolitinib were obtained from Incyte Corporation (Wilmington, DE) under a material transfer agreement. ORY-1001 was obtained from Cayman Chemicals (Ann Arbor, Michigan). OTX015 (BET inhibitor), WM1119 (MOZ inhibitor), EPZ5676 (DOT1L inhibitor), and RGFP966 (HDAC3-specific inhibitor) were obtained from MedChem Express (Monmouth Junction, NJ). Decitabine was obtained from Sigma-Aldrich (St. Louis, MO). AC220 and ABBV075 were obtained from Selleck Chemicals (Houston, TX). All compounds were prepared as 10 mM stocks in 100% DMSO and frozen at -80°C in 5-10 µL aliquots to allow for single use, thus avoiding multiple freeze-thaw cycles that could result in compound decomposition and loss of activity. Anti-BRD4 [RRID: AB_1576498], anti-BRD2 [RRID: AB_2034828], and anti-pSer2 RNAP2 [RRID: AB_2631452] antibodies were obtained from Bethyl Labs (Montgomery, TX). Anti-c-Myc [RRID:AB_1903938], anti-c-Myb [RRID:AB_2716637], anti-PU.1 [RRID:AB_10693421], anti-MCL1 [RRID:AB_2799149], anti-HEXIM1 [RRID:AB_2797969], anti-p21 [RRID:AB_823586], and anti-Bcl-xL [RRID:AB_10695729] antibodies were obtained from Cell Signaling Technologies (Beverly, MA). Anti-CDK4 [RRID:AB_631221], anti-CDK6 [RRID:AB_10610066], anti-BCL2 [RRID:AB_626733], anti-TCF7L2 [RRID:AB_2199823], anti-JMJD6 [RRID:AB_628185], and anti-β-Actin [RRID:AB_626630] antibodies were obtained from Santa Cruz Biotechnologies (Santa Cruz, CA).

**Cell lines and cell culture.** OCI-AML5 [DSMZ Cat# ACC-247, RRID:CVCL_1620], OCI-AML2 [DSMZ Cat# ACC-99, RRID:CVCL_1619], Mono-Mac-1 [DSMZ Cat# ACC-252, RRID:CVCL_1425], MOLM13 [DSMZ Cat# ACC-554, RRID:CVCL_2119], SKM-1 [DSMZ Cat# ACC-547, RRID:CVCL_0098], and SET-2 cells [DSMZ Cat# ACC-608, RRID:CVCL_2187] cells were obtained from the DSMZ. MV4-11 [ATCC Cat# CRL-9591, RRID:CVCL_0064], HEL92.1.7 [ATCC Cat# TIB-180, RRID: CVCL_2481], THP1 [ATCC Cat# TIB-202, RRID:CVCL_0006], and HS5 [ATCC Cat# CRL-11882, RRID:CVCL_3720] cells were obtained from the ATCC (Manassas, VA). HEK-293T cells were obtained from the Characterized Cell Line Core Facility at M.D. Anderson Cancer Center, Houston TX. All experiments with cell lines were performed within 6 months after thawing or obtaining from ATCC or DSMZ. The cell lines were also authenticated in the Characterized Cell Line Core Facility at M.D. Anderson Cancer Center, Houston TX. HEL92.1.7, SET-2, and THP1 cells were cultured in ATCC-formulated RPMI-1640 media with 20% FBS and 1% penicillin/streptomycin. HS5 and HEK293T cells were cultured in high-glucose-formulated DMEM media with 10% FBS and 1% penicillin/streptomycin. Logarithmically growing, mycoplasma-negative cells were utilized for all experiments. Following drug treatments, cells were washed free of the drug(s) prior to the performance of the studies described. HEL-OTX P/R, SET-2-OTX P/R and THP1-OTX P/R cells were generated by culturing HEL92.1.7, SET-2, or THP1 cells in their LD_90_ concentration of OTX015 for 48 hours. Dead cells were removed by Ficoll Hypaque centrifugation. Live cells were washed once with complete media to remove residual Ficoll and cultured in complete media until viability was greater than 90% by trypan dye exclusion assessment. This process was repeated for a total of 10 shocks.

**Cell Line Authentication**. The cell lines utilized in these studies were authenticated in the Characterized Cell Line Core Facility at M.D. Anderson Cancer Center, Houston TX utilizing STR profiling.

**Primary AML blasts:** Patient-derived AML cells samples were obtained with informed consent as part of a clinical protocol approved by the Institutional Review Board of The University of Texas, M.D. Anderson Cancer Center. Normal hematopoietic progenitor cells (HPCs) were obtained from delinked, de-identified cord blood samples. Mononuclear cells were purified by Ficoll Hypaque (Axis Shield, Oslo, Norway) density centrifugation following the manufacturer’s protocol. Mononuclear cells were washed once with sterile 1X PBS and suspended in complete RPMI media containing 20% FBS and counted to determine the number of cells isolated prior to immuno-magnetic selection. CD34+ AML blast progenitor cells were purified by immuno-magnetic beads conjugated with anti-CD34 antibody following the manufacturer’s protocol (StemCell Technologies, Vancouver, British Columbia) prior to utilization in the cell viability assays, RNA expression, and immunoblot analyses.

**Primary post-MPN-MF secondary AML blasts:** Primary peripheral blood and/or bone marrow aspirate samples were obtained with informed consent from patients with secondary AML transformed from high risk (≥ 3) MF (according to the International Prognostic Scoring System, IPSS) or de novo AML under an IRB-sanctioned protocol. The samples were collected in heparinized tubes, and mononuclear cells were separated using Ficoll Hypaque (Axis Shield, Oslo, Norway) following the manufacturer’s protocol. The cells were washed once with sterile 1X PBS, then re-suspended in complete RPMI-1640, and counted to determine the number of cells isolated prior to immuno-magnetic selection. Secondary AML blast progenitor cells were purified by immuno-magnetic beads conjugated to anti-CD34 antibody (StemCell Technologies, Vancouver, British Columbia) prior to utilization in the cell viability assays.

**Sequencing of primary de novo and sAML blast cells:** We performed targeted next-generation sequencing (NGS) of DNA samples from bone marrow or peripheral blood collected from patients at our center with de novo AML or sAML transformed from MF (1). Diagnostic bone marrow samples were obtained for mutational analysis. Total genomic DNA was extracted from unenriched peripheral blood (PB) or bone marrow (BM) samples using ReliaPrep genomic DNA isolation kit (Promega Corp, Madison, WI, USA). Briefly, a total of 250 ng DNA was utilized to prepare sequencing libraries using Agilent HaloPlex custom Kit (Agilent Technologies, Santa Clara, CA, USA). The entire coding sequences of ABL1, ASXL1, BRAF, CALR, DNMT3A, EGFR, EZH2, FLT3, GATA1, GATA2, HRAS, IDH1, IDH2, KIT, KRAS, MDM2, IKZF2, JAK1, JAK2, MLL, MPL, MYD88, NOTCH1, NF1, NPM1, NRAS, PTPN11, RUNX1, TET2, TP53, and WT1 were interrogated on a custom-designed next-generation sequencing approach using the Illumina MiSeq platform (Illumina; San Diego, CA, USA; RRID:SCR_016379). The genomic reference sequence used was genome GRch37/hg19. The following software tools were utilized in the experimental setup and data analysis: Illumina Experiment Manager 1.6.0 (Illumina; San Diego, CA, USA), MiSeq Control Software 2.4 (Illumina; San Diego, CA, USA), Real Time Analysis 1.18.54 (Illumina; San Diego, CA, USA), Sequence Analysis Viewer 1.8.37 (Illumina; San Diego, CA, USA), MiSeq Reporter 2.5.1 (Illumina; San Diego, CA, USA), and SureCall 3.0.1.4 (Agilent Technologies; Santa Clara, CA, USA). A minimum of 80% reads at quality scores of AQ30 or higher were required to pass quality control. The lower limit of detection of this assay (analytical sensitivity) for single nucleotide variations was determined to be 5% (one mutant allele in the background of nineteen wild type alleles) to 10% (one mutant allele in the background of nine wild type alleles). Testing of patients with active hematologic malignancies was limited to somatic mutations only.

**Analysis of epigenetic state in AML cells *in vitro***. ATAC-Seq analysis of untreated and treated AML cells was performed following a previously described protocol (2)_._ ATAC-Seq libraries were generated with a Nextera DNA Library Preparation Kit containing the mutant Tn5 transposase (Illumina, San Diego, CA; Catalog number: FC-121-1030). The DNA fragments were indexed utilizing a Nextera Index Kit (Illumina, San Diego, CA; Catalog number: FC-121-1011) and amplified by PCR utilizing NEBNext® High-Fidelity 2X PCR Master Mix according to the manufacturer’s protocol (New England Biolabs, Ipswich, MA). Library fragments were amplified for 12-15 cycles utilizing the denaturation, annealing, and extension times as previously described (2). The amplified library fragments were PCR-purified with a Qiagen MinElute column (Qiagen, Germantown, MD) then size selected with a 1.0X bead concentration to remove fragments shorter than 200 bp. Library fragments were incubated with AMPure XP SPRI beads (Beckman Coulter, Indianapolis, IN) for 10 minutes at room temperature in 1.5 mL microcentrifuge tubes. The mixture was placed on a magnetic stand for 10 minutes. The supernatant was removed and the SPRI beads were washed twice with fresh 80% ethanol (30 seconds each wash) and air-dried for 2-3 minutes. Library DNA was eluted from the SPRI beads with a 20 µL volume of 10 mM Tris-HCl (pH 8.5). Beads were incubated at room temperature for 10 minutes, then the tubes were transferred to a magnetic stand for 10 minutes. The supernatant containing the DNA libraries was carefully removed by pipetting and transferred into a clean microcentrifuge tube. The individual libraries were quantified and quality-checked by Thermo Fisher Qubit [Thermo Fisher Qubit fluorimeter, RRID:SCR_018095] fluorometric quantification and Agilent Bioanalyzer 2100 [Agilent 2100 Bioanalyzer Instrument, RRID:SCR_019389] analysis, respectively. Individual libraries were pooled into one tube, purified over a Qiagen MinElute column [QIAGEN, RRID:SCR_008539], eluted in 20 µL of 10 mM Tris, (pH 8.5) and sequenced on a NextSeq 500 next generation sequencer (Illumina NextSeq 500, RRID:SCR_014983) utilizing a 150 cycle mid-output kit (Illumina, San Diego, CA). Raw sequencing data was mapped using TopHat2 (3, 4) [TopHat, RRID:SCR_013035] onto the human genome build UCSC hg19 (NCBI 37) for human data and log2-fold changes were calculated with diffReps (5) [diffReps, RRID:SCR_010873]. Sequence tracks were visualized with IGV software [Integrative Genomics Viewer, RRID:SCR_011793]. We also determined the H3K27Ac status and BRD4 occupancy in untreated and treated OCI-AML5 cells by ChIPmentation following a previously described protocol (6), with modifications on the concentration of AmpPure XP beads utilized for dual AmpPure XP SPRI bead selection of the final libraries. We utilized 0.65X beads for the first selection, then a 1.0X bead concentration to narrow the fragment size of the final tagmented ChIP DNA library. ChIP input DNA libraries were only selected with a 1.0X bead concentration. The individual libraries (ChIP and input) were quantified and quality-checked with Qubit and Bioanalyzer 2100 analysis, respectively. The libraries were pooled into one tube, purified utilizing a Qiagen MinElute column, and eluted in 20 µL for loading onto a NextSeq500 sequencer utilizing a mid-output kit. Raw sequence data were mapped to UCSC hg19 (NCBI 37) and log2 fold-changes were calculated with diffReps (5) [diffReps, RRID:SCR_010873]. Sequence tracks were visualized with IGV software (7, 8) [RRID:SCR_011793]. To identify super enhancers, we performed a ranked order of super enhancers (ROSE) analysis [ROSE, RRID:SCR_017390] utilizing the H3K27Ac status of the chromatin according to the methods of Loven et al. (9). Analysis of transcription factor binding motifs in gained ATAC-Seq peaks was performed with HOMER [HOMER, RRID:SCR_010881].

**Transcriptome Analysis.** Total RNA was isolated from untreated or LSD1 inhibitor-treated AML and sAML cells utilizing a PureLink RNA Mini kit from Ambion, Inc. (Austin, TX). Sequencing libraries were prepared with ERCC spike-in controls in the MD Anderson Cancer Center DNA Sequencing and Microarray core facility and sequenced on an Illumina HiSeq-4000 next generation sequencer [Illumina HiSeq 3000/HiSeq 4000 System, RRID:SCR_016386]. Each library yielded 30-40 million read pairs. Data was mapped using TopHat2 [RRID: SCR_013035] (3, 4) onto the human genome build UCSC hg19 (NCBI 37) for human data. Gene expression was assessed using Cufflinks2 (4) [Cufflinks, RRID: SCR_014597], then variance stabilization and quantile normalization were applied. Significantly altered transcripts were determined using the limma package (10) in R [LIMMA, RRID: SCR_010943]; multiple hypotheses testing correction was applied using the false discovery rate (fdr) method implemented in the R statistical system. We considered that significance was achieved for fold changes greater than or equal to 1.25X up or down relative to the untreated or parental cells, and p-values less than 0.05. We inferred enriched pathways using the Gene Set Enrichment (GSEA) method (11), and the gene set collection from the Molecular Signature Database (MSigDB) (12) [Molecular Signatures Database, RRID:SCR_016863].

**Detection of enhancer RNAs in AML cells**. To determine the abundance and changes in enhancer RNA due to LSD1 knockout in AML cells, we utilized the sequencing reads from H3K27Ac ChIP Seq and from RNA-Seq loaded into IGV software. Within known super enhancer/enhancer regions, we screened for regions of overlap with high H3K27Ac signal in the ChIP tracks and lower abundance reads in the RNA-Seq signal tracks. These regions were selected and utilized for designing primers for qPCR with the Primer3 online software tool (<http://primer3.ut.ee/>) (13) [Primer3, RRID:SCR_003139]. Two non-overlapping primer sets were generated for each potential enhancer RNA. Primer sets were tested by qPCR on a StepOnePlus™ Real-Time PCR System [StepOnePlus Real-Time PCR System, RRID:SCR_015805] utilizing SYBR-Green PCR mastermix (Cat # 4309155, Thermo Fisher) and cDNA that had been generated with random hexamers from control and LSD1-knockout cells. A melt curve analysis was performed to ensure that primer pairs generated only a single product in qPCR. The abundance of enhancer RNA in control and LSD1 knockout cells from independent experiments was normalized to the expression of GAPDH.

**Short hairpin RNAs for LSD1 and GFI1 in AML cells.** A pool of shRNAs against KDM1A/LSD1 and GFI1 were obtained from Sigma Aldrich (KDM1A: SHCLNG-NM_015013 and GFI1: SHCLNG-NM_005263). After determining the most effective shRNAs for LSD1 knockdown in a constitutive state in AML cells, the two most active shRNAs were synthesized as long oligonucleotides from sequences obtained from the Broad shRNA Consortium. A non-targeting shRNA was also utilized. The oligos were annealed to each other by boiling in annealing buffer (10 mM Tris, pH 8.0, 50 mM NaCl, 1 mM EDTA) then performing a step-down reaction 3°C per cycle to room temperature utilizing a thermocycler (Bio-Rad). The annealed oligos were ligated at 16°C overnight with a tetracycline-inducible vector that had been digested with *Age1* and *EcoRI* and gel purified. The ligated DNA was transformed into *E. coli* Stbl3 cells (Invitrogen, Carlsbad, CA). Tet-pLKO-puro was a gift from Dmitri Wiederschain (Addgene plasmid # 21915; http://n2t.net/addgene:21915; RRID:Addgene_21915). Positive transformants were checked by restriction digestion with *XhoI* and confirmed by Sanger sequencing before proceeding to lentiviral production and transduction of AML cells. OCI-AML5 and MOML13 cells were transduced with the inducible shRNA vectors, maintained in Tet-free FBS-containing media and selected with puromycin for 96 hours to isolate stable transduced cell lines. Cell lines were treated with doxycycline at 100 ng/mL daily to induce and maintain action of the shRNA for all experiments. After determining the most effective shRNAs for GFI1 knockdown in a constitutive state in OCI-AML5cells, the two most active shRNAs were utilized for the remaining studies following lentiviral transduction and selection with 0.5 µg/mL of puromycin for 72 hours to isolate positive transductants.

**Plasmid Generation, Viral Packaging, and Creation of Cell Lines.** Plasmid constructs for the production of lentivirus were transfected with packaging plasmids psPAX2 and pMD2.G into HEK-293T cells utilizing jetPRIME reagent (PolyPlus Transfection, New York, NY). The psPAX2 and pMD2.G packaging plasmids were a gift from Didier Trono (Addgene plasmid #12260 and #12259 [RRID:Addgene_12260; RRID: Addgene_12259]). Media was changed the following day. Viral supernatant was collected 72 hours post transfection and filtered through a 0.45 µm PES membrane. AML and sAML cells were seeded at 5 x 10^5^ cells/mL in a 50:50 mix of media and lentiviral supernatant with 8 µg/mL polybrene (Sigma-Aldrich). The following day, the viral supernatant was removed by centrifugation and cells were transduced with fresh viral supernatant for an additional 24 hours. To generate luciferase-expressing OCI-AML5, HEL92.1.7, and HEL-OTX P/R cells, pHIV-Luc-ZsGreen (a gift from Bryan Welm [Addgene plasmid #39196;http://n2t.net/addgene:39196; RRID:Addgene_39196]) was packaged as above and transduced into OCI-AML5, HEL92.1.7, or HEL-OTX P/R cells. ZsGreen-positive cells were sorted by flow cytometry (FACSAria, FL-1 channel, top 10% brightest GFP-expressing cells), and expanded in culture prior to their utilization in therapeutic in vivo mouse studies. To generate AML cells (OCI-AML5) with stable expression of SP-Cas9, lentiCas9-Blast (a kind gift from Feng Zhang; Addgene #52962; RRID:Addgene_52962) was utilized. Transduced cells were selected with 8-10 µg/mL of blasticidin for 10 days. Expression of Cas9 in AML cells was confirmed by immunoblot analyses utilizing anti-FLAG M2 (Sigma-Aldrich) and anti-Cas9 antibodies.

**CRISPR/Cas9-mediated gene editing in cultured AML and sAML cells.** To study the effects of knockout of LSD1 or GFI1 in AML cells, the CHOP-CHOP prediction algorithm (14) was utilized to develop guide RNAs. Guide RNAs were developed against exon 2 and exon 3 sequence of GFI1. For LSD1, guide RNAs were developed against sequences within the intron splice site between exon 2/3 and between exon 3/4. High scoring sgRNAs were synthesized by Synthego, Inc or DNA oligos were synthesized, annealed and cloned. sgRNAs for use in OCI-AML5-Cas9 cells were cloned into digested LRG [Lenti_sgRNA_EFS_GFP][RRID:Addgene_65656] vector and cells were transduced with lentivirus, and sorted by FACS for GFP-positive cells. For sAML SET-2 cells without stable Cas9 expression, to obtain Cas9-sgRNA RNPs (ribonucleoprotein complexes), 1 μg of synthetic sgRNA was incubated with 1.5 μg recombinant Cas9 protein (Synthego, Inc.) for 15 min at room temperature. sAML SET-2 cells were transfected by electroporation utilizing the Amaxa Nucleofector device with Cell Line-Specific Nucleofector Kit V (Amaxa GmbH, Cologne, Germany) as per the manufacturer's instructions and program X-005. Immediately post-transfection, cells were plated in complete media containing no antibiotics and 2 µM of ROCK inhibitor (Y-27632, Selleck Chemicals; Houston, TX) and allowed to recover for 24 hours. Knockout of LSD1 (OCI-AML5 and SET-2) or GFI1 (OCI-AML5) was confirmed by mRNA expression by qPCR 5-6 days post-transfection and Western blot analysis 7-8 days post transfection/transduction. Gene-edited AML and sAML cells were treated with OTX015 or ruxolitinib (sAML cells) for 48 hours and the % of Annexin V-positive, apoptotic cells were determined by flow cytometry.

**CRISPR/Cas9-mediated gene editing in patient-derived, post-MPN sAML blasts.** For LSD1 knockout, guide RNAs were developed against sequences within the intron splice site between exon 2/3 and between exon 3/4. High scoring sgRNAs were synthesized by Synthego, Inc. To obtain Cas9-sgRNA RNPs (ribonucleoprotein complexes), 1 μg of synthetic sgRNA was incubated with 1.5 μg recombinant Cas9 protein (Synthego, Inc.) for 15 min at room temperature. Following RNP incubation, 7 x 10^6^ post-MPN sAML cells were transfected by electroporation utilizing a nucleofector kit-V (Lonza Bioscience, Basel, Switzerland) and protocol (T-030) on an Amaxa Nucleofector II device (Lonza Bioscience). Transfected cells were treated with 2 µM of ROCK inhibitor (Selleck Chemicals. Houston, TX), plated on a GFP-expressing HS5 stromal cell monolayer and incubated for 5 days. The viability of the gene-edited cells was tracked by trypan blue dye uptake. Cells were also treated with ruxolitinib for 48 hours to determine the effects of knockout of LSD1 on sensitivity to ruxolitinib.

**Generation of a dTAG13-responsive LSD1-FKBP12^F36V^-HA expressing AML cell line.** To generate an in-frame fusion between the LSD1 cDNA (NM_015013) and the F36V mutant FKBP12 cDNA, an LSD1 cDNA clone (Cat #SC125274) was obtained from Origene (Rockville, MD). The mutant FKBP12 cDNA was amplified from pET15b His6-FKBPF36V which was a gift from Thomas Wandless (Addgene plasmid #73180) [RRID:Addgene_73180]. An attB1 site was incorporated into the N-terminus of the primer for LSD1 (attB1 Lsd1.for 5’- GGGG **ACA AGT TTG TAC AAA AAA GCA GGC TTC** ACC ATGG ATG TTA TCT GGG AAG AAG GCG GCA G and a *Not1* restriction site into the reverse primer (NotI LSD1.rev 5’- GTC A **GCG GCC GC** CAT GCT TGG GGA CTG CTG TGC-3’), thus removing the native stop codon. A *Not1* restriction site was designed into the forward primer of the FKBP12 cDNA (NotI FKBP12.for 5’-GTC A **GCG GCC GC**T GGA GTG CAG GTG GAA ACC ATC TC-3’) and a C-terminal HA-tag and attB2 site were designed into the reverse primer (attB2 HA FKBP12.rev 5’-GGGG **AC CAC TTT GTA CAA GAA AGC TGG GTA** TCA AGC GTA ATC TGG AAC ATC GTA TGG GTA AGC GTA ATC TGG AAC ATC GTA TGG GTA TTC CAG TTT TAG AAG CTC CAC ATC GAA GAC-3’). The cDNAs were amplified by PCR using Pfusion polymerase according to the manufacturer’s recommendations for temperature and extension times. PCR products were column-purified and digested with *Not1* overnight at 37°C. The resulting fragments were gel purified and ligated with T4 DNA ligase overnight at 16°C. The pDONR^TM^221 vector (Invitrogen, Carlsbad, CA) was utilized for the BP clonase reaction. The LSD1-FKBP12 fusion DNA was introduced into the Gateway cloning vector pDONR^TM^221 following the manufacturer’s BP clonase protocol and incubating the reaction at room temperature for 2 hours. The recombined DNA was transformed into *E. coli* TOP10 cells and selected with 100 µg/mL of ampicillin. Transformants were checked by DNA sequencing. The fusion cDNA was transferred by Gateway cloning into pLEX_305, a kind gift from David Root (Addgene # 41390)[RRID:Addgene_41390] utilizing an LR clonase reaction. The resulting plasmid was transformed into *E. coli* DH5 alpha cells and selected with 100 µg/mL of ampicillin. Positive clones were confirmed by DNA sequencing and then pLEX_305/LSD1-FKBP12^(F36V)^-HA vector was combined with packaging vectors (as above) to generate lentiviral particles in HEK293T cells. OCI-AML5 cells that had been previously engineered to stably express SP-Cas9 (as above) were transduced with lentivirus supernatants and selected with 0.5 µg/mL of puromycin for 96 hours. Next, two splice-blocking sgRNAs were used as above: one in the intron between exon 2 and exon 3, and one in the intron between exon 3 and exon 4. DNA oligos were synthesized, annealed to each other in annealing buffer, then ligated into digested LRG [Lenti_sgRNA_EFS_GFP] vector, a kind gift from Christopher Vakoc (Addgene #65656) [RRID: Addgene_65656] and transformed into *E. coli* Sure2 cells (Part Number: 200152; Agilent Technologies, Santa Clara, CA ). Positive transformants were confirmed by Sanger sequencing, then combined with packaging vectors to generate lentiviral particles. Lentiviral supernatant was combined with OCI-AML5 LSD1- FKBP12^(F36V)^-HA expressing cells and incubated for 48 hours. GFP-positive cells were selected by FACS sorting and expanded for experimentation. dTAG-13 was synthesized as previously described (15). Cells were treated with dTAG-13 (kindly provided by Dr. Nathanael Gray) as indicated.

**Cell proliferation analysis**. For cell proliferation analysis, cells were plated in triplicate at 0.125 x 10^6^ cells/mL and total cell numbers were counted utilizing a Countess-2 cell counting instrument (Life Technologies, Carlsbad, CA) every 24 hours for 72-96 hours.

**Cell cycle analysis of AML cells**. After the designated treatments, cells were harvested by centrifuging at 125 x g for 5 minutes. Cells were washed twice with 1× phosphate-buffered saline (PBS) in 12 x 75 flow tubes, re-suspended in 200 µL of 1X PBS and fixed in 70% ethanol by adding 800 µL of molecular grade 70% ethanol dropwise to the cells in the tube. The tubes were then vortexed to mix and stored overnight at -20°C. Fixed cells were washed twice with 1× PBS by centrifuging at 125 x g for 5 minutes and then stained in 250 µL of DNA staining buffer [5 mL Triton-PBS (100 µL of Triton X100 in 100 mL of 1X PBS) with 100 µL of 1 mg/mL propidium iodide and 100 µL of 10mg/mL RNAse A] in the dark for 15 minutes at 37°C. Cell-cycle data were collected on a flow cytometer with a 488 nM laser in the FL-2 channel and analyzed with Accuri CFlow6 software (BD Biosciences).

**Assessment of apoptosis by annexin-V staining.** Untreated or drug-treated cells were stained with Annexin-V (Pharmingen, San Diego, CA) and TO-PRO-3 iodide (Life Technologies, Carlsbad, CA) and the percentages of apoptotic cells were determined by flow cytometry. To analyze synergism, cells were treated with combinations for 48 hours and the percentages of annexin V-positive, apoptotic cells or % PI-positive, non-viable cells were determined by flow cytometry. The combination index (CI) for each drug combination was calculated by median dose effect and isobologram analyses (assuming mutual exclusivity) utilizing the commercially available software CompuSyn (16). CI values of less than 1.0 represent a synergistic interaction of the two drugs in the combination. The CI values were input into GraphPad V8.0 to create the Box plots of the range of the CI values for each cell line and combination studied. We also utilized matrix dosing of agents in combinations to allow synergy assessment by Bliss scoring utilizing the SynergyFinder V2 online web application tool (<http://synergyfinder.fimm.fi/>) (17, 18).

**Assessment of percentage non-viable cells.** Following designated treatments, PD, CD34+ sAML cells were stained with trypan blue (Sigma, St. Louis, MO) and counted on a Countess-2 cell counting instrument (Life Technologies, Carlsbad, CA). The numbers of non-viable cells were also determined by counting the cells that exhibited trypan blue uptake in a hemocytometer, and were reported as a percentage of the untreated control cells. Alternatively, cells were washed with 1X PBS, stained with propidium iodide or TO-PRO-3 iodide (Life Technologies, Carlsbad, CA) and analyzed by flow cytometry on a BD Accuri CFlow-6 flow cytometer (BD Biosciences, San Jose, CA).

**Assessment of leukemia cell differentiation.** Following knockout or knockdown of LSD1 by shRNA in AML cells or treatment with LSD1 inhibitors, cells were harvested and washed with 1X PBS. Cells were re-suspended in 0.5% BSA/PBS and stained with FITC-conjugated anti-CD86 antibody [RRID:AB_396012] and APC-conjugated anti-CD11b antibody [RRID:AB_398456] or FITC-conjugated IgG1 isotype control [RRID:AB_396090] and APC-conjugated IgG1 isotype control antibody [RRID:AB_398613] in the dark, at 4°C for 15-20 minutes. Cells were washed with 0.5% BSA/PBS by centrifugation at 125 x g for 5 minutes, and then re-suspended in 0.5% BSA/PBS for analysis by flow cytometry. Similar staining was also performed with anti-CD117(c-Kit)-APC conjugated antibody (BD Biosciences Cat# 561118, [RRID: AB_10562384]). Cells were assessed in the FL-1 and FL-4 fluorescence channels. Differentiation of leukemia cells was also determined by examination of cellular/nuclear morphology. Cells were cytospun onto glass slides at 500 rpm for 5 minutes. The cytospun cells were fixed and stained with a Protocol® HEMA3 stain set (Fisher Scientific, Kalamazoo, MI). Cellular/nuclear morphology was assessed by light microscopy. Two hundred cells were counted in at least 5 sections of the slide for each condition. The % morphologic differentiation is reported relative to control cells.

**Colony growth of AML and sAML cells**. OCI-AML5, OCI-AML2, THP1, SET-2, and HEL92.1.7 cells were treated with INCB059872 for 96 hours. Then, cells were stained with 0.4% Trypan blue and viable cells were counted with a Countess II counting device. Five hundred cells were plated in complete Methocult media into wells of a 12-well plate and incubated at 37°C. Colony growth was assessed after 7-10 days.

**RNA isolation and quantitative polymerase chain reaction.** Following the designated treatments, total RNA was isolated from sAML cells utilizing a PureLink RNA Mini kit from Ambion, Inc. (Austin, TX) and reverse transcribed with a High Capacity Reverse Transcription kit from Life Technologies (Carlsbad, CA). Quantitative real-time PCR analysis for the expression of target genes was performed on cDNA using TaqMan probes and a TaqMan Universal PCR Mastermix from Applied Biosystems (Foster City, CA). Relative mRNA expression was normalized to the expression of GAPDH and compared to the untreated cells.

**Cell lysis and protein quantitation.** Untreated or drug-treated cells were centrifuged, and the cell pellets were incubated in lysis buffer on ice for 20 minutes (19). After centrifugation, an aliquot of each cell lysate was diluted 1:10 and the protein content was quantitated using a BCA protein quantitation kit (Pierce, Rockford, IL), according to the manufacturer’s protocol. Protein concentrations were determined by comparing the absorbance at 562 nm compared to a known concentration range of bovine serum albumin (BSA) from 0.125 mg to 2 mg/mL.

**SDS-PAGE and immunoblot analyses.** Seventy five micrograms of total cell lysate were used for SDS-PAGE. Western blot analyses were performed on total cell lysates using specific antisera or monoclonal antibodies. Blots were washed with 1× PBST, then incubated in IRDye 680RD goat anti-mouse (RRID:AB_10956588) or IRDye 800CW goat anti-rabbit (RRID:AB_621843) secondary antibodies (LI-COR, Lincoln, NE) for 1 h, washed three times in 1× Phosphate Buffered Saline with Tween®20 (PBST) and scanned with an Odyssey CLX Infrared Imaging System utilizing Image Studio 5.0 Software (RRID:SCR_015795) (LI-COR, Lincoln, NE). The expression levels of β-Actin or GAPDH in the cell lysates were used as the loading control for the western blots. Immunoblot analyses were performed at least twice. Representative immunoblots were subjected to densitometry analysis. Densitometry analysis was performed using ImageJ software (20).

**LSD1-GFI1 ELISA Assay in AML cells.** To determine the impact of LSD1 inhibitor on the binding of LSD1 and GFI1 in AML cells, a sandwich ELISA assay was utilized. Rabbit polyclonal anti-LSD1 antibody (Diagenode) was coated onto wells of an ELISA plate overnight at 4°C. The wells were washed 4X with ELISA Wash Buffer from a DuoSet ELISA Ancillary Reagent Kit (Catalog #DY008, R&D systems, Minneapolis, MN). Fifty micrograms of total cell lysate from untreated and drug treated cells was added and the reactions were incubated at room temperature for 4 hours. Cell lysate was removed and the wells were washed 4X with ELISA wash buffer. Mouse monoclonal anti-GFI1 antibody was added and the reaction was incubated for 2 hours at room temperature. The wells were washed 4X with ELISA wash buffer, then HRP-conjugated anti-mouse secondary antibody was added and the reactions were incubated at room temperature for 1 hour in the dark. The wells were washed 4X with ELISA wash buffer and developer solution was added. The wells were incubated at room temperature in the dark for 30 minutes, then Stop solution was added and the absorbance at 450 nm and 540 nm (background) were recorded on a plate reader.

**CRISPR domain-scanning dropout screen in OCI-AML5 cells:** To determine specific dependencies in AML cells and identify sgRNA dropouts that were synthetic lethal with treatment with LSD1 inhibitors, we obtained a human chromatin regulatory domain-focused CRISPR screening library, in which sgRNAs had been designed to target the catalytic domain or bromodomain of each protein in the library based on the NCBI database annotation, from the laboratory of Dr. Christopher Vakoc (Cold Spring Harbor Laboratory, Cold Spring Harbor, New York). The library of sgRNAs (Table S1) was sub-cloned into *E. coli* Stbl3 cells. Lentivirus was prepared as described above. OCI-AML5-Cas9 expressing cells were transduced with the library in duplicates for 24 hours. Cells were washed, centrifuged at 125 x g, and plated in complete media. Forty eight hours post-transduction, a portion of the cells were removed and frozen for genomic DNA isolation. The remaining cells were cultured for an additional 6 days. On day 8, cells were split into treatment groups (in duplicate) and treated for 96 hours with LSD1 inhibitor. Twelve days post-transduction, live AML cells were harvested by ficoll density centrifugation, and genomic DNA was isolated with a GeneJET Genomic DNA Purification Kit (Thermo Scientific). Primers surrounding the sgRNA sequence were utilized to minimally amplify (20 cycles) the genomic DNA from the Day2-transduced cells and the Day12 untreated and INCB-treated cells. Libraries were constructed and amplicon-seq was performed on a MiSeq next-generation sequencer. Sequencing reads were used to determine the read counts of the remaining sgRNAs in each condition. Log2 fold-changes in the sgRNA reads between Day12 and Day2 or between Day12 treated and untreated groups were calculated utilizing the CRISPRCloud2 online software application (21). Dropouts were graphed with GraphPad V8 software.

**In vivo model of de novo AML and post-MPN, secondary AML:** All in vivo studies were approved by, and conducted in accordance with the guidelines of the IACUC at the M.D. Anderson Cancer Center, an AAALAC-accredited facility. Male and female NOD.Cg-Prkdc^scid^ Il2rg^tm1Wjl^/SzJ (NSG) mice (stock number: 005557; 4-6 weeks of age) [Jackson Labs, Bar Harbor, ME; RRID: IMSR_JAX:005557] were exposed to 2.5 Gy of radiation. The following day, mice (n=10 per cohort) were injected in the lateral tail vein with 3.0 x 10^6^ GFP-luciferase expressing OCI-AML5 or 2.0 x 10^6^ GFP-luciferase expressing HEL92.1.7 or HEL-OTX P/R cells and monitored for 4-5 days. Mice were imaged utilizing a Xenogen Lumina in vivo imaging system to document engraftment before treatment was initiated. Mice were randomized into groups based on equivalent mean bioluminescent intensity to control for variation in cell engraftment and variation between different treatment groups. Treatments were initiated on day 5. For the OCI-AML5 model, mice were treated with 1.5 mg/kg of INCB059872 daily for 4 weeks by oral gavage. Mice were imaged weekly by bioluminescent imaging to document treatment efficacy and/or disease progression. Total bioluminescence was recorded as photons/second. Mice that became moribund or experienced hind limb paralysis were euthanized according to the approved IACUC protocol. Department of Veterinary Medicine staff members assisting in determining when euthanasia was required were blinded to the experimental conditions of the study. The survival of the mice is represented by a Kaplan-Meier plot. Significance was determined by a Mantel-Cox log rank test. P-values of less than 0.05 were assigned significance. For the HEL92.1.7 mouse model, mice were treated with vehicle, INCB059872 (1.5 mg/kg, daily x 5 days, by oral gavage), and/or ruxolitinib (20 mg/kg, daily x 5 days, by oral gavage), or OTX015 (30 mg/kg, daily x 5 days, by oral gavage) for 3 weeks and then treatment was stopped. All mice in each treatment cohort were imaged utilizing a Xenogen Lumina in vivo imaging system once per week to monitor disease status and treatment efficacy. Total bioluminescence was recorded as photons/second. Mice that became moribund or experienced hind limb paralysis were euthanized according to the approved IACUC protocol. Department of Veterinary Medicine staff members assisting in determining when euthanasia was required were blinded to the experimental conditions of the study. The survival of the mice is represented by a Kaplan-Meier plot. Significance was determined by a Mantel-Cox log rank test. P-values of less than 0.05 were assigned significance. For the HEL-OTX P/R mouse model, mice were treated with INCB059872 (1.5 mg/kg, daily x 5 days, by oral gavage) for one week followed by three weeks of treatment with INCB059872 (1.5 mg/kg, daily x 5 days, by oral gavage) and/or OTX015 (50 mg/kg daily x 5 days, by oral gavage). All mice in each treatment cohort were imaged utilizing a Xenogen Lumina in vivo imaging system once per week to monitor disease status and treatment efficacy. Total bioluminescence was recorded as photons/second. Mice that became moribund or experienced hind limb paralysis were euthanized according to the approved IACUC protocol. The survival of the mice is represented by a Kaplan-Meier plot. Significance was determined by a Mantel-Cox log rank test. P-values of less than 0.05 were assigned significance.

**Power analysis for in vivo studies**. With a sample size of 10 mice per group, we can achieve 79.5% power to detect a difference of overall survival at a significance level of 0.05 with one-sided log-rank test, assuming 30% of mouse-survival at the end of study in the experimental group.

**Statistical analysis**. Significant differences between values obtained in AML or sAML cells treated with different experimental conditions compared to untreated control cells were determined using the Student’s t-test in GraphPad V8. For the *in vivo* mouse models, a two-tailed, unpaired t-test was utilized for comparing total bioluminescent flux. For survival analysis, a Kaplan-Meier plot and a Mantel–Cox log rank test were utilized for comparisons of different cohorts. P values of < 0.05 were assigned significance.

**Data and Software availability**. RNA-Seq, ChIP-Seq, and ATAC-Seq datasets have been deposited in GEO as a super series under accession ID GSE160319.

**REFERENCES for Supplemental Methods**

1. Khan M, et al. Clinical outcomes and co-occurring mutations in patients with runx1-mutated acute myeloid leukemia. *Int J Mol Sci* **18**, (2017).
2. Buenrostro JD, Wu B, Chang HY, Greenleaf WJ. ATAC-seq: A method for assaying chromatin accessibility genome-wide. *Curr Prot Mol Biol*. **109**, 21 29 21-29, (2015)
3. Kim D, et al. TopHat2: accurate alignment of transcriptomes in the presence of insertions, deletions and gene fusions. *Genome Biol*. **14**, R36, (2013).
4. Trapnell C, et al. Transcript assembly and quantification by RNA-Seq reveals unannotated transcripts and isoform switching during cell differentiation. *Nat Biotechnol* **28**, 511-5, (2010).
5. Shen L, Shao NY, Liu X, Maze I, Feng J, Nestler EJ. diffReps: detecting differential chromatin modification sites from ChIP-seq data with biological replicates. *PLoS One* **8**, e65598 (2013).
6. Schmidl C, Rendeiro AF, Sheffield NC, Bock C. ChIPmentation: fast, robust, low-input ChIP-seq for histones and transcription factors. *Nat Methods* **12**, 963-5, (2015).
7. Robinson JT, et al. Integrative genomics viewer. *Nat Biotechnol* **29**, 24-6, (2011).
8. Thorvaldsdottir H, Robinson JT, Mesirov JP. Integrative Genomics Viewer (IGV): high-performance genomics data visualization and exploration. *Briefings in Bioinformatics* **14**, 178-92, (2013).
9. Loven J, Hoke HA, Lin CY, Lau A, Orlando DA, Vakoc CR, et al. Selective inhibition of tumor oncogenes by disruption of super-enhancers. *Cell* **153**, 320-34, (2013).
10. Smyth GK. Linear models and empirical bayes methods for assessing differential expression in microarray experiments. *Statistical applications in genetics and molecular biology* **3**, Article3, (2004).
11. Subramanian A, et al. Gene set enrichment analysis: a knowledge-based approach for interpreting genome-wide expression profiles. *Proc Natl Acad Sci USA* **102**, 15545-50, (2005).
12. Liberzon A, Subramanian A, Pinchback R, Thorvaldsdottir H, Tamayo P, Mesirov JP. Molecular signatures database (MSigDB) 3.0. *Bioinformatics*. **27**, 1739-40, (2011).
13. Untergasser A, et al. Primer3--new capabilities and interfaces. *Nucleic Acids Res* **40,** e115, (2012).
14. Labun K, Montague TG, Gagnon JA, Thyme SB, Valen E. CHOPCHOP v2: a web tool for the next generation of CRISPR genome engineering. *Nucleic Acids Res* **44**, W272-6, (2016).
15. Nabet B, et al. The dTAG system for immediate and target-specific protein degradation. *Nat Chem Biol* **14**, 431-441, (2018).
16. Chou TC, Talalay P. Quantitative analysis of dose-effect relationships: the combined effects of multiple drugs or enzyme inhibitors. *Advances in Enzyme Reg* **22**, 27-55, (1984).
17. Ianevski A, He L, Aittokallio T, Tang J. SynergyFinder: a web application for analyzing drug combination dose-response matrix data. *Bioinformatics* **33**, 2413-2415, (2017).
18. Ianevski, A., Giri, A. K., and Aittokallio, T. SynergyFinder 2.0: visual analytics of multi-drug combination synergies, *Nucleic Acids Res* **48**, W488-W493, (2020).
19. Fiskus W, Verstovsek S, Manshouri T, Rao R, Balusu R, Venkannagari S, et al. Heat shock protein 90 inhibitor is synergistic with JAK2 inhibitor and overcomes resistance to JAK2-TKI in human myeloproliferative neoplasm cells. *Clin Cancer Res* **17**,7347-58, (2011).
20. Schneider CA, Rasband WS, Eliceiri KW. NIH Image to ImageJ: 25 years of image analysis. *Nat Methods* **9**, 671-5, (2012).
21. Jeong HH, Kim SY, Rousseaux MWC, Zoghbi HY, Liu Z. CRISPRcloud: a secure cloud-based pipeline for CRISPR pooled screen deconvolution. *Bioinformatics* **33**, 2963-2965, (2017).
